# Tracking funding disparities in global health aid with machine learning

**DOI:** 10.1038/s41467-026-76542-z

**Published:** 2026-08-18

**Authors:** Finn Stürenburg, Kerstin Forster, Nicolas Banholzer, Malte Toetzke, Kenneth Harttgen, Stefan Feuerriegel

**Affiliations:** 1 LMU Munich, Munich, Germany; 2 Munich Center for Machine Learning, Munich, Germany; 3 Institute of Social and Preventive Medicine, University of Bern, Bern, Switzerland; 4 Department of Public Health, University of Copenhagen, Copenhagen, Denmark; 5 TU Munich, Munich, Germany; 6 Max Planck Institute for Innovation and Competition, Munich, Germany; 7 ETH Zurich, Zurich, Switzerland

**Keywords:** Public health, Developing world

## Abstract

Reducing the global burden of disease is crucial for improving health outcomes. However, misalignment between health aid and country-level disease burden leaves vulnerable populations without the necessary support for major health challenges, particularly in the least developed countries. Here, we develop a machine learning pipeline using large language models to track flows in official development assistance (ODA) earmarked for health and identify aid–burden misalignment. We classified 3.7 million development aid projects from 2000 to 2022 (USD  ~ 332 billion) into 17 major categories of communicable, maternal, neonatal, and nutritional diseases (CMNNDs) and non-communicable diseases (NCDs). We compared the rank of per capita ODA disbursement against the rank of disease burden, measured in disability-adjusted life years (DALYs). We interpret DALY-based alignment as a policy-relevant heuristic rather than a prescriptive allocation criterion. Although funding and disease burden are significantly correlated for many diseases, there are notable disparities. For example, NCDs account for 59.5% of global DALYs but received only 2.5% of health-related ODA over the study period. This is concerning because low- and middle-income countries face an increasing double burden from both CMNNDs and NCDs. Our results show aid–burden misalignment across multiple diseases in several regions, including Central Africa and parts of South Asia and West Africa. Overall, our results identify health disparities to potentially inform policy decisions on development assistance and support targeted allocation of health aid.

## Abstract (web summary)

Misalignment between health aid and country-level disease burden can leave populations without the necessary support for major health challenges. Here the authors report a machine learning analysis of global health aid and conclude that aid is often misaligned with country-level disease burden, with non-communicable diseases receiving limited aid despite their large global burden.

## Introduction

Progress in global health has slowed since 2015^1–5^. As of now, only one out of the five health-related Sustainable Development Goals is on track for 2030^6,7^, with low- and middle-income countries facing large setbacks. These countries are confronted by a double burden of both severely under-resourced health systems^8,9^ and high disease burden from both communicable, maternal, neonatal, and nutritional diseases (CMNNDs) and non-communicable diseases (NCDs). For example, new HIV infections are three times above the 2025 goal^10^, progress on reducing tuberculosis (TB) deaths is less than a third of the target^11^, and cases for malaria have increased instead of reaching the reduction target for 2025^12^. Without faster action, none of the six WHO regions will meet the 2030 mortality targets for NCDs^1^. Recent cuts to health aid – particularly by major donors such as the United States – are further widening the gap between health needs and available resources^13–16^.

Official development assistance (ODA; hereafter referred to as aid) provides a key source of funding to address global health challenges, particularly in the least developed countries but also lower-middle-income and upper-middle-income countries that lack sufficient domestic resources^17–21^. However, substantial variation exists in how ODA is distributed across diseases and regions, leading to persistent gaps between funding needs and available resources^22–30^. Yet, systematic evidence on whether aid aligns with disease burden is limited. The Institute for Health Metrics and Evaluation’s Financing Global Health series provides comprehensive trends in development assistance for health from 1990–present across broad health areas, including non-communicable and neglected tropical diseases, and incorporates private foundation and NGO flows^31^, but does not provide granular country-level assessments of ODA allocations relative to disease burden. Other existing databases are typically constrained to only a few common diseases (e.g., typically only HIV/AIDS, TB, malaria and maternal and neonatal health^32–39^) and often lack comprehensive temporal and geographic coverage (e.g., the focus is often limited exclusively to Africa or a few selected countries such as the Countdown countries)^32–35^. Most analyses precede 2020 and thus do not reflect the impact of the COVID-19 pandemic. Moreover, the aforementioned works aimed at tracking and analyzing ODA flows rely heavily on manual coding, which is not scalable and limits timely and comprehensive evidence for policy decision-making.

**Fig. 1:**
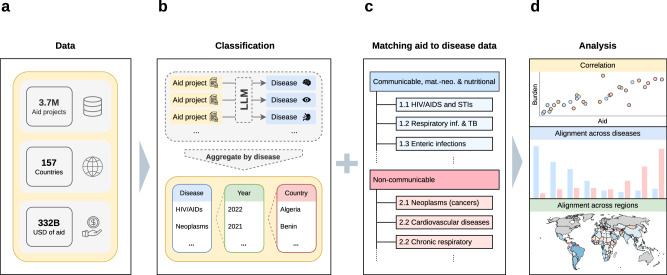
Machine learning pipeline to track aid–burden alignment in global health. Our machine learning pipeline tracks country-level disparities between aid flows and disease burden across major diseases and regions. **a** The input consists of project descriptions from 3.7 million official development assistance (ODA) projects worldwide. **b** We used an open-source large language model (Llama-3.1-70B-Instruct) to perform multi-label classification of aid projects into disease categories. We focus on 17 major causes of disease burden, grouped into communicable, maternal, neonatal, and nutritional diseases (CMNNDs) and non-communicable diseases (NCDs), as defined by the Global Burden of Disease study^40,41^. Funding was then aggregated by disease, year, and country. **c** We matched the disease-specific ODA to country-level disease burden, measured in disability-adjusted life years (DALYs) from the Global Burden of Disease study^40,41^. **d** The pipeline enables analysis of the alignment between ODA flows and disease burden (e.g., via Spearman’s rank correlation coefficient). Together, this approach offers a scalable way to monitor aid--burden alignment and allows for regular updates to inform evidence-based policies in global health. Icons were obtained from Flaticon (https://www.flaticon.com/): database by Smashicons, internet by LAFS, donate by surang, add file by itim2101, brain by Irfansusanto20, eye by Gregor Cresnar, stomach by brick-stock. Country boundaries are from Natural Earth, 1:110m Admin 0 Countries (public domain).

Here, we develop a machine learning pipeline using large language models to track ODA flows for public health (see Fig. 1). This enables us to analyze the global allocation of ODA earmarked to CMNNDs and NCDs and assess whether the distribution of funding is in line with the country-specific disease burden. Using our machine learning pipeline, we classified 3.7 million ODA projects from 2000 to 2022 (~USD 332 billion across 157 recipient countries) into 17 major categories of CMNNDs and NCDs. We focus on donor contributions (ODA disbursements) and not domestic government or household health spending. We then assessed the alignment between disease-specific ODA flows and disease burden across major causes as defined by the Global Burden of Disease study^40,41^. Specifically, we compared the rank of per capita ODA disbursement against the rank of disease burden (i.e., disability-adjusted life years [DALYs]) to identify relative aid–burden misalignment at the country level. We interpret aid–burden misalignment as a descriptive measure that highlights divergence, not as a prescriptive criterion for optimal funding allocation, given legitimate alternative objectives and acknowledging that DALY-based metrics reflect specific value assumptions^42,43^. Our machine learning pipeline is scalable and allows for regular updating. It supports evidence-based policies in global health by identifying health needs and informs the targeted allocation of health aid to reduce the global burden of diseases.

## Results

### Disease classification of aid projects

We analyzed 3.7 million development aid projects from 2000–2022 as provided by the Creditor Reporting System^44^ of the Organisation for Economic Co-operation and Development (OECD), which is the standardized reporting system for donors in the development sector across the world. Using an open-source large language model (Llama-3.1-70B-Instruct), we classified all projects into 17 causes of disease burden as defined by the Global Burden of Disease study^40,41^. Our categorization distinguishes (i) CMNNDs (e.g., HIV/AIDS and sexually transmitted infections [STIs], respiratory infections and TB, nutritional deficiencies) and (ii) NCDs (e.g., mental disorders, neoplasms, substance use disorders). The full list of disease categories is provided in Supplementary Table S1. We validated the classification from our machine learning approach by comparing it against (i) a keyword-based classification approach and (ii) manual annotations of 2060 projects (see Supplementary Material S1), which confirms the accuracy of our machine learning pipeline. Of the 3.7 million projects, our machine learning pipeline classified 321,527 as being related to one of the major diseases (8.7%), while the remaining projects relate to other sustainability targets (e.g., climate action). We then aggregated the ODA flows across disease, year, and country. To contextualize these flows, we summarized the distribution of funding by donor (Supplementary Table S17) and found that global health ODA is dominated by a small number of countries and international funds as donors, and their funding decisions may have the largest impact on reducing the aid–burden disparities identified in this analysis.

Throughout the analyses, we use DALY burden as one policy-relevant heuristic for assessing how closely ODA disbursements track disease burden across health categories. This heuristic is not intended as a stand-alone criterion of appropriate allocation. Departures from DALY burden may reflect other important considerations, including cost-effectiveness assessments, domestic financing capacity, health-system constraints, equity concerns, and strategic donor or recipient priorities^42,45,46^. We thus interpret DALY alignment as one of several decision-relevant reference points for the analysis rather than as a prescriptive standard. Because our data capture donor disbursements only, and do not incorporate domestic financing, private donations, or household spending, the observed aid–burden alignment should not be interpreted as a measure of overall underfunding. Rather, it indicates how closely ODA disbursements track disease burden across diseases and countries.

### Distribution of global aid earmarked to health

**Fig. 2:**
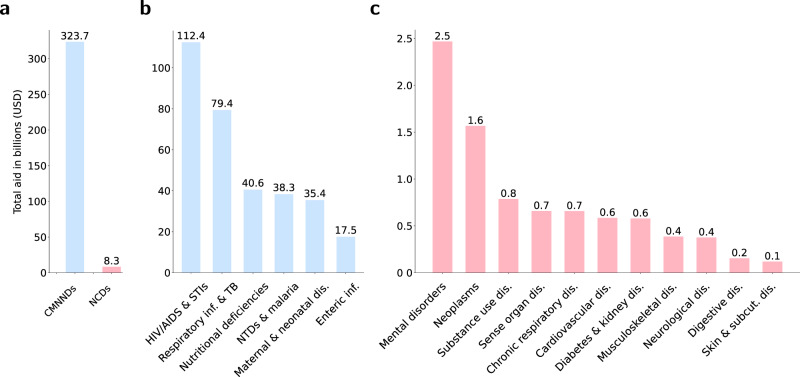
Distribution of total development aid (2000–2022) across diseases. Shown is (**a**) the aggregate comparison of total aid between CMNNDs and NCDs, alongside total aid for **b**, CMNNDs and **c**, NCDs. The majority of funding was allocated to CMNNDs, particularly HIV/AIDS and STIs, and respiratory infections and TB. The categorization of diseases follows the Global Burden of Disease study^40,41^. Abbreviations: CMNNDs, communicable, maternal, neonatal, and nutritional diseases; NCDs, non-communicable diseases; STIs, sexually transmitted infections; TB, tuberculosis; NTDs, neglected tropical diseases. Source data are provided as a Source Data file.

The distribution of ODA shows large disparities between CMNNDs and NCDs (Fig. 2). From 2000 to 2022, a total of USD 332 billion was allocated to disease-specific aid projects, of which 97.5% (USD 323.7 billion) targeted CMNNDs. In contrast, only 2.5% (USD 8.3 billion) was directed toward NCDs. Most funding was concentrated in a few areas, with HIV/AIDS and STIs alone receiving USD 112.4 billion and respiratory infections and TB receiving USD 79.4 billion. By comparison, NCDs received considerably less (e.g., cardiovascular diseases: USD 0.6 billion; diabetes including kidney disorders: USD 0.6 billion; and mental disorders: USD 2.5 billion), despite growing prevalence in low- and middle-income countries^8,9,40^.

**Fig. 3:**
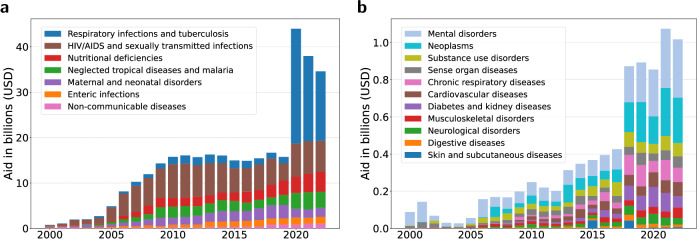
Temporal trends in annual global health aid across diseases (2000–2022). Shown are annual aid disbursements by disease category for (**a**) CMNNDs and (**b**) NCDs. Overall, annual aid increased from USD 0.78 billion in 2000 to USD 34.60 billion in 2022, with notable peaks in funding for respiratory infections and TB during the COVID-19 pandemic. Abbreviations: CMNNDs, communicable, maternal, neonatal, and nutritional diseases; NCDs, non-communicable diseases; TB, tuberculosis. Source data are provided as a Source Data file.

Between 2000 and 2022, global health funding increased from USD 783 million per year to USD 34.6 billion per year (Fig. 3; overall aid in this time period: USD 332 billion), which corresponds to a more than 40-fold increase. However, funding priorities shifted between 2000 and 2022. For example, between 2000 and 2022, funding for HIV/AIDS and STIs rose from USD 174 million to USD 6.9 billion, though their share of total aid declined over time; funding for nutritional deficiencies rose from USD 93 million to USD 4.3 billion; and funding for neglected tropical diseases and malaria rose from USD 77 million to USD 3.6 billion. Further, a large increase is seen in ODA earmarked for respiratory infections and TB during the COVID-19 pandemic, which reached an annual volume of USD 25.3 billion in 2020, before declining to USD 15.3 billion by 2022.

**Fig. 4:**
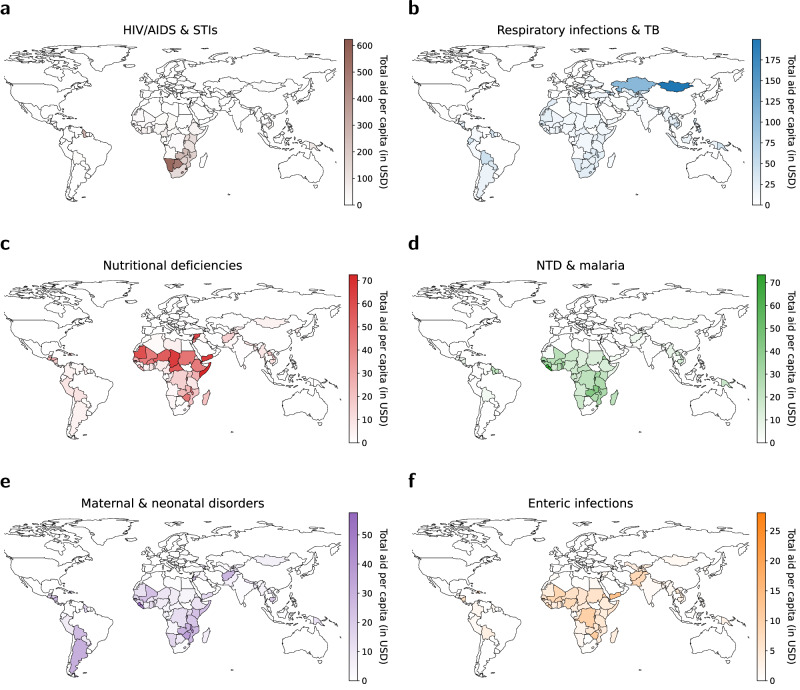
Geographic variation of global health aid (for CMNNDs). Maps show accumulated disease-specific ODA per capita (in USD) across countries from 2000 to 2022. The plots show (**a**) HIV/AIDS and STIs; **b** respiratory infections and TB; **c** nutritional deficiencies; **d** NTDs and malaria; **e** maternal and neonatal disorders; and (**f**) enteric infections. Darker colors indicate higher per capita aid; white indicates countries with no recorded allocation of aid. Country boundaries are from Natural Earth, 1:110m Admin 0 Countries (public domain). Abbreviations: CMNNDs, communicable, maternal, neonatal, and nutritional diseases; ODA, official development assistance; STIs, sexually transmitted infections; TB, tuberculosis; NTDs, neglected tropical diseases. Source data are provided as a Source Data file.

**Fig. 5:**
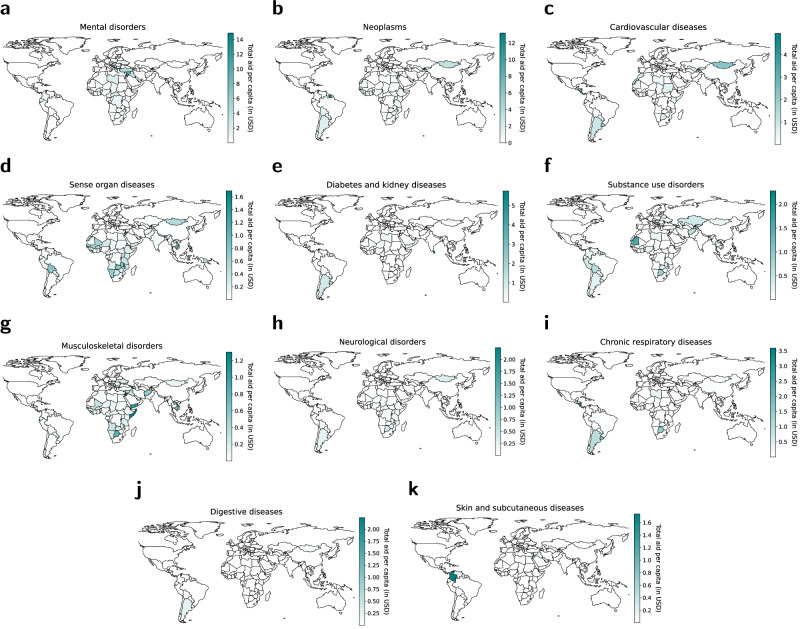
Geographic variation of global health aid (for NCDs). Maps show accumulated disease-specific ODA per capita (in USD) across countries from 2000 to 2022. Darker colors indicate higher per capita aid; white indicates countries with no recorded allocation of aid. The plots show (**a**) mental disorders; **b** neoplasms; **c** cardiovascular diseases; **d** sense organ diseases; **e** diabetes and kidney diseases; **f** substance use disorders; **g** musculoskeletal disorders; **h** neurological disorders; **i** chronic respiratory diseases; **j**, digestive diseases; and (**k**) skin and subcutaneous diseases. Country boundaries are from Natural Earth, 1:110m Admin 0 Countries (public domain). Abbreviations: NCDs, non-communicable diseases; ODA, official development assistance. Source data are provided as a Source Data file.

Over 2000–2022, accumulated disease-specific ODA per capita varies substantially across countries for both CMNNDs (Fig. 4) and NCDs (Fig. 5). For CMNNDs, ODA for HIV/AIDS and STIs is highest in Southern Africa (e.g., Eswatini: USD 623, Namibia: USD 557, Botswana: USD 495); ODA for respiratory infections and TB is highest in Central Asia and the Caucasus (e.g., Mongolia: USD 200, Kazakhstan: USD 105, Georgia: USD 80); ODA for nutritional deficiencies is highest in the Horn of Africa, the Middle East, and the Sahel (USD 50–110); ODA for neglected tropical diseases and malaria is highest in West Africa (USD 55–75). ODA for maternal and neonatal disorders is more broadly distributed, with notable per capita highs in South Sudan and Sierra Leone (USD 58–72). ODA for enteric infections is generally lower, typically below USD 30 per capita. For NCDs, ODA per capita is generally lower than for CMNNDs and more concentrated in a limited set of countries, with minimal disease-specific funding elsewhere.

### Alignment between aid and disease burden

**Fig. 6:**
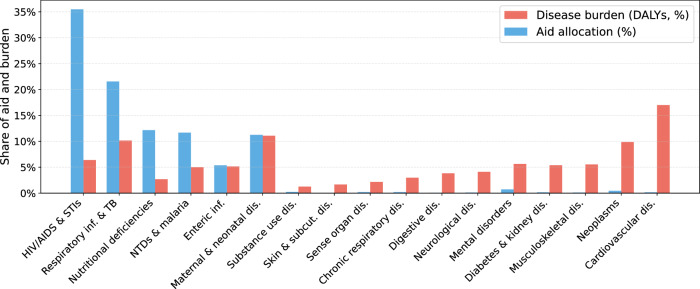
Disparities between aid volume and disease burden. Shown are the proportion of total health aid allocated to each disease category (in blue) and the proportion of the accumulated global disease burden (in red). Total health aid is based on the accumulative disbursement from 2000 to 2021. Disease burden is based on DALYs from 2000 to 2021 using the latest available data from the Global Burden of Disease study at the time of our analysis. Deviations between these proportions point to disparities; that is, diseases receiving less aid relative to their burden may reflect misalignment of funding relative to burden, while those receiving more may reflect historical or donor-driven priorities. Abbreviations: DALYs, disability-adjusted life years; STIs, sexually transmitted infections; TB, tuberculosis; NTDs, neglected tropical diseases. Source data are provided as a Source Data file.

Our analysis points to substantial disparities between the proportion of allocated ODA for each disease and the corresponding proportion of the global disease burden (Fig. 6). While such disparities may be driven by unobserved factors (e.g., historical or donor-driven priorities, political economy), they point to aid–burden misalignment, whereby some diseases receive less aid relative to their share of global disease burden. We interpret aid–burden misalignment as a descriptive measure of divergence rather than as evidence of misallocation: first, departures from aid–burden alignment may reflect legitimate priorities (e.g., equity, contagion control, or system strengthening). Second, DALY aggregation—blending severity and prevalence—embeds value choices that shape interpretation^42^. Accordingly, we treat aid–burden alignment as a decision-relevant reference point rather than a prescriptive criterion for optimal funding allocation. We find that HIV/AIDS and STIs, neglected tropical diseases and malaria, and nutritional deficiencies received disproportionately high ODA volumes compared to the respective disease burden. For example, HIV/AIDS and STIs account for 6% of the global disease burden, but received 34% of total health aid, and similarly large disparities are present for neglected tropical diseases and malaria (5% of burden vs. 12% of aid) and nutritional deficiencies (3% of burden vs. 12% of aid). In contrast, NCDs account for 59.5% of the overall disease burden but received only a small fraction of global health aid (2.5%). Such disparities are especially pronounced, for instance, for cardiovascular diseases (17% of burden vs. <1% of aid), diabetes and kidney diseases (5.4% of burden vs. <1% of aid), neoplasms (10% of burden vs. <1% of aid), and mental disorders (6% of burden vs. <1% of aid), which received low funding despite the substantial negative impact on global health.

**Fig. 7:**
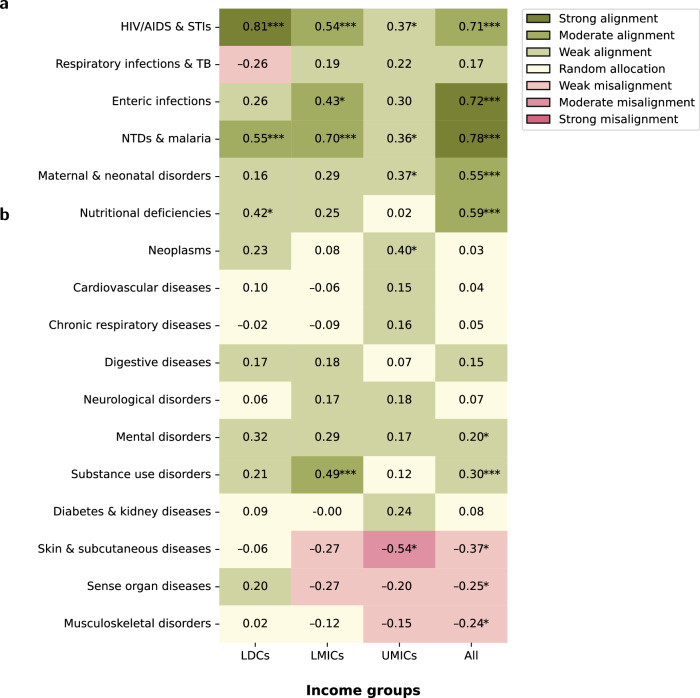
Correlation between disease-specific aid and burden across income groups. Shown are Spearman’s rank correlation coefficients (*ρ*) between per capita aid (accumulated over the time period 2000--2021) and disease burden across country income groups: least developed countries (LDCs), lower-middle-income countries (LMICs), upper-middle-income countries (UMICs), and all countries combined. **a** The top six rows report CMNNDs, which generally show positive correlations across most income groups. **b** In contrast, NCDs (bottom eleven rows) exhibit more heterogeneity in the correlations. Green indicates positive correlations and red indicates negative correlations. Asterisks denote statistically significant correlations based on two-sided Spearman rank tests, where *p* denotes the corresponding *p*-value (^*^p < 0.05; ^**^p < 0.01; ^***^p < 0.001). Legend interpretation: −1 to −0.7: strong misalignment of health aid with disease burden; −0.7 to −0.3: moderate misalignment; −0.3 to −0.1: weak misalignment; −0.1 to 0.1: indicative of random allocation; 0.1 to 0.3: weak alignment, 0.3 to 0.7, moderate alignment; and 0.7 to 1.0: strong alignment. Abbreviations: CMNNDs, communicable, maternal, neonatal, and nutritional diseases; NCDs, non-communicable diseases; STIs, sexually transmitted infections; TB, tuberculosis; NTDs, neglected tropical diseases. Source data are provided as a Source Data file.

To analyze how well aid allocation between countries is aligned with country-level disease burden, we analyzed the correlation between per capita aid and disease burden using Spearman’s rank correlation coefficient (Fig. 7). These correlations reveal how closely funding aligns with needs across diseases and country income groups (i.e., least developed countries [LDCs], lower-middle-income countries [LMICs], and upper-middle-income countries [UMICs], as per the country income group categorization of the OECD^44^). Overall, CMNNDs show stronger and more consistent positive correlations compared to NCDs (Fig. 7, right column). For instance, HIV/AIDS and STIs (*ρ* = 0.71; *p* < 0.001) and NTDs and malaria (*ρ* = 0.78; *p* < 0.001) exhibit strong alignment. In contrast, several NCDs show negative correlations (i.e., skin and subcutaneous diseases, sense organ diseases, and musculoskeletal disorders), meaning that greater disease burden is associated with less aid. Results are robust to using disease incidence instead of DALYs: correlations between incidence and disease-specific per capita aid show largely consistent alignment patterns (Supplementary Fig. S3).

We also observe substantial variation across income groups (Fig. 7, columns 1–3). For example, HIV/AIDS and STIs show a strong positive correlation in all income groups, particularly in LDCs. In contrast, respiratory infections and TB show weaker or even negative correlations in lower-income settings. When restricting the analysis to the COVID-19 period (2020–2021), the overall alignment patterns remain similar (Supplementary Figs. S4–S6), although respiratory infections and TB show reduced alignment with disease burden. This reflects the temporary disruption in funding patterns during the pandemic. On the other hand, excluding the pandemic years for respiratory infections and TB markedly strengthens the correlation (*ρ* = 0.63; *p* < 0.001; Supplementary Figs. S7–S9), which suggests that the COVID-19 aid surge substantially affected alignment for these diseases. Together, these patterns highlight that aid allocation across countries seems well targeted for CMNNDs in lowest-income settings, but also point to persistent disparities in funding for many NCDs.

### Geographic distribution of aid–burden misalignment

**Fig. 8:**
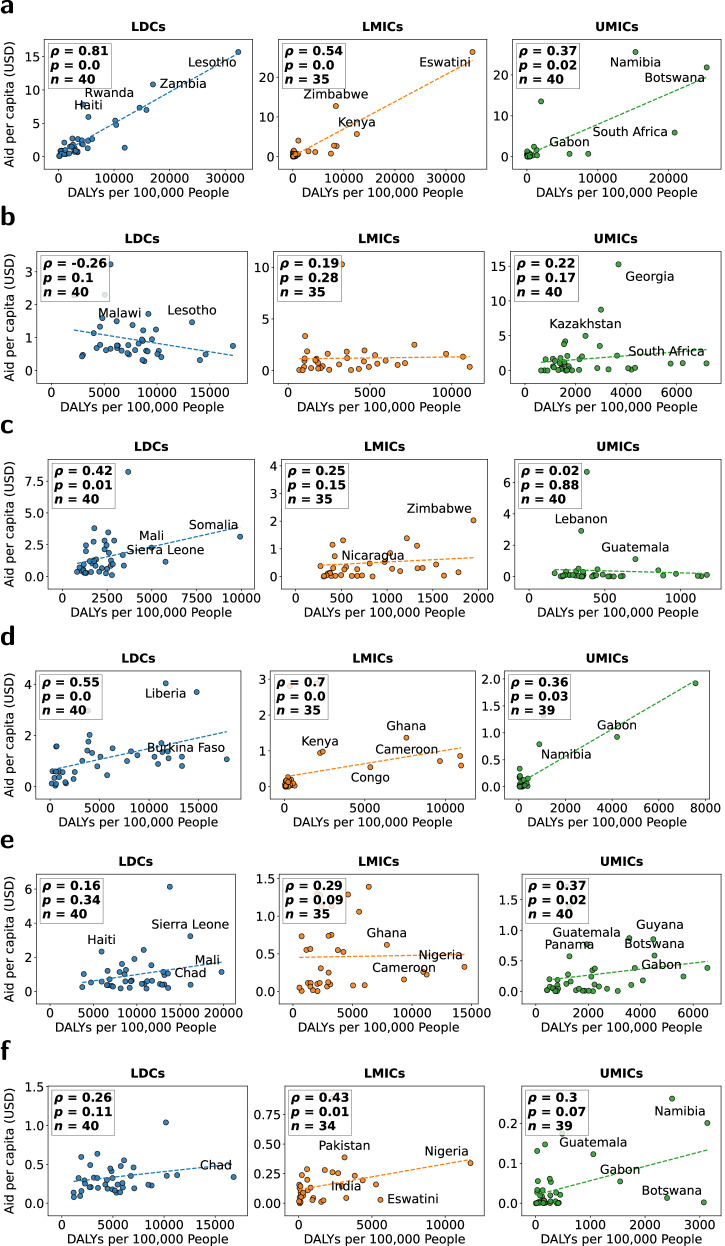
Funding disparities at the country level (for CMNNDs). The plots compare disease burden (*x*-axis, measured by DALYs per 100,000 population, accumulated for 2000--2021) against aid per capita (*y*-axis, accumulated for 2000--2021). Each point represents one country, which allows identifying countries with high disease burden but comparatively low aid and thus to reveal aid--burden misalignment. For each disease, countries/plots are grouped by country income groups: least developed countries (LDCs), lower-middle-income countries (LMICs) and upper-middle-income countries (UMICs). We focus on selected diseases with high overall funding: **a** HIV/AIDS and STIs; **b** respiratory infections and TB; **c** nutritional deficiencies; **d** NTDs and malaria; **e** maternal and neonatal disorders; and (**f**) enteric infections. Each plot reports Spearman’s rank correlation coefficient (*ρ*), the corresponding *p*-value from a two-sided Spearman rank test, and the number of countries included in the analysis (*n*). The dashed line is the fitted trend line from an ordinary least squares regression. Funding disparities for NCDs are presented in Supplementary Fig. S1. Abbreviations: CMNNDs, communicable, maternal, neonatal, and nutritional diseases; NCDs, non-communicable diseases; DALYs, disability-adjusted life years. Source data are provided as a Source Data file.

To locate potential aid–burden misalignment at the country level, we plot the country-level patterns of disease burden against aid per capita (see Fig. 8 for CMNNDs and Supplementary Fig. S1 for NCDs). This highlights countries with a mismatch between ODA flows and disease burden, that is, where the disease burden is substantially higher (or lower) than per capita aid. For example, in the case of HIV/AIDS and STIs, the Central African Republic, classified as a least developed country in the OECD CRS database, ranks at the 90th percentile for disease burden, but is only at the 52nd percentile for per capita ODA volume of all LDCs. Funding for NCDs shows similar disparities (Supplementary Fig. S1).

**Fig. 9:**
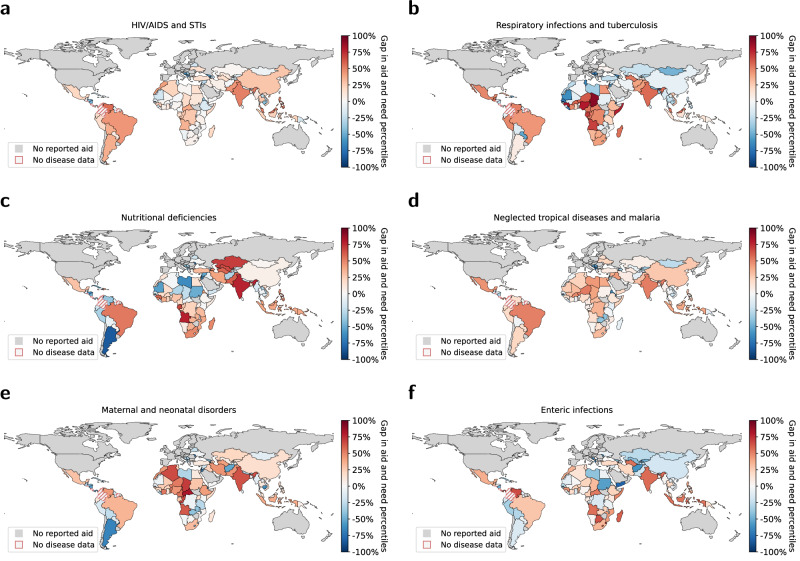
Geographic distribution of aid–burden misalignment (for CMNNDs). The maps show the relative aid--burden misalignment by disease. Here, we refer to “aid--burden misalignment” as the difference between a country’s percentile in disease burden (DALYs per 100,000 population) and its percentile in per capita aid, calculated within income groups. Aid--burden misalignment captures relative disparity within income groups, not necessarily absolute underfunding. In other words, an aid--burden misalignment of 10 percentiles means that the country’s disease burden is 10 percentiles higher than its aid level. Put simply, assuming 100 peer countries, this country would need to climb 10 places in the aid rank to have a similar rank in terms of aid and burden. Due to space, we focus on selected diseases with high overall funding: **a** HIV/AIDS and STIs; **b** respiratory infections and TB; **c** nutritional deficiencies; **d** NTDs and malaria; **e** maternal and neonatal disorders; and (**f**) enteric infections. Red color denotes aid--burden misalignment, where a country’s aid rank is lower than its burden rank. Blue color denotes that a country’s aid rank is higher than the burden rank. Gray indicates no reported aid; red striped areas indicate no disease data. The geographic distribution of aid--burden misalignment for NCDs is shown in Supplementary Fig. S2. Country boundaries are from Natural Earth, 1:110m Admin 0 Countries (public domain). Abbreviations: CMNNDs, communicable, maternal, neonatal, and nutritional diseases; NCDs, non-communicable diseases; DALYs, disability-adjusted life years; STIs, sexually transmitted infections; TB, tuberculosis. Source data are provided as a Source Data file.

We find regions with aid allocated disproportionately to burden (see Fig. 9 for CMNNDs and Supplementary Fig. S2 for NCDs). Several regions show substantial aid–burden misalignment across multiple diseases, notably in Central Africa, West Africa, and parts of South Asia. Here, we refer to “aid–burden misalignment” as the difference between a country’s percentile rank in disease burden (DALYs per 100,000 population) and its percentile rank in per capita aid, calculated within income groups to account for potential heterogeneity in macroeconomic factors such as the price of labor and the general state of health systems. Aid-burden misalignment captures relative disparity within income groups, not necessarily absolute underfunding. For example, countries from Southern Africa exhibit aid–burden misalignment for enteric infections, nutritional deficiencies, respiratory infections and TB, maternal and neonatal disorders, but these countries appear relatively well funded for HIV/AIDS and STIs, which reflects historic donor priorities in these regions^47–50^. The geographic distribution of aid–burden misalignment for NCDs is shown in Supplementary Fig. S2.

## Discussion

Our analysis shows a skew in aid toward communicable diseases, with LDCs and LMICs exhibiting a stronger aid-burden alignment than UMICs in these disease categories. One potential reason for this is that the greater domestic capacity of UMICs to manage disease burden was reflected in donor priorities. HIV/AIDS in particular has consistently received the largest share of global health aid prior to the COVID-19 pandemic, reflecting long-standing prioritization by international donors^38^. Communicable diseases offer clearer, shorter-term impact paths, fitting donors’ preference for vertical, high-visibility results^51^. However, such priorities may displace funding for other important areas of healthcare^52,53^, including NCDs, which continue to receive a disproportionately low share of ODA relative to their growing global burden^54^. Furthermore, NCDs would often involve systemic and multisectoral approaches that would benefit multiple disease categories simultaneously. While the WHO and international donor organizations agreed in 2007 that health-system strengthening should become a greater priority^55–57^, our analysis indicates that funding disparities still reflect historical priorities, suggesting that general improvements to global health systems in years prior to the COVID-19 pandemic need to continue. However, amid recent cuts to WHO and USAID funding, which threaten progress in HIV, malaria, maternal health, and other disease categories^51,57,58^, retaining and reallocating scarce resources equitably will become even more difficult. These developments underscore the necessity for timely and comprehensive monitoring of health aid flows to ensure investments align with evolving global health needs.

We found a considerable increase in the funding for respiratory infections and tuberculosis during the COVID-19 pandemic. While emergency funding surged and the introduction of infection control measures may have provided broader benefits for infectious disease control^59^, many low-income countries experienced sharp disruptions in routine health services^60,61^, including tuberculosis case detection, HIV testing and treatment initiation, and malaria prevention campaigns^62–65^. Low-income countries would thus have needed an increase in aid for several disease categories to cope with the additional burden of the pandemic. However, aid for diseases unrelated to respiratory infections did not show a significant increase or decrease, suggesting that the pandemic did not trigger a broader shift in funding priorities. Furthermore, our sensitivity analysis revealed that the correlation between disease burden and aid became lower for respiratory infections and tuberculosis during the 2020–2021 period (Supplementary Figs. S4–S6), suggesting that the COVID-19 pandemic exposed and possibly exacerbated inequities in development aid allocation. The impact of the pandemic was especially severe in low-income countries, with regions such as sub-Saharan Africa facing a “triple burden” of tuberculosis, HIV, and COVID-19^66^. Fragile health systems and aid misalignments left underserved populations even more vulnerable and the disparities in health aid and infrastructure contributed to an unequal recovery, which may increase global health inequities in the years following the pandemic.

While HIV/AIDS has consistently received the largest share of ODA prior to the COVID-19 pandemic, ODA distribution is uneven, and our analysis showed substantial geographical disparities in the alignment between HIV/AIDS-related ODA and disease burden. TB, despite its long-standing burden, continues to attract considerable funding, yet eradication remains challenging due to persistent poverty and the lack of an effective vaccine^11^. Notably, the Global Fund’s recent change in allocation methodology, increasing the share of funds for TB in high-burden countries from 18% to 22%, represents a positive shift^67^. At the same time, the burden of NCDs is rising rapidly in many low- and middle-income countries, with increasing prevalence of conditions like cardiovascular diseases and mental disorders^68^. Yet, we found that pronounced aid–burden misalignment remains for many NCDs. In sum, our results point to persistent relative misalignment between CMNNDs and NCDs: aid remains misaligned with their respective burden shares, even as NCD-directed ODA has risen over time. This indicates challenges in adapting to slow structural changes.

Our machine learning pipeline offers several strengths. First, it generates a comprehensive, fine-grained dataset of ODA flows across 157 countries and 17 disease categories. As a result, our dataset provides substantially broader coverage across diseases and regions as compared to prior efforts, which typically focus on a limited set of diseases (e.g., HIV/AIDS, TB, and malaria^32–39,69^) and specific geographical contexts (e.g., certain African countries^32–35^). Second, our pipeline leverages a state-of-the-art, open-source large language model, which enables accurate and consistent classification of health-related ODA. By making our code publicly available, we also allow for seamless adoption by end users across various institutional settings. Third, our machine learning pipeline is scalable, thus allowing for regular updates and timely monitoring of ODA flows. It helps address the delays and resource demands associated with manual coding and strengthen the evidence available for policy-making in global health.

As with others, our study is subject to limitations. First, the textual descriptions of ODA projects vary in quality, specificity, and length, which may introduce bias into the classification process. In addition, a fraction of projects lacked any textual description and could therefore not be categorized by our machine learning pipeline and are excluded from the analysis. For example, diseases such as HIV and TB are linked; therefore, many projects may relate to both, and it is challenging to attribute the relative proportion of funding each disease received. Nevertheless, these descriptions provide raw, first-hand data and offer greater granularity compared to earlier efforts, which strengthens the accuracy and depth of our findings. Moreover, previous studies have shown that such data are highly effective for monitoring tasks^70^, which corroborates the robustness of our analysis. Second, the reliability of our findings depends on the performance of the underlying machine learning pipeline. For example, translating non-English descriptions via machine learning may introduce translation error, and classification error may affect estimates of aid–burden misalignment. To mitigate this risk, we evaluated our approach using multiple validation and robustness analyses, including (i) comparison with a conventional keyword-based classification approach, (ii) manual annotations for 2060 ODA projects, and (iii) robustness checks using a 1% subset of disease-category projects, thereby providing evidence for the reliability of our approach. In the validation set for the robustness analyses, rare categories were represented by relatively few observations, which may increase the uncertainty of category-specific performance estimates for these categories. At the same time, although inter-annotator agreement was high, a small number of disagreements involved broad or cross-cutting health projects, indicating some residual ambiguity in the underlying project descriptions and category boundaries. In addition, classification performance may vary across alternative LLMs, but the performance was relatively similar for different state-of-the-art LLMs that we compared as part of our robustness checks. We therefore interpret the validation and robustness results as supporting the overall reliability of the pipeline, including robust category-specific performance estimates for most disease categories, while acknowledging residual uncertainty for a small number of rare categories. While the validation set was constructed in line with established best practice for informative validation under high inter-annotator agreement^71^, it remains limited and may not fully capture the full range of text structures and other corpus characteristics. Furthermore, state-of-the-art LLMs may be sensitive to input structure, including the position of relevant information within the text and how inputs are processed relative to the model’s context window^72,73^. These factors may influence classification performance in subtle ways and may therefore affect the overall accuracy of our analyses.

Third, we focus on various causes of disease burden as defined by the Global Burden of Disease study^40,41^ to enable comparability over time but which may limit responsiveness to emerging global health priorities that fall outside the existing taxonomy. Nevertheless, our pipeline can be seamlessly adapted to incorporate additional disease categories as needed. Moreover, because some projects are assigned to single primary disease categories, cross-disease interactions and co-benefits (e.g., HIV–TB) can impact estimates of aid–burden misalignment, with the magnitude likely varying by disease area. Projects outside the disease categories (e.g., education, environment, climate) may have health spillovers that we do not attribute to specific diseases, which can shift apparent aid–burden alignment in either direction. Fourth, while we analyze ODA at the disease-category level, we do not disaggregate funding by regions beyond the country level, donors, or funding instruments, which may mask variations in aid effectiveness within categories. We also restrict our analysis to ODA as reported by the Creditor Reporting System of the OECD. Although CRS provides comprehensive coverage of bilateral and multilateral government donors and includes major institutional and philanthropic actors (such as the Global Fund and the Bill & Melinda Gates Foundation), it has only partial coverage of other private philanthropic contributions; accordingly, our findings primarily reflect government-sourced and large institutional development assistance for health, rather than the full landscape of privately financed global health funding. We track disbursements as reported in the CRS database and do not trace downstream reallocations, which introduces uncertainty in disease-specific estimates and may not fully capture end-use spending relative to the reported allocations. When a project covers multiple disease areas, we apportion funding equally following prior work^24,27,32,37^, which may not fully reflect the actual allocation. Fifth, our analysis assesses the alignment between disease burden and aid allocation, rather than directly estimating the financial resources required to achieve specific health targets or the adequacy of total health financing. Importantly, our data cover ODA disbursements but not domestic financing, private donations, or household spending. Other methods are therefore needed to quantify the financial requirements associated with reaching specific targets. Sixth, our analysis focuses on DALY burden as a benchmark for comparing aid allocation across health categories. While DALY-based comparisons provide a policy-relevant way to assess how closely ODA disbursements track disease burden^26,74^, this benchmark is not a standalone criterion of appropriate allocation. DALYs aggregate severity and prevalence into a single summary measure, thus involving inherent value choices that may affect the interpretation of misalignment. Departures from DALY alignment may reflect other legitimate allocation priorities, such as cost-effectiveness assessments, domestic financing capacity, health-system constraints, equity concerns, and strategic donor or recipient priorities^42,45,46^. Observed patterns of aid–burden alignment should therefore be interpreted as decision-relevant reference points rather than as prescriptive allocation criteria.

Addressing the persistent gap between global health needs and ODA requires timely, disease-specific evidence to inform policy decisions. Our machine learning pipeline enables systematic and scalable tracking of the alignment between ODA flows and disease burden and can be regularly updated to reflect newly available data and emerging health priorities. Our approach thereby supports more targeted, equitable allocation of ODA by aligning funding with health needs and improving health outcomes worldwide.

## Methods

This study complies with all relevant ethical regulations. The study was based exclusively on secondary, non-identifiable data: project-level records of official development assistance from the OECD Creditor Reporting System and aggregate country-level disease burden estimates from the Global Burden of Disease study. The study did not involve human participants, human biological material, clinical data, individual-level personal data, identifiable images, interventions, or the collection of data from individuals. The use of all third-party data complied with the terms and conditions of the respective data providers.

### Data

We obtained data on ODA projects from the Development Co-operation Directorate of the OECD, which provided us with raw data from the Creditor Reporting System (CRS)^44^. The CRS tracks international ODA projects and is considered the most comprehensive data source on global development aid^75^. In particular, the coverage of the CRS is more comprehensive compared to other datasets on health-related aid, which are often limited to specific diseases, geographic regions, or lack granular disease categorizations. We focus on donor contributions (ODA disbursements) and not domestic government or household health spending.

The ODA projects in the CRS cover various forms of development assistance, including financial grants, development loans, and equity investments made by donor organizations. Projects are reported directly by donor organizations at the close of each year in which the projects are active. Each project entry contains a textual description that describes the objectives, implementation, and outcomes of the project (see Supplementary Table S2 for examples). We track disbursements as reported in the CRS database and cannot trace subsequent downstream reallocations.

### Machine learning pipeline

We developed a machine learning pipeline to track ODA flows for public health using a state-of-the-art, open-source large language model (LLM). Our pipeline consists of three steps: In step 1, we translated and preprocessed the textual descriptions of ODA projects (translation and preprocessing). In step 2, we classified the ODA projects into 17 disease categories using a pre-trained LLM, which was guided by a tailored prompt (disease classification). Finally, in step 3, we validated our approach against (i) a conventional keyword-based classification approach and (ii) manual annotations of ODA projects (validation).

Step 1: Translation and preprocessing. Our dataset included all ODA projects between 2000–2022 (~4.7 million). We preprocessed the data by identifying the languages of the project descriptions using the langid library (version 1.1.6) and the langdetect library (version 1.0.9)^76,77^, and then translated all non-English descriptions into English using the Google Translate API^78^. We removed all invalid entries, such as projects with missing textual descriptions, missing funding amounts, or negative funding amounts. Finally, we concatenated the translated project title, long description, and short description into a single text field, which was then input into the machine learning pipeline. This preprocessing strategy is consistent with previous large-scale analyses of ODA project descriptions^70,79^. To compute per capita aid flows, we used population statistics from the World Bank^80^. The initial dataset contained 4,726,638 projects. After filtering out invalid entries (1,012,703 projects; 21.4%), the final cleaned dataset included 3,713,935 projects. The ODA project preprocessing and classification flow is illustrated in Supplementary Fig. S10.

Step 2: Disease classification. We classified ODA projects into 17 disease categories adapted from the Global Burden of Disease (GBD) cause hierarchy. Specifically, we selected all Level 2 causes from (i) CMNNDs and (ii) NCDs. We did not include injury-related categories and the residual Level 2 buckets ("other infectious diseases”, “other non-communicable diseases”) in the taxonomy because they have received comparatively little focus in health aid initiatives, and their heterogeneity limits interpretability and decision relevance in the context of development assistance for health. The resulting 17-category taxonomy is provided in Supplementary Table S1.

The classification method assigns multiple labels to a project when applicable, i.e., a project may belong to more than one disease category. For example, a project addressing both HIV and malnutrition is categorized under both “HIV/AIDS and STIs” and “nutritional deficiencies”. Since the CRS database does not report within-project allocations to specific disease areas, we distribute the funding equally among the applicable categories to prevent double-counting of the project funding, consistent with prior work^24,27,32,37^. In the aforementioned example, 50% of the project funding would be allocated to “HIV/AIDS and STIs” and 50% to “nutritional deficiencies”. Similarly, if an aid activity extends over multiple years, it is reported on a pro-rata basis per year, with the proportional aid disbursement attributed to the corresponding years. This approach ensures that each project’s total funding is accounted for only once, while accurately reflecting that development aid projects can pursue multiple objectives.

For the classification task, we used Llama-3.1-70B-Instruct-Turbo^81^ via the Together AI API^82^. This LLM is a state-of-the-art, open-source model, which, at the time of our analysis, ranked among the top-performing LLMs across various benchmarks^83^. We also experimented with other LLMs, including GPT-4, Claude Sonnet and Haiku, Mistral 7B, other Llama 3.1 variants (i.e., 70B-Instruct and 8B-Instruct). Eventually, our choice to select Llama-3.1-70B-Instruct-Turbo was based on three key considerations: (i) accuracy in structured classification tasks, (ii) scalability in terms of computational costs to enable a large-scale analysis of millions of aid projects, and (iii) consistent classification across varying project description lengths and complexities.

Using this LLM, we then classified the projects based on their textual descriptions into the 17 disease categories from above using a tailored prompt. The prompt design followed best practices in prompt engineering and prior research^84–86^, and included a task description, a list of possible labels, and the description of the ODA project to be classified. The model was instructed to adopt a conservative classification strategy to reduce false positives, using fallback categories ("Other” and “General Health”) where applicable. Additional implementation details, including the full prompt and model hyperparameters, are provided in Supplementary Material S3 and Supplementary Material S4, respectively. Reporting follows best practice^87^.

Step 3: Validation. To assess the accuracy and reliability of our ML approach, we employed a dual validation strategy. First, we benchmarked our approach against a conventional keyword-based classification method, which is interpretable, but unable to capture complex semantics as effectively as our LLM approach. The conventional keyword-based approach, inspired by refs. ^36,39^, relies on the predefined terms from our disease categories (see Supplementary Material S1). We then searched for exact matches (case-insensitive) within the title, long description, and short description of each project. Again, aid projects were assigned to one or more categories based on the matches. Projects without keyword match were classified as “Other”. Overall, the keyword-based approach shows large agreement with the results from our machine learning approach. However, there are a few exceptions, which we identified in a manual assessment as issues with the keyword-based search, thereby again highlighting the benefits of our machine learning approach.

Second, we manually annotated 2060 aid projects and compared the labels to the LLM-generated classifications (see Supplementary Material S1); however, this approach lacks the scalability of our machine learning pipeline. Again, we find strong agreement, with an overall accuracy of 0.91 (micro-averaged *F*_1_-score: 0.92; macro-averaged *F*_1_-score: 0.86), computed using scikit-learn (version 1.5), and robust performance across disease categories (both precision and recall  > 0.9 for the majority of disease categories).

### Analysis

For our analysis of how disease-specific development aid aligns with disease burden, we use disability-adjusted life years (DALYs) to measure disease burden^88,89^. A DALY combines years of life lost from premature death and years lived with disability. The DALY estimates originate from the Global Burden of Disease study, which systematically quantifies health loss due to diseases across 204 countries^90^. The GBD study provides annual disease burden estimates from 1990 to 2021 and uses standardized estimation approaches to ensure that disease burden metrics can be meaningfully compared across locations and time periods^91^. We then matched the aid and disease burden data at the country level (using ISO-3 country codes). Our ODA data spans 2000–2022; however, comparisons with disease burden are limited to the period 2000–2021, which was the latest available data from the Global Burden of Disease study at the time of our analysis.

To assess whether disease burden aligns with the volume of health-related ODA, we computed Spearman’s rank correlation coefficient using scipy (version 1.13). Low correlation may indicate disparities in aid allocation, potentially reflecting historical or donor-driven priorities, and may signal cases where disease burden is high but aid is relatively low, that is, aid–burden misalignment. All statistical tests are based on two-sided Spearman’s rank correlation tests. We selected Spearman’s method over Pearson’s to account for potential non-linear relationships and non-normal data distributions. Correlations were computed separately for each disease category and for each country income group. Income group classifications follow the OECD framework. Our analysis included least-developed countries (LDCs), lower-middle-income countries (LMICs), and upper-middle-income countries (UMICs), but excluded more advanced developing countries and territories (MADCTs) and other low-income countries due to limited data coverage. We further excluded microstates (e.g., Vatican City, Nauru, Tuvalu, etc.), as their small populations can result in disproportionately high per capita aid volumes, thereby distorting aid–burden misalignment estimates. We interpreted correlation coefficients according to established guidelines in statistical literature^92,93^.

To identify countries with aid–burden misalignment, we computed a disease-specific misalignment metric. This metric captures the relative position of each country within its income group by comparing disease burden and aid received. For each disease, countries are ranked based on two measures: (i) per capita aid received (aid rank percentile) and (ii) disease burden, measured as DALYs per 100,000 population (burden rank percentile). Aid-burden misalignment is calculated as the difference between these two percentiles. Positive values indicate countries with higher disease burden, but lower aid levels compared to their peers. This metric is designed to capture relative disparities, rather than absolute funding adequacy. It is a descriptive, rank-based indicator and should not be interpreted as a prescriptive criterion for funding allocation. For example, an aid–burden misalignment of +10 percentiles indicates that a country’s disease burden rank is 10 percentiles higher than its aid rank among countries in the same income group. Countries were ranked within their respective income groups to account for differences in economic capacity, which affect the ability to respond to health challenges. This reflects that an equivalent disease burden will create a relatively greater strain on countries with fewer domestic resources. This approach follows established practices in global health financing^94^, and aligns with the resource allocation frameworks of major international health financing institutions such as the Global Fund and Gavi, the Vaccine Alliance^47,95–98^. The rationale for taking into account economic capacity in health burden assessment is well-established^99–101^, with the Lancet Commission on Investing in Health and the WHO Commission on Macroeconomics and Health emphasizing the importance of economic context for both burden assessment and resource allocation^102,103^.

### Sensitivity analysis

To evaluate the sensitivity of our results to key modeling choices, we conducted several supplementary analyses. First, we studied a different metric, namely, disease incidence as an alternative to DALYs (Supplementary Fig. S3). Second, to assess the impact of the COVID-19 pandemic on the alignment between aid and burden, we separately analyzed the years 2020–2021 (Supplementary Figs. S4–S6). Third, to account for possible changes in funding priorities during the COVID-19 pandemic among donors, we excluded data from the years 2020 and 2021 for respiratory infections and TB from our analysis (Supplementary Figs. S7–S9). In all cases, we recomputed disease-specific per capita aid and Spearman correlations with burden using the same procedure as in the main analysis.

### Robustness checks

We conducted several supplementary analyses to assess the robustness of the disease-classification pipeline. Specifically, we re-ran the classification on a randomly sampled 1% of projects assigned to one of the named disease categories, using different random seeds and alternative LLMs, summarized agreement using Cohen’s kappa (*κ*) and the label consistency rate (LCR), and found consistently high run-to-run and inter-model agreement. In addition, we conducted targeted robustness analyses for specific failure modes, including (i) a chunk-based variant of the pipeline applied to projects with long descriptions, (ii) a negation-focused validation set of projects containing statements in which disease keywords occur in a negated context (e.g., “no tuberculosis”, “not HIV”, “excluding malaria”), (iii) a separate set of short, non-English, and semantically ambiguous project descriptions, where predictions from the pipeline were compared against additional manual labels, and (iv) stratification of projects according to the position of disease-related mentions within the text. Across these supplementary analyses, classification performance remained high in several targeted settings, supporting the overall robustness of the pipeline, although some alternative input-processing strategies showed lower performance than the main validation results (Supplementary Material S2). Finally, to quantify the impact of classification uncertainty on downstream estimates, we computed bootstrap-based 95% uncertainty intervals for disease-specific ODA shares (Supplementary Table S18).

### Reporting summary

Further information on research design is available in the Nature Portfolio Reporting Summary linked to this article.

## Supplementary information

Supplements

Description of Additional Supplementary Files

Supplementary data

Reporting Summary

Transparent Peer Review file

## Source data

Source data

## Data Availability

The processed data generated in this study have been deposited in the Open Science Framework (OSF) repository at https://osf.io/ce3q2/. Source data are provided with this paper. The raw aid data used in this study are available from the OECD Creditor Reporting System at https://data-explorer.oecd.org/. The disease burden data used in this study are available from the Global Burden of Disease study^40,41^. Population data used to compute per capita aid are available from the World Bank at https://data.worldbank.org/indicator/SP.POP.TOTL. Source data are provided with this paper.

## Code availability

The code used to reproduce the analyses, tables, and figures reported in this study is available on GitHub at https://github.com/forsterkerstin/tracking-health-aid-disparitiesand has been archived on Zenodo^104^.
