# Supplementary material for: Tracking funding disparities in global health aid with machine learning
Source: Nat Commun. 2026 Aug 18;17:8569. doi: 10.1038/s41467-026-76542-z (PMC13486753; doi:10.1038/s41467-026-76542-z)
Supplement: Supplementary file 1 [file 41467_2026_76542_MOESM1_ESM.pdf]

Supplements

Supplementary Tables

S1 Classification of causes of disease burden 4

S2 Example ODA projects with metadata and project description 5

S3 Keywords used as search terms for HIV/AIDS and sexually transmitted infections . 28

S4 Frequency of labels in the manually-annotated validation dataset 35

S5 Overall classification performance based on manually-labeled validation data . . . 36

S6 Classification performance by disease categories for the machine learning approach 37

S7 Classification performance by disease categories for the keyword-based approach . 38

S8 Distribution of disease categories in the 1% subset and in the full corpus. 41

S9 Overall classification performance using chunk-based classification. 51

S10 Classification performance by disease category using chunk-based classification. . . 52

S11 Overall classification performance on short, ambiguous, and non-English project descriptions. 52

S12 Classification performance on short, ambiguous, and non-English project descriptions by disease category. 53

S13 Overall classification performance on project descriptions including negation. . . . 54

S14 Classification performance by disease category on project descriptions including negation using the original prompt. 55

S15 Classification performance by disease category on project descriptions including negation using the negation-aware prompt. 56

S16 Classification performance by position of disease mention. 56

S17 Share and cumulative share of global development funding by donor from 2000 to 2022. . . . 60
S18 Disease-specific downstream ODA allocation estimates from the main classification. 61

Supplementary Figures

S1 Funding disparities at the country level (for NCDs) 8
S2 Geographic distribution of aid–burden misalignment (for NCDs) 10
S3 Correlation between disease-specific aid and incidence across income groups . . . 11
S4 Correlation between disease-specific aid and burden across income groups (for 2020–2021) 12
S5 Funding disparities at the country level (for 2020–2021) 16
S6 Geographic distribution of aid–burden misalignment (for 2020–2021) 18
S7 Correlation between disease-specific aid and burden across income groups (as a sensitivity analysis discounting for the surge in aid earmarked to COVID-19) . . . 19
S8 Funding disparities at the country level (without the years 2020 and 2021 for respiratory infections and TB) 23
S9 Geographic distribution of aid–burden misalignment without the years 2020 and 2021 for respiratory infections and TB 25
S10 ODA project preprocessing and classification flow. 26
S11 Comparison between our machine learning pipeline based on LLMs and keyword-based classification 29
S12 Comparison of project description lengths in the full and validation datasets 32
S13 Sensitivity analysis of classification performance 33
S14 Comparison between our manual annotators 34
S15 Comparison between our machine learning pipeline and the manually-annotated dataset 39

S16 Comparison between classifications from different random seeds, weighted by funding. 42
S17 Comparison between classifications from different random seeds, weighted by funding. 43
S18 Comparison between GPT-4.1-mini-2025-04-14 classifications and primary model classifications, weighted by funding. 44
S19 Comparison between GPT-4.1-mini-2025-04-14 classifications and primary model classifications. 45
S20 Comparison between DeepSeek-V3.1 classifications and primary model classifications, weighted by funding. 46
S21 Comparison between DeepSeek-V3.1 classifications and primary model classifications. 47
S22 Prompt design for the chunk-based classification. 51
S23 Prompt design for the negation-aware classification. 54
S24 Prompt design 58

Supplementary Materials

S1 Comparison against validation datasets 27
S2 Robustness checks 40
S3 Prompt design 57
S4 Hyperparameters 59
S5 Keyword list 62

Supplementary Tables

Communicable, maternal, neonatal, and nutritional diseases (CMNNDs)
HIV/AIDS and sexually transmitted infections (STIs)
Respiratory infections and tuberculosis (TB)
Nutritional deficiencies
Neglected tropical diseases (NTDs) and malaria
Maternal and neonatal disorders
Enteric infections
**Non-communicable diseases (NCDs)**
Mental disorders
Neoplasms
Substance use disorders
Sense organ diseases
Chronic respiratory diseases
Cardiovascular diseases
Diabetes and kidney diseases
Musculoskeletal disorders
Neurological disorders
Digestive diseases
Skin and subcutaneous diseases

Table S1: **Classification of causes of disease burden**. The categorization is based on the causes of disease burden in the Global Burden of Disease study [1, 2]. Specifically, we selected all Level 2 causes from (i) CMNNDs and (ii) NCDs. We did not include injury-related categories and the residual Level 2 buckets ("other infectious diseases", "other non-communicable diseases") in the taxonomy because they have received comparatively little focus in health aid initiatives and their heterogeneity limits interpretability and decision relevance in the context of development assistance for health.

**Parameter**	**Details**
Year	2017
Donor	UNDP
Recipient country	Angola
Income group	LDCs
Project title	Reducing the Burden of HIV/AIDS
Project description	*Strengthened the institutional capacity of the national HIV&AIDS programme, National health information system, epidemiological surveillance and expansion of counseling and testing centres*
Year	2021
Donor	Global Alliance for Vaccines and Immunization
Recipient country	Uganda
Income group	LDCs
Project title	New vaccine support (NVS)
Project description	*Vaccine against rotavirus, which causes diarrhoea.*
Year	2020
Donor	France
Recipient	Niger
Income group	LDCs
Project title	P209 - Fonds Muskoka - Niger - ONU femmes
Project description	*The French Muskoka Fund (FFM) has been operating since 2011 in West and Central Africa to accelerate the reduction of maternal and infant mortality and improve reproductive, sexual, Maternal, Neonatal, Child, Adolescent and Nutrition (RMNCIA-N) health outcomes.*
Year	2021
Donor	Spain
Recipient country	Sudan
Income group	LDCs
Project title	Vaccine support for covid-19
Project description	*Donation via COVAX of 2.325.600 doses of Janssen COVID-19 vaccines to Sudan.*

Table S2: **Example ODA projects with metadata and project description.**

Supplementary Figures

Funding disparities at the country level (for NCDs)

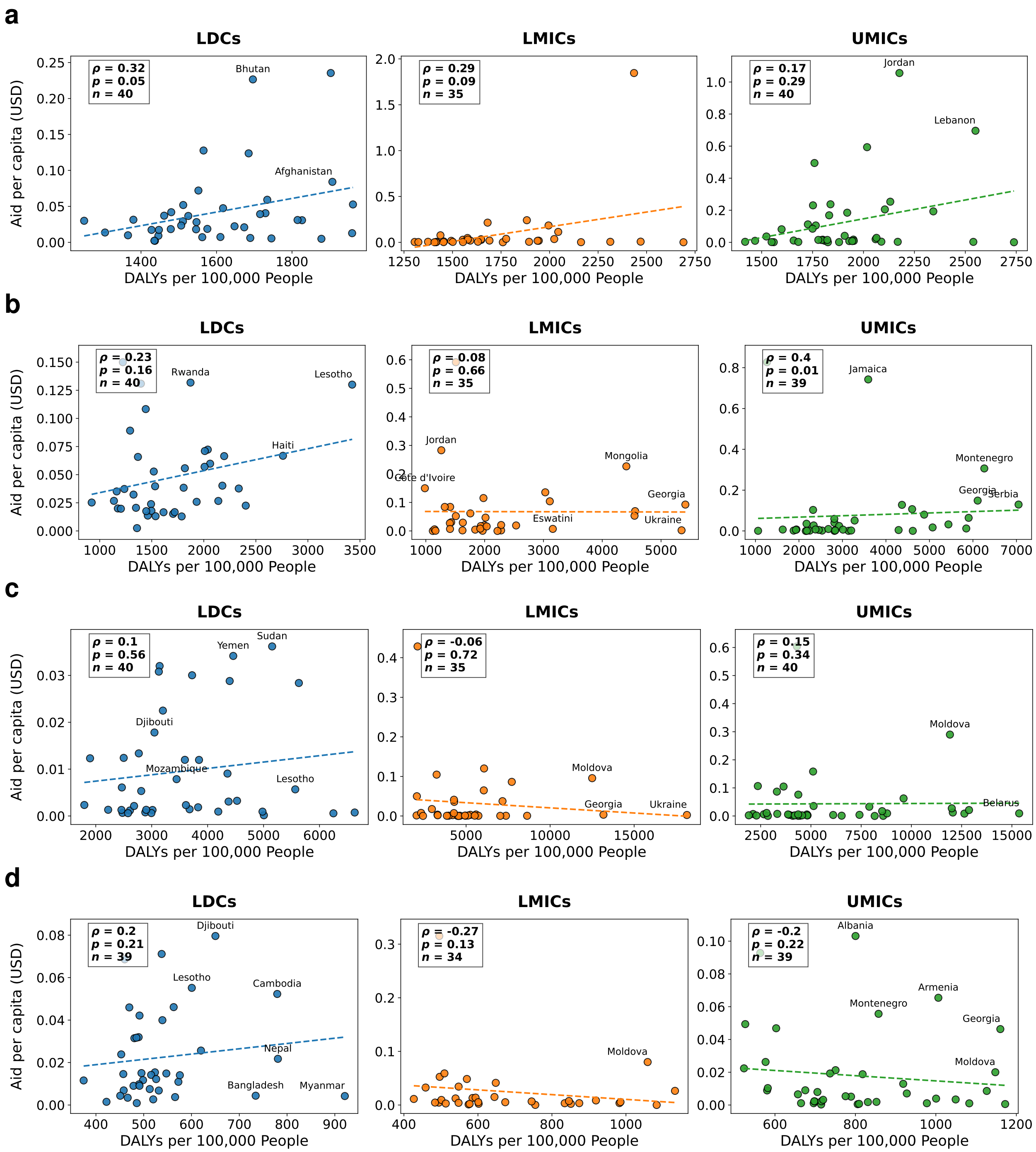

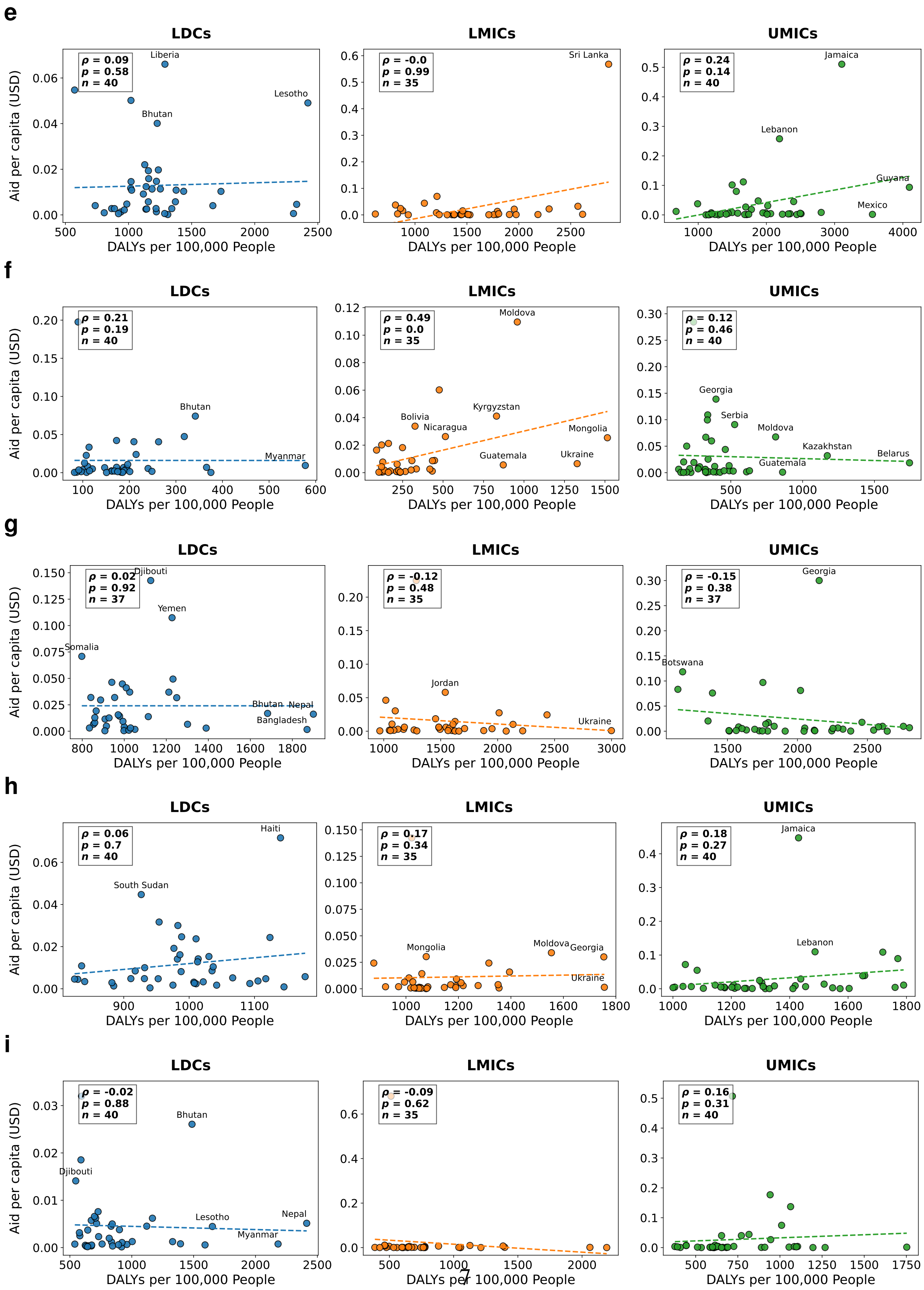
e
LDCs
LMICs
UMICs
ρ = 0.09
p = 0.58
n = 40
Liberia
Lesotho
Bhutan
ρ = -0.0
p = 0.99
n = 35
Sri Lanka
ρ = 0.24
p = 0.14
n = 40
Jamaica
Lebanon
Guyana
Mexico
f
LDCs
LMICs
UMICs
ρ = 0.21
p = 0.19
n = 40
Bhutan
Myanmar
ρ = 0.49
p = 0.0
n = 35
Moldova
Kyrgyzstan
Bolivia
Nicaragua
Mongolia
Guatemala
Ukraine
ρ = 0.12
p = 0.46
n = 40
Georgia
Serbia
Moldova
Kazakhstan
Belarus
Guatemala
g
LDCs
LMICs
UMICs
ρ = 0.02
p = 0.92
n = 37
Djibouti
Yemen
Somalia
Bhutan
Nepal
Bangladesh
ρ = -0.12
p = 0.48
n = 35
Jordan
Ukraine
ρ = -0.15
p = 0.38
n = 37
Georgia
Botswana
h
LDCs
LMICs
UMICs
ρ = 0.06
p = 0.7
n = 40
Haiti
South Sudan
ρ = 0.17
p = 0.34
n = 35
Mongolia
Moldova
Georgia
Ukraine
ρ = 0.18
p = 0.27
n = 40
Jamaica
Lebanon
i
LDCs
LMICs
UMICs
ρ = -0.02
p = 0.88
n = 40
Bhutan
Djibouti
Lesotho
Nepal
Myanmar
ρ = -0.09
p = 0.62
n = 35
ρ = 0.16
p = 0.31
n = 40
Aid per capita (USD)
DALYs per 100,000 People

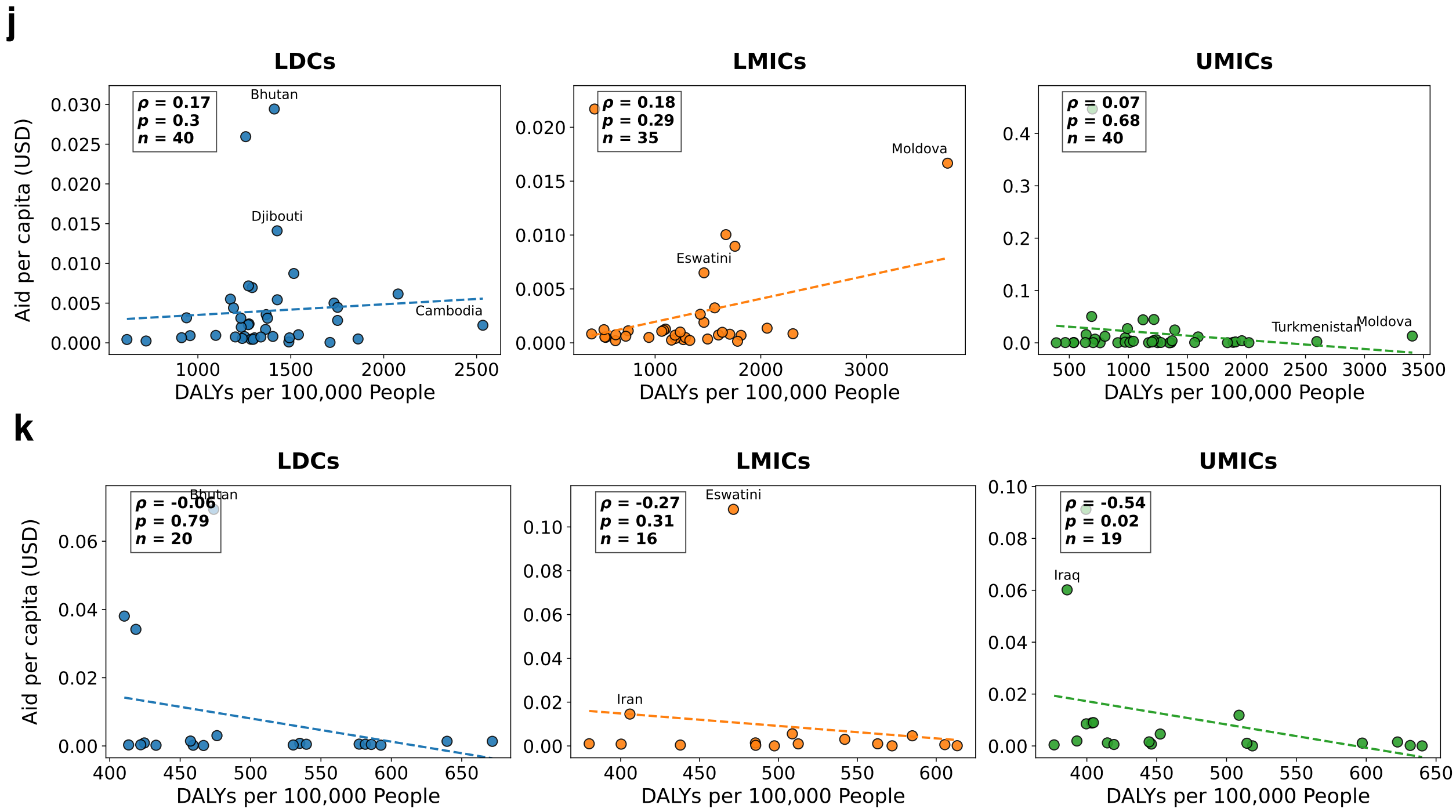

Fig. S1. Funding disparities at the country level (for NCDs). The plots compare the disease burden (x-axis, measured by DALYs per 100,000 population, accumulated for 2000–2021) against the aid per capita (y-axis, accumulated for 2000–2021). Each point represents a single country, which allows to identify countries with high disease burden but comparatively low aid and thus to reveal aid–burden misalignment. For each disease, countries/plots are grouped by country income groups: least developed countries (LDCs), lower-middle-income countries (LMICs) and upper-middle-income countries (UMICs). The figure shows: **a**, mental disorders; **b**, neoplasms; **c**, cardiovascular diseases; **d**, sense organ diseases; **e**, diabetes and kidney diseases; **f**, substance use disorders; **g**, musculoskeletal disorders; **h**, neurological disorders; **i**, chronic respiratory diseases; **j**, digestive diseases; and **k**, skin and subcutaneous diseases. Each plot reports Spearman's rank correlation coefficient (ρ), the corresponding p-value from a two-sided Spearman rank test, and the number of countries included in the analysis (n). The dashed line is the fitted trend line from an ordinary least squares regression. Abbreviations: DALYs, disability-adjusted life years.

Geographic distribution of aid–burden misalignment (for NCDs)

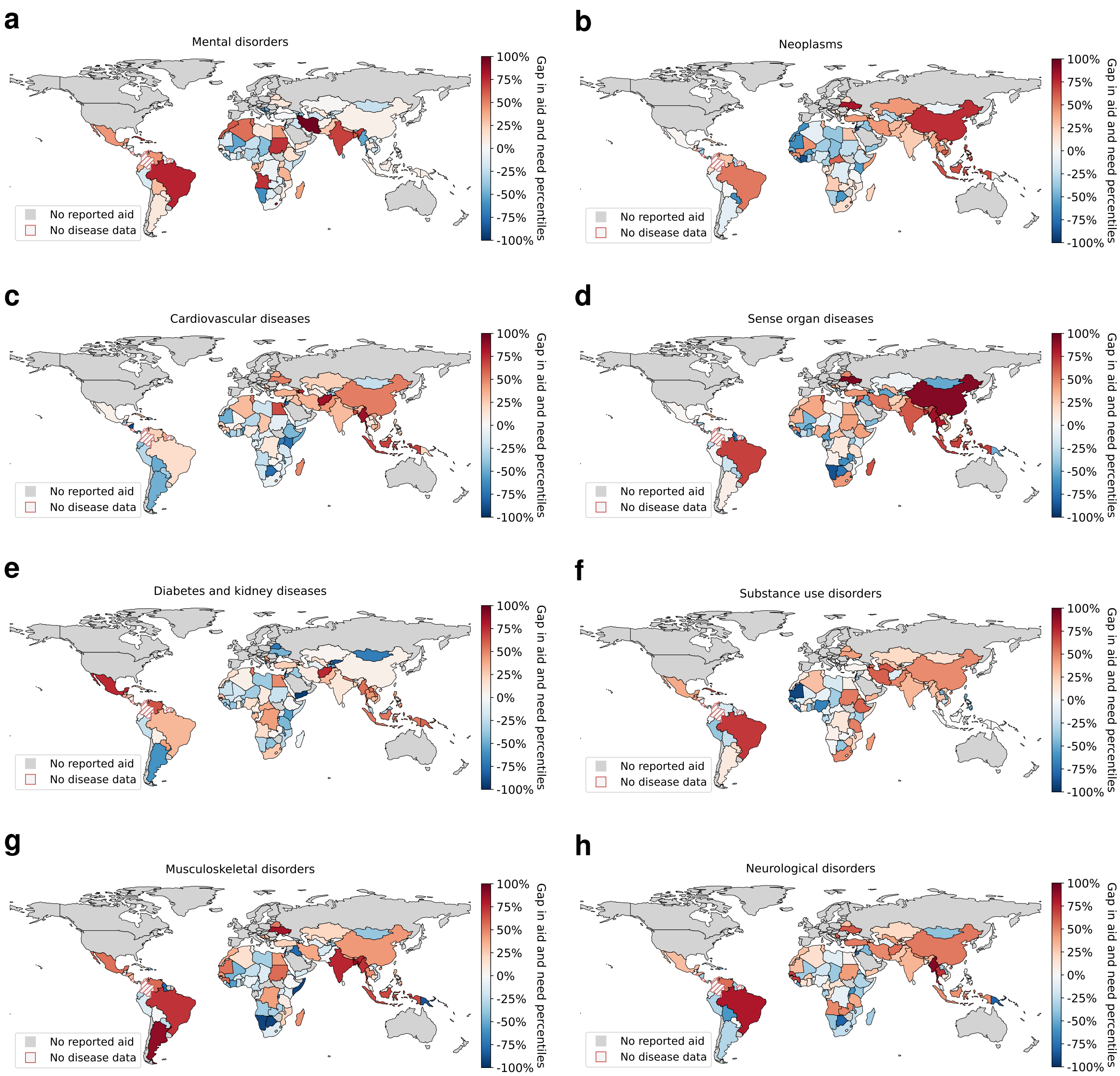

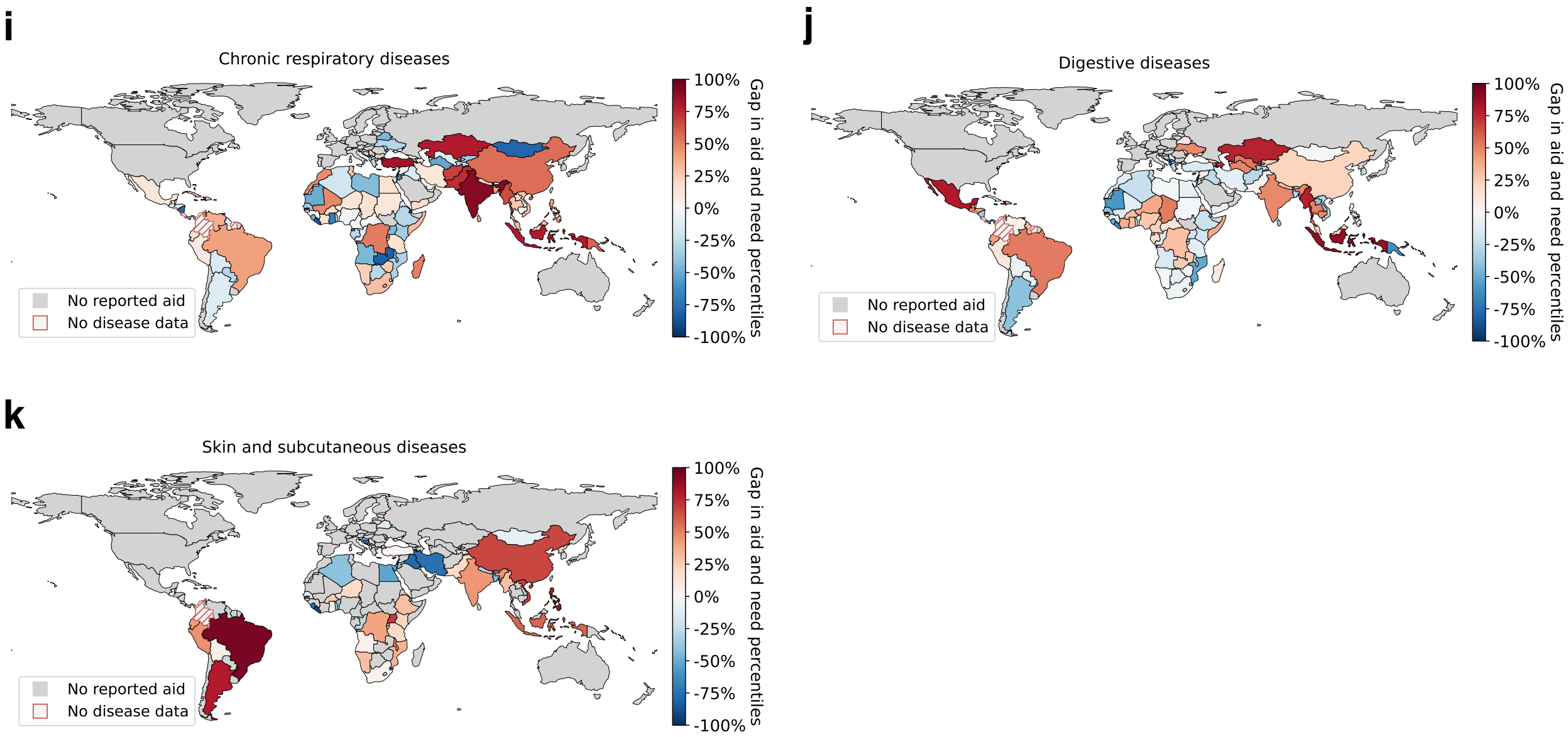

Fig. S2. Geographic distribution of aid–burden misalignment (for NCDs). The maps show the relative aid–burden misalignment by disease. Here, we refer to "aid–burden misalignment" as the difference between a country's percentile in disease burden (DALYs per 100,000 population) and its percentile in per capita aid, calculated within income groups. In other words, an aid–burden misalignment of 10 percentiles means that the country's disease burden is 10 percentiles higher than its aid level. Put simply, assuming 100 peer countries, this country would need to climb 10 places in the aid rank to have a similar rank in terms of aid and burden. The figure shows: **a**, mental disorders; **b**, neoplasms; **c**, cardiovascular diseases; **d**, sense organ diseases; **e**, diabetes and kidney diseases; **f**, substance use disorders; **g**, musculoskeletal disorders; **h**, neurological disorders; **i**, chronic respiratory diseases; **j**, digestive diseases; and **k**, skin and subcutaneous diseases. Red color denotes aid–burden misalignment, where a country's aid rank is lower than its burden rank. Blue color denotes that a country's aid rank is higher than the burden rank. Gray indicates no reported aid; red striped areas indicate no disease data. Country boundaries are from Natural Earth, 1:110m Admin 0 Countries (public domain). Abbreviations: DALYs, disability-adjusted life years.

Alignment using disease incidence (instead of DALYs)

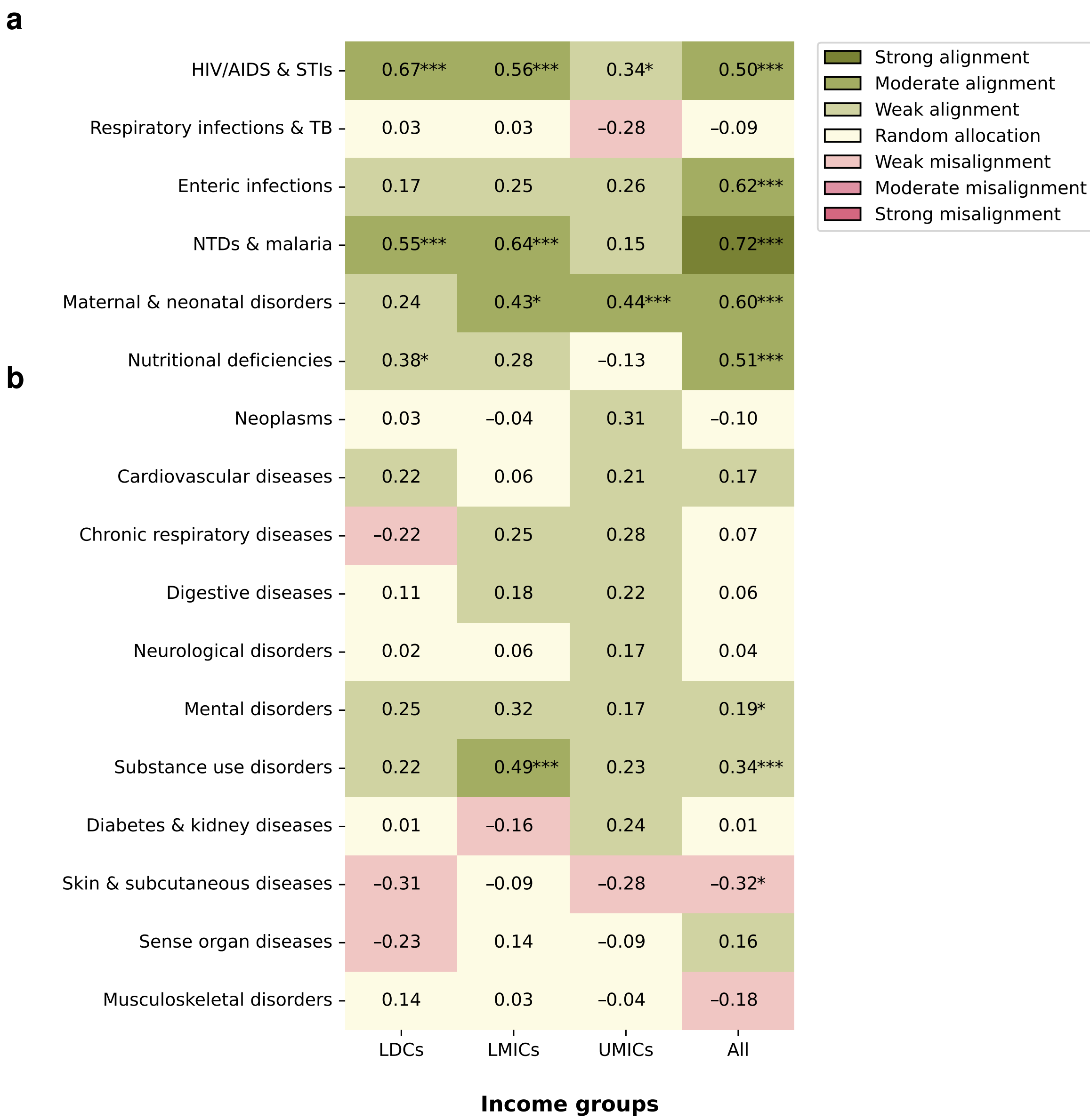

Fig. S3. Correlation between disease-specific aid and incidence across income groups. Shown are Spearman's rank correlation coefficients (ρ) between per capita aid (accumulated over the time period 2000–2021) and disease incidence (as reported in the Global Burden of Disease study) across country income groups: least developed countries (LDCs), lower-middle-income countries (LMICs), upper-middle-income countries (UMICs), and all countries combined. **a**, The top six rows report CMNNDs, which generally show positive correlations across most income groups. **b**, In contrast, NCDs (bottom eleven rows) exhibit more heterogeneity in the correlations. CMNNDs (top six rows) predominantly show positive correlations across most income groups, while NCDs (bottom eleven rows) display more varied patterns. Green indicates positive correlations and red indicates negative correlations. Asterisks denote statistically significant correlations based on two-sided Spearman rank tests, where p denotes the corresponding p-value (* $p < 0.05$; ** $p < 0.01$; *** $p < 0.001$). Legend interpretation: -1 to -0.7: strong misalignment of health aid with disease burden; -0.7 to -0.3: moderate misalignment; -0.3 to -0.1: weak misalignment, -0.1 to 0.1: indicative of random allocation; 0.1 to 0.3: weak alignment, 0.3 to 0.7, moderate alignment; and 0.7 to 1.0 strong alignment. Abbreviations: CMNNDs, communicable, maternal, neonatal, and nutritional diseases; NCDs, non-communicable diseases; DALYs, disability-adjusted life years; STI, sexually transmitted infections; TB, tuberculosis; NTDs, neglected tropical diseases.

Sensitivity analysis for 2020 to 2021

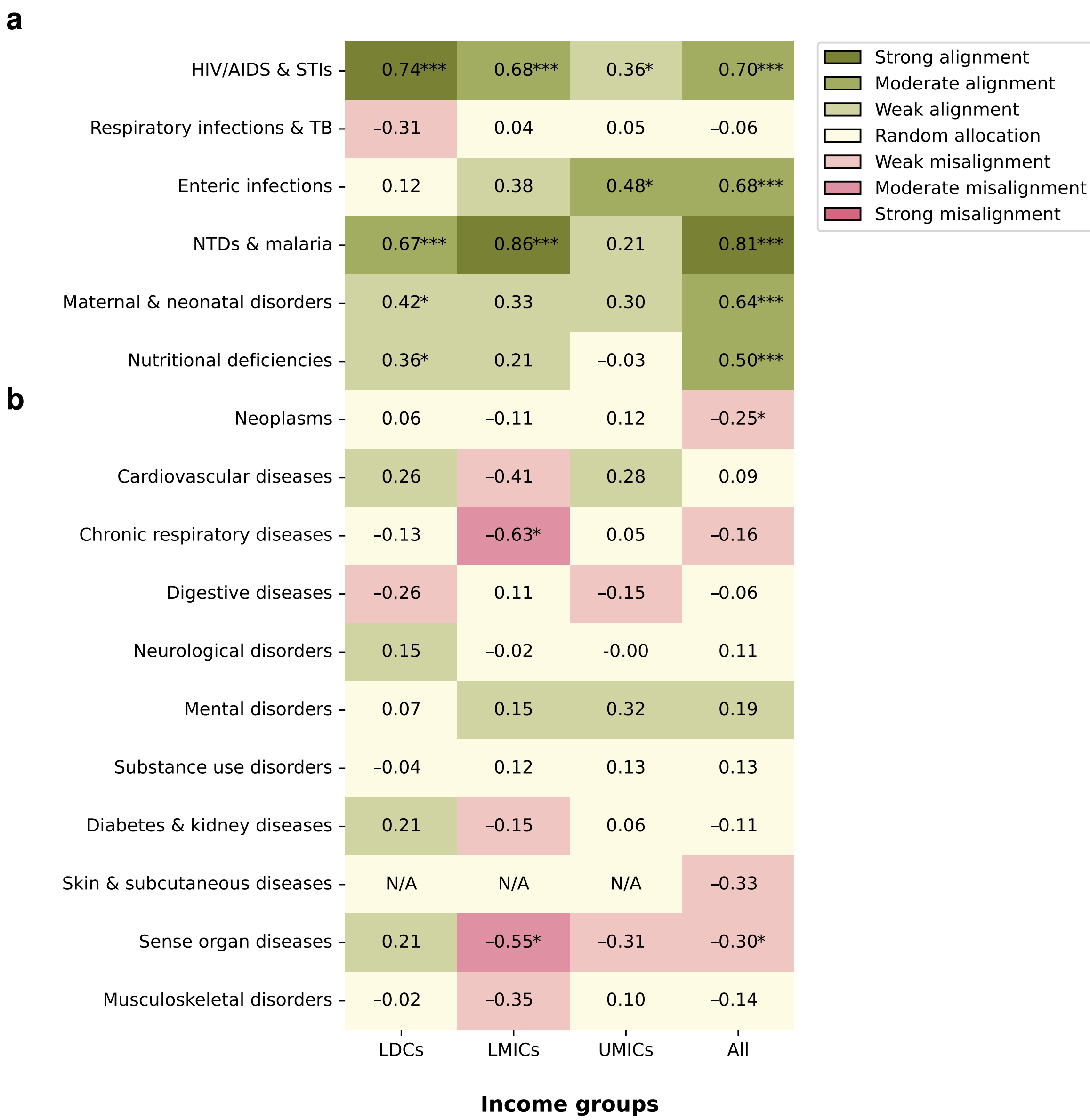

Fig. S4. Correlation between disease-specific aid and burden across income groups (for 2020–2021). Shown are Spearman's rank correlation coefficients between per capita aid (accumulated over the time period 2020–2021) and disease burden across country income groups: least developed countries (LDCs), lower-middle-income countries (LMICs), upper-middle-income countries (UMICs), and all countries combined. **a**, The top six rows report CMNNDs. **b**, The bottom 11 rows show NCDs. Green indicates positive and red negative correlations. Asterisks denote statistically significant correlations based on a two-sided Spearman rank test (* $p < 0.05$; ** $p < 0.01$; *** $p < 0.001$). Legend interpretation: -1 to -0.7: strong misalignment of health aid with disease burden; -0.7 to -0.3: moderate misalignment; -0.3 to -0.1: weak misalignment, -0.1 to 0.1: indicative of random allocation; 0.1 to 0.3: weak alignment, 0.3 to 0.7, moderate alignment; and 0.7 to 1.0 strong alignment. Abbreviations: CMNNDs, communicable, maternal, neonatal, and nutritional diseases; NCDs, non-communicable diseases; DALYs, disability-adjusted life years; STI, sexually transmitted infections; TB, tuberculosis; NTDs, neglected tropical diseases.

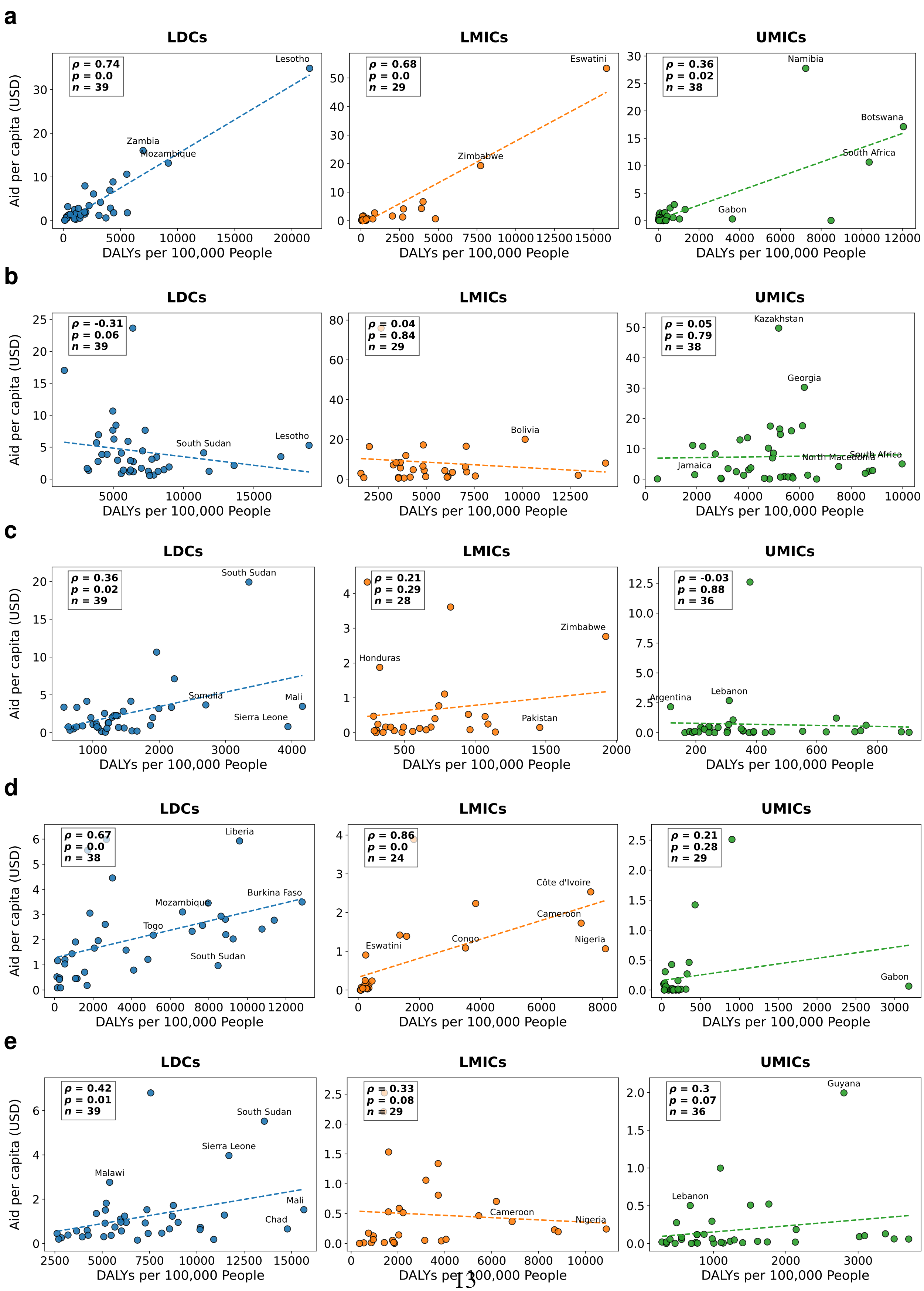
a
LDCs
LMICs
UMICs
ρ = 0.74
p = 0.0
n = 39
Lesotho
Zambia
Mozambique
ρ = 0.68
p = 0.0
n = 29
Eswatini
Zimbabwe
ρ = 0.36
p = 0.02
n = 38
Namibia
Botswana
South Africa
Gabon
b
LDCs
LMICs
UMICs
ρ = -0.31
p = 0.06
n = 39
South Sudan
Lesotho
ρ = 0.04
p = 0.84
n = 29
Bolivia
ρ = 0.05
p = 0.79
n = 38
Kazakhstan
Georgia
Jamaica
North Macedonia
South Africa
c
LDCs
LMICs
UMICs
ρ = 0.36
p = 0.02
n = 39
South Sudan
Somalia
Mali
Sierra Leone
ρ = 0.21
p = 0.29
n = 28
Zimbabwe
Honduras
Pakistan
ρ = -0.03
p = 0.88
n = 36
Argentina
Lebanon
d
LDCs
LMICs
UMICs
ρ = 0.67
p = 0.0
n = 38
Liberia
Burkina Faso
Mozambique
Togo
South Sudan
ρ = 0.86
p = 0.0
n = 24
Côte d'Ivoire
Cameroon
Nigeria
Congo
Eswatini
ρ = 0.21
p = 0.28
n = 29
Gabon
e
LDCs
LMICs
UMICs
ρ = 0.42
p = 0.01
n = 39
South Sudan
Sierra Leone
Malawi
Mali
Chad
ρ = 0.33
p = 0.08
n = 29
Cameroon
Nigeria
ρ = 0.3
p = 0.07
n = 36
Guyana
Lebanon
Aid per capita (USD)
DALYs per 100,000 People

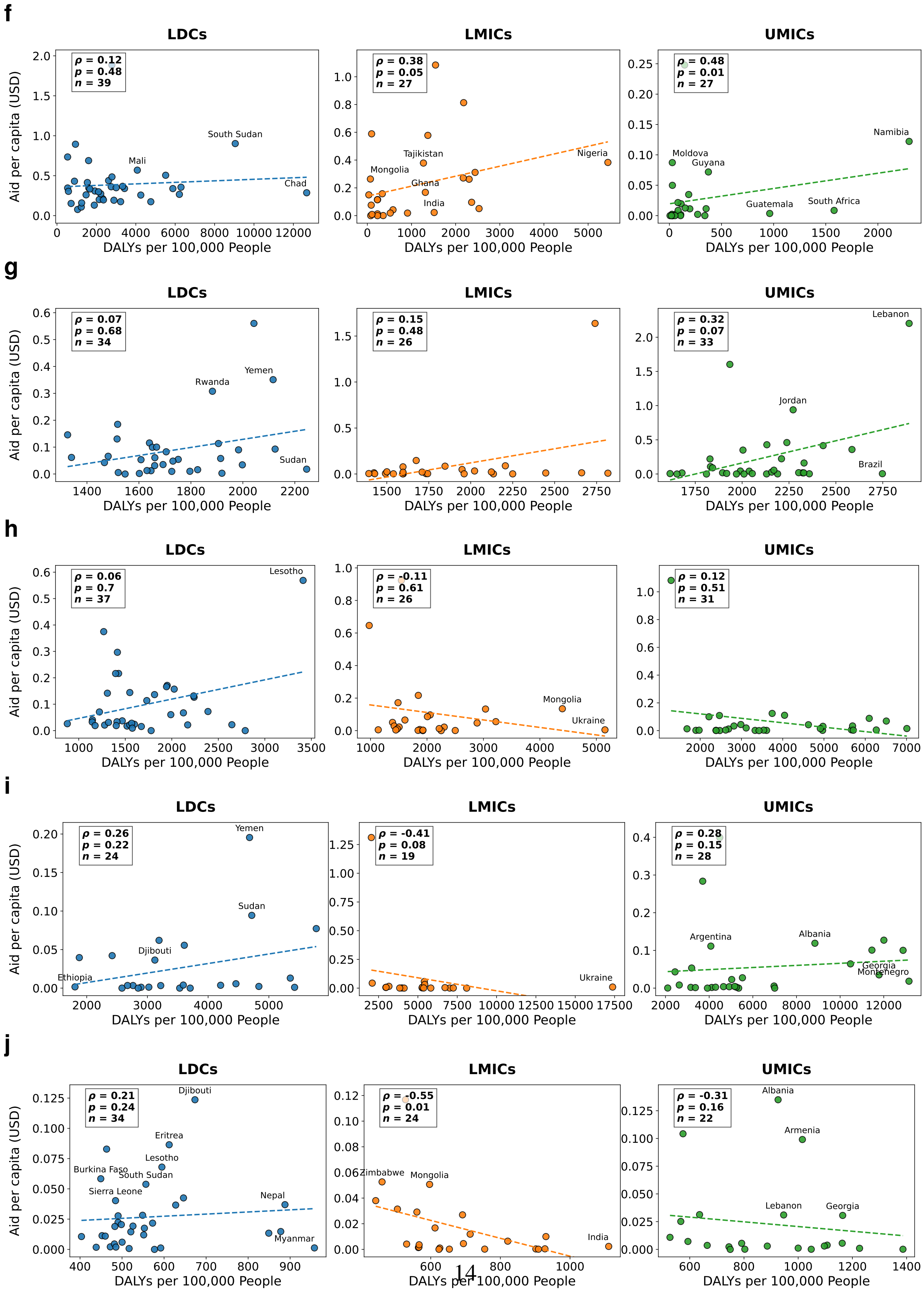
f
LDCs
ρ = 0.12
p = 0.48
n = 39
South Sudan
Mali
Chad
LMICs
ρ = 0.38
p = 0.05
n = 27
Tajikistan
Mongolia
Ghana
India
Nigeria
UMICs
ρ = 0.48
p = 0.01
n = 27
Moldova
Guyana
Namibia
Guatemala
South Africa
g
LDCs
ρ = 0.07
p = 0.68
n = 34
Rwanda
Yemen
Sudan
LMICs
ρ = 0.15
p = 0.48
n = 26
UMICs
ρ = 0.32
p = 0.07
n = 33
Lebanon
Jordan
Brazil
h
LDCs
ρ = 0.06
p = 0.7
n = 37
Lesotho
LMICs
ρ = -0.11
p = 0.61
n = 26
Mongolia
Ukraine
UMICs
ρ = 0.12
p = 0.51
n = 31
i
LDCs
ρ = 0.26
p = 0.22
n = 24
Yemen
Sudan
Djibouti
Ethiopia
LMICs
ρ = -0.41
p = 0.08
n = 19
Ukraine
UMICs
ρ = 0.28
p = 0.15
n = 28
Argentina
Albania
Georgia
Montenegro
j
LDCs
ρ = 0.21
p = 0.24
n = 34
Djibouti
Eritrea
Lesotho
Burkina Faso
South Sudan
Sierra Leone
Nepal
Myanmar
LMICs
ρ = -0.55
p = 0.01
n = 24
Zimbabwe
Mongolia
India
UMICs
ρ = -0.31
p = 0.16
n = 22
Albania
Armenia
Lebanon
Georgia
Aid per capita (USD)
DALYs per 100,000 People

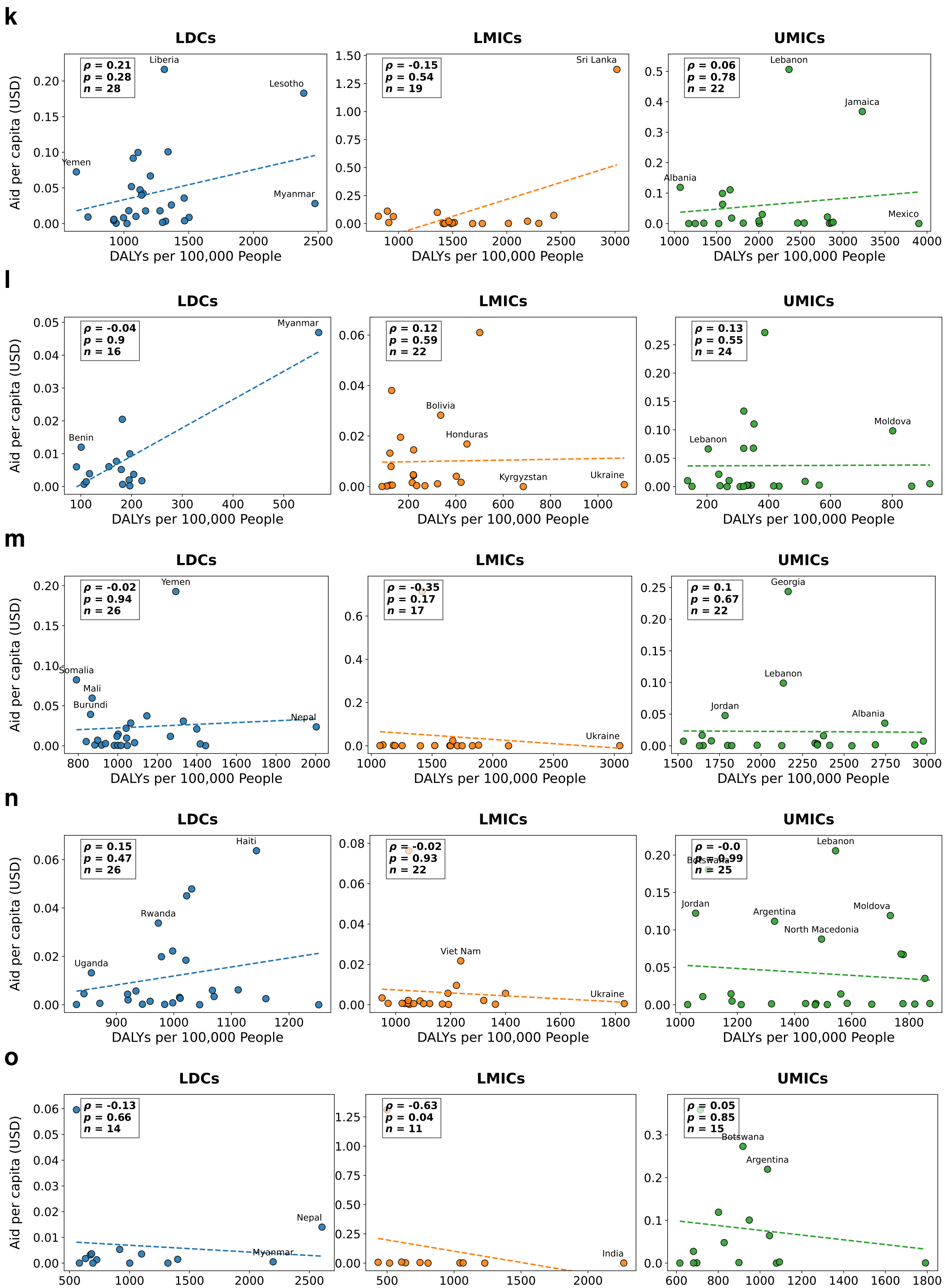
k
LDCs
ρ = 0.21
p = 0.28
n = 28
Liberia
Lesotho
Yemen
Myanmar
LMICs
ρ = -0.15
p = 0.54
n = 19
Sri Lanka
UMICs
ρ = 0.06
p = 0.78
n = 22
Lebanon
Jamaica
Albania
Mexico
l
LDCs
ρ = -0.04
p = 0.9
n = 16
Myanmar
Benin
LMICs
ρ = 0.12
p = 0.59
n = 22
Bolivia
Honduras
Kyrgyzstan
Ukraine
UMICs
ρ = 0.13
p = 0.55
n = 24
Lebanon
Moldova
m
LDCs
ρ = -0.02
p = 0.94
n = 26
Yemen
Somalia
Mali
Burundi
Nepal
LMICs
ρ = -0.35
p = 0.17
n = 17
Ukraine
UMICs
ρ = 0.1
p = 0.67
n = 22
Georgia
Lebanon
Jordan
Albania
n
LDCs
ρ = 0.15
p = 0.47
n = 26
Haiti
Rwanda
Uganda
LMICs
ρ = -0.02
p = 0.93
n = 22
Viet Nam
Ukraine
UMICs
ρ = -0.0
p = 0.99
n = 25
Lebanon
Botswana
Jordan
Argentina
Moldova
North Macedonia
o
LDCs
ρ = -0.13
p = 0.66
n = 14
Nepal
Myanmar
LMICs
ρ = -0.63
p = 0.04
n = 11
India
UMICs
ρ = 0.05
p = 0.85
n = 15
Botswana
Argentina
Aid per capita (USD)
DALYs per 100,000 People

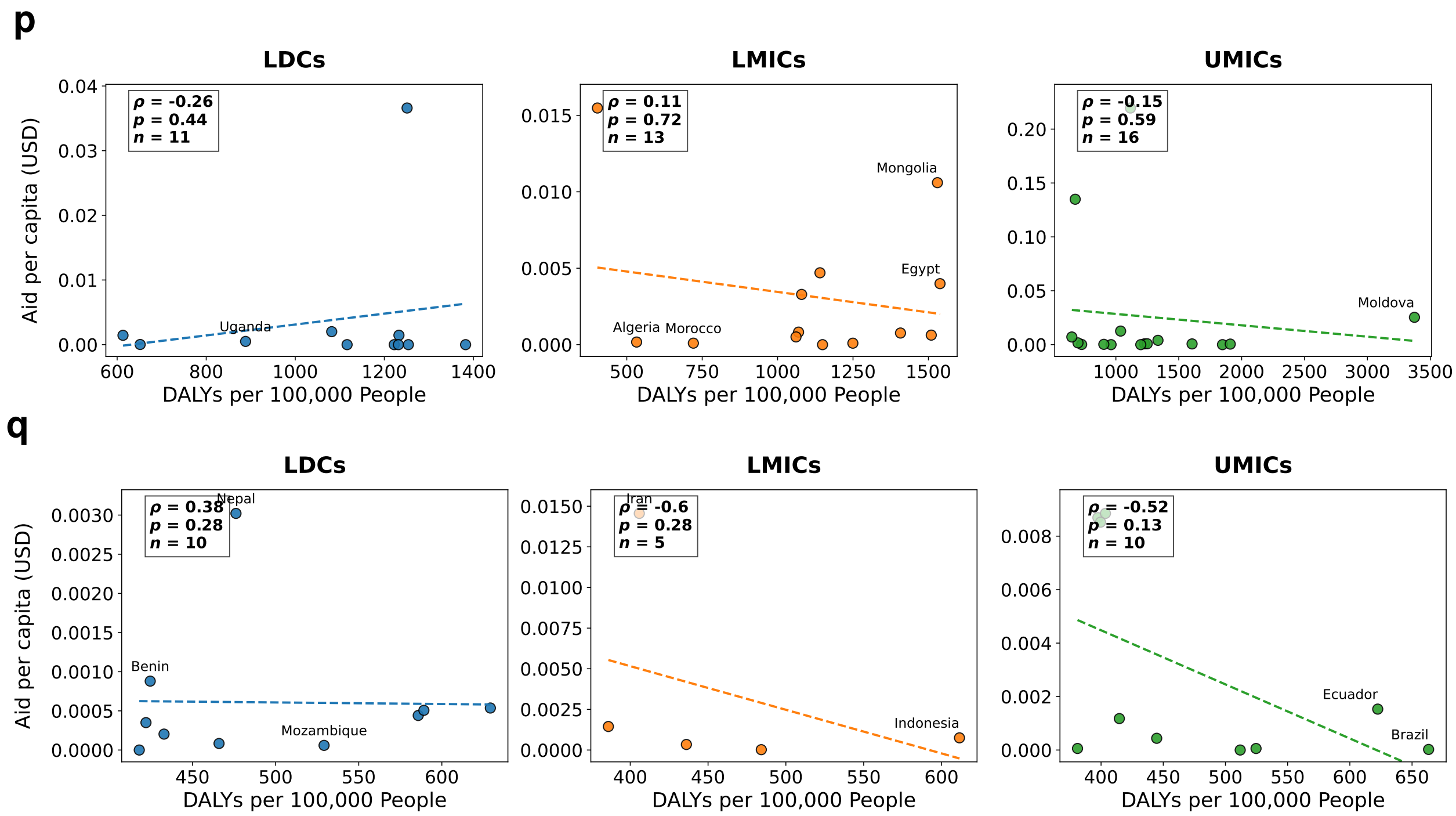

Fig. S5. Funding disparities at the country level (for 2020–2021). The figure is analogous to Figure 8 in the main paper, except that aid and disease burden are accumulated over 2020–2021. The figure shows: **a**, HIV/AIDS and sexually transmitted infections; **b**, respiratory infections and tuberculosis; **c**, nutritional deficiencies; **d**, neglected tropical diseases and malaria; **e**, maternal and neonatal disorders; **f**, enteric infections; **g**, mental disorders; **h**, neoplasms; **i**, cardiovascular diseases; **j**, sense organ diseases; **k**, diabetes and kidney diseases; **l**, substance use disorders; **m**, musculoskeletal disorders; **n**, neurological disorders; **o**, chronic respiratory diseases; **p**, digestive diseases; and **q**, skin and subcutaneous diseases. Each plot reports Spearman's rank correlation coefficient (ρ), the corresponding p-value from a two-sided Spearman rank test, and the number of countries included in the analysis (n). The dashed line denotes the fitted trend line from an ordinary least squares regression. Abbreviations: DALYs, disability-adjusted life years.

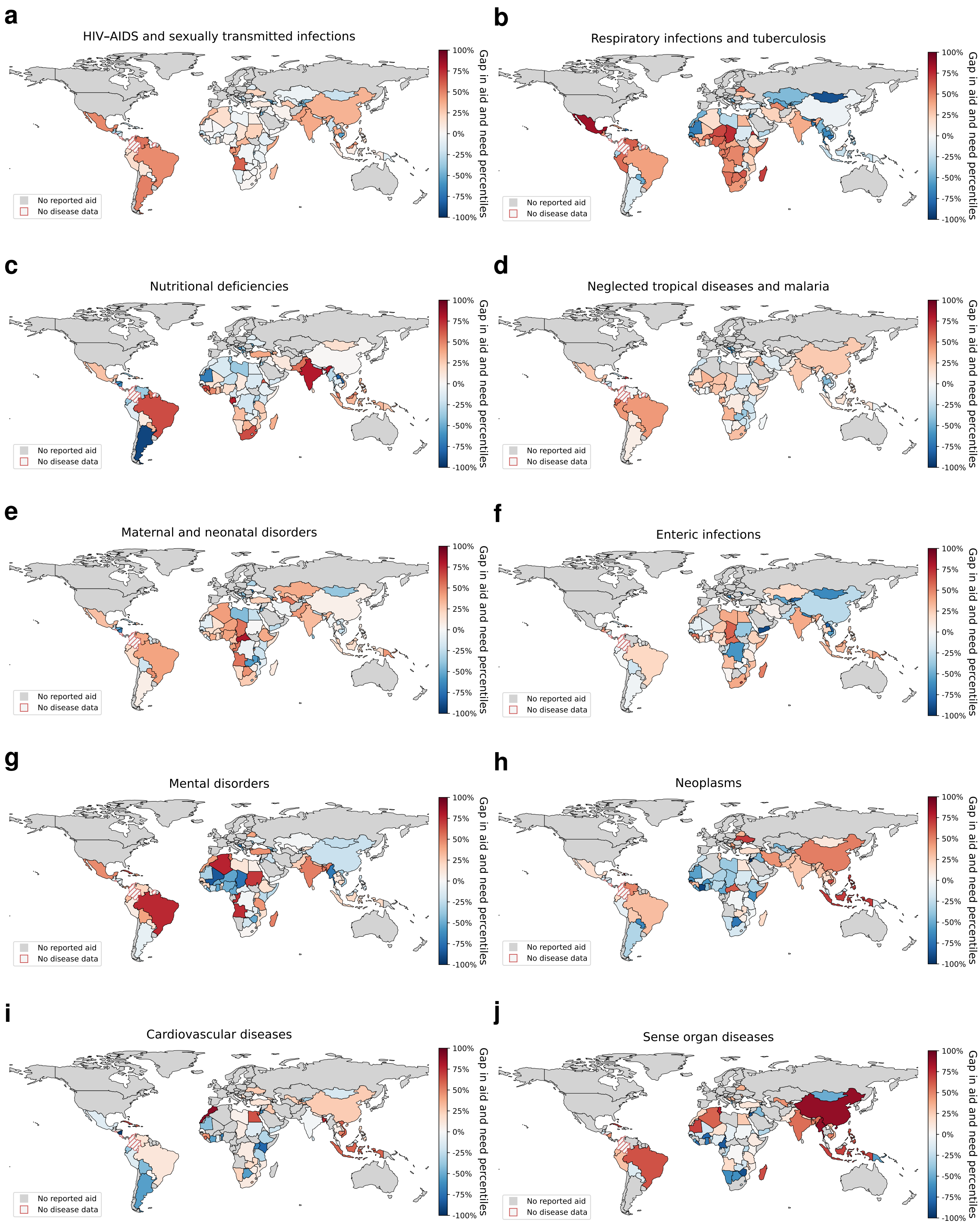
a
HIV–AIDS and sexually transmitted infections
b
Respiratory infections and tuberculosis
c
Nutritional deficiencies
d
Neglected tropical diseases and malaria
e
Maternal and neonatal disorders
f
Enteric infections
g
Mental disorders
h
Neoplasms
i
Cardiovascular diseases
j
Sense organ diseases
No reported aid
No disease data
Gap in aid and need percentiles
100%
75%
50%
25%
0%
-25%
-50%
-75%
-100%

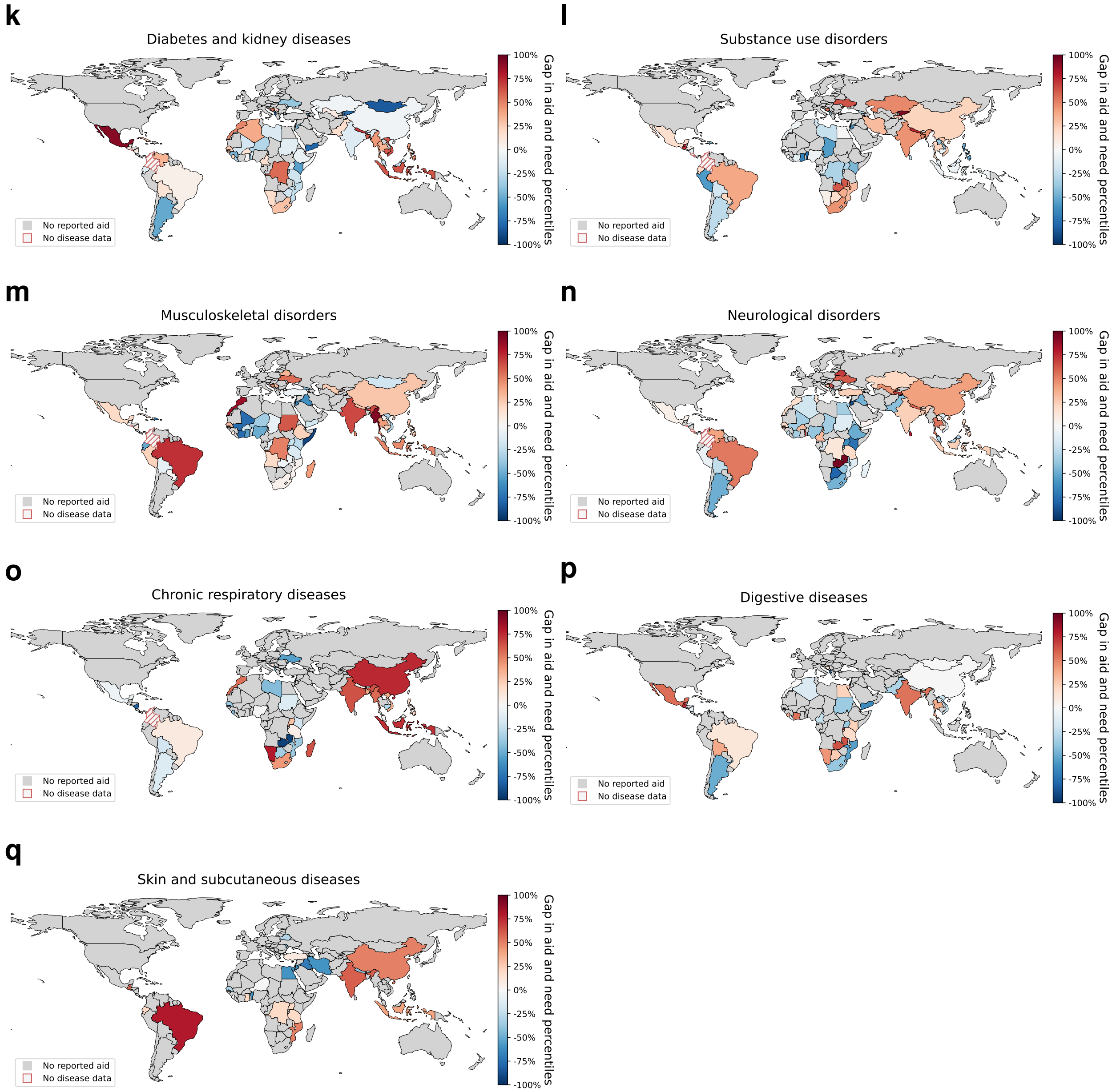

Fig. S6. Geographic distribution of aid–burden misalignment (for 2020–2021). The maps show the relative aid–burden misalignment by disease. Here, we refer to "aid–burden misalignment" as the difference between a country's percentile in disease burden (DALYs per 100,000 population) and its percentile in per capita aid, calculated within income groups. The figure is analogous to Figure 9 in the main paper, except that aid and disease burden are accumulated over 2020–2021. The figure shows: **a**, HIV/AIDS and sexually transmitted infections; **b**, respiratory infections and tuberculosis; **c**, nutritional deficiencies; **d**, neglected tropical diseases and malaria; **e**, maternal and neonatal disorders; **f**, enteric infections; **g**, mental disorders; **h**, neoplasms; **i**, cardiovascular diseases; **j**, sense organ diseases; **k**, diabetes and kidney diseases; **l**, substance use disorders; **m**, musculoskeletal disorders; **n**, neurological disorders; **o**, chronic respiratory diseases; **p**, digestive diseases; and **q**, skin and subcutaneous diseases. Country boundaries are from Natural Earth, 1:110m Admin 0 Countries (public domain). Abbreviations: DALYs, disability-adjusted life years.

Sensitivity analysis to account for funding effects during the COVID-19 pandemic

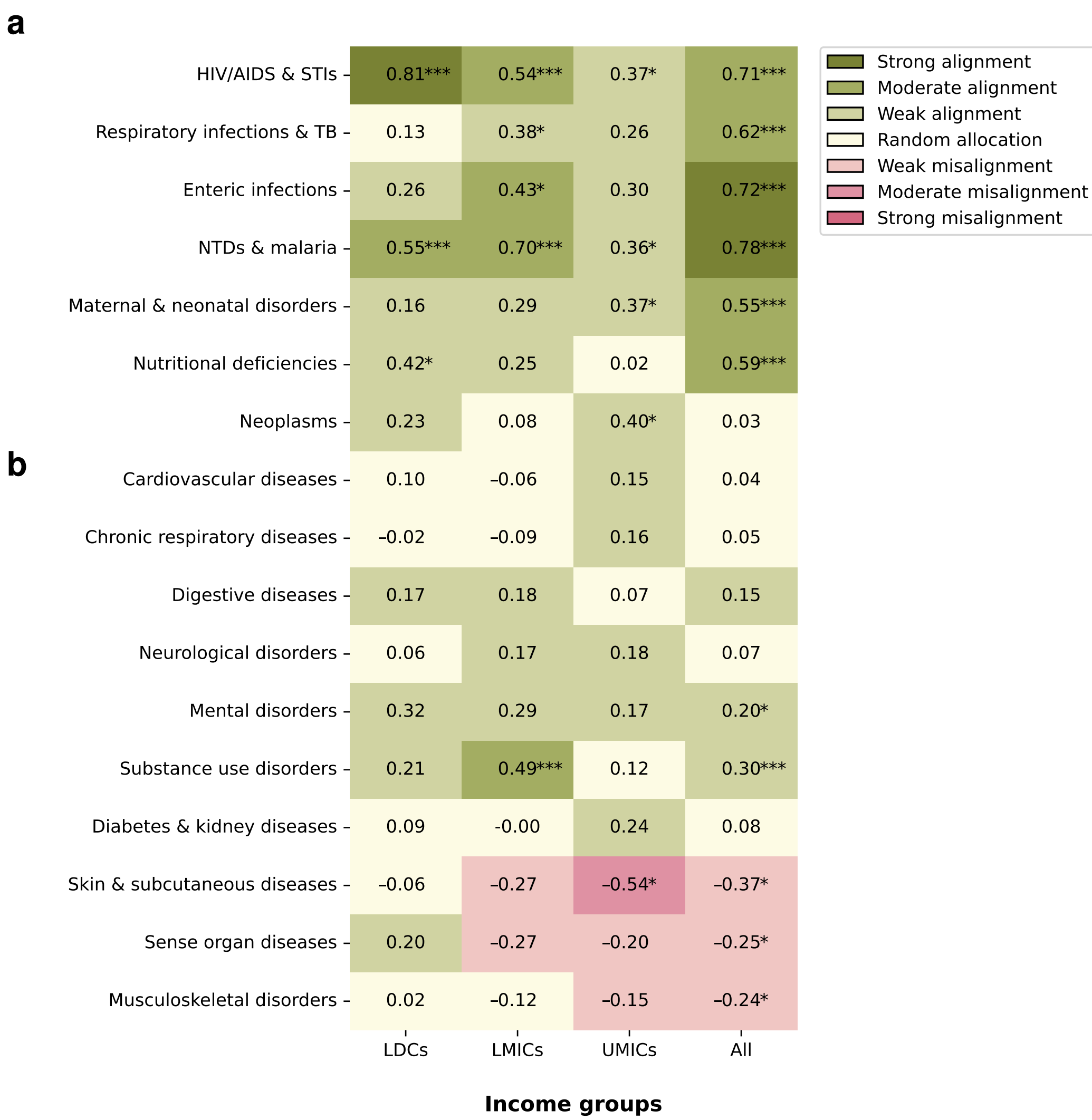

Fig. S7. Correlation between disease-specific aid and burden across income groups (as a sensitivity analysis discounting for the surge in aid earmarked to COVID-19). Shown are Spearman's rank correlation coefficients between per capita aid (accumulated over the time period 2000–2021) and disease burden across country income groups: least developed countries (LDCs), lower-middle-income countries (LMICs), upper-middle-income countries (UMICs), and all countries combined. The 2020 and 2021 values for respiratory infections and TB are replaced with the values for 2019. All other values are unchanged. **a**, The top six rows report CMNNDs. **b**, The bottom 11 rows show NCDs. Asterisks denote statistically significant correlations based on a two-sided Spearman rank test (* $p < 0.05$; ** $p < 0.01$; *** $p < 0.001$). Legend interpretation: -1 to -0.7: strong misalignment of health aid with disease burden; -0.7 to -0.3: moderate misalignment; -0.3 to -0.1: weak misalignment, -0.1 to 0.1: indicative of random allocation; 0.1 to 0.3: weak alignment, 0.3 to 0.7, moderate alignment; and 0.7 to 1.0 strong alignment. Abbreviations: CMNNDs, communicable, maternal, neonatal, and nutritional diseases; NCDs, non-communicable diseases; DALYs, disability-adjusted life years; STIs, sexually transmitted infections; TB, tuberculosis; NTDs: neglected tropical diseases.

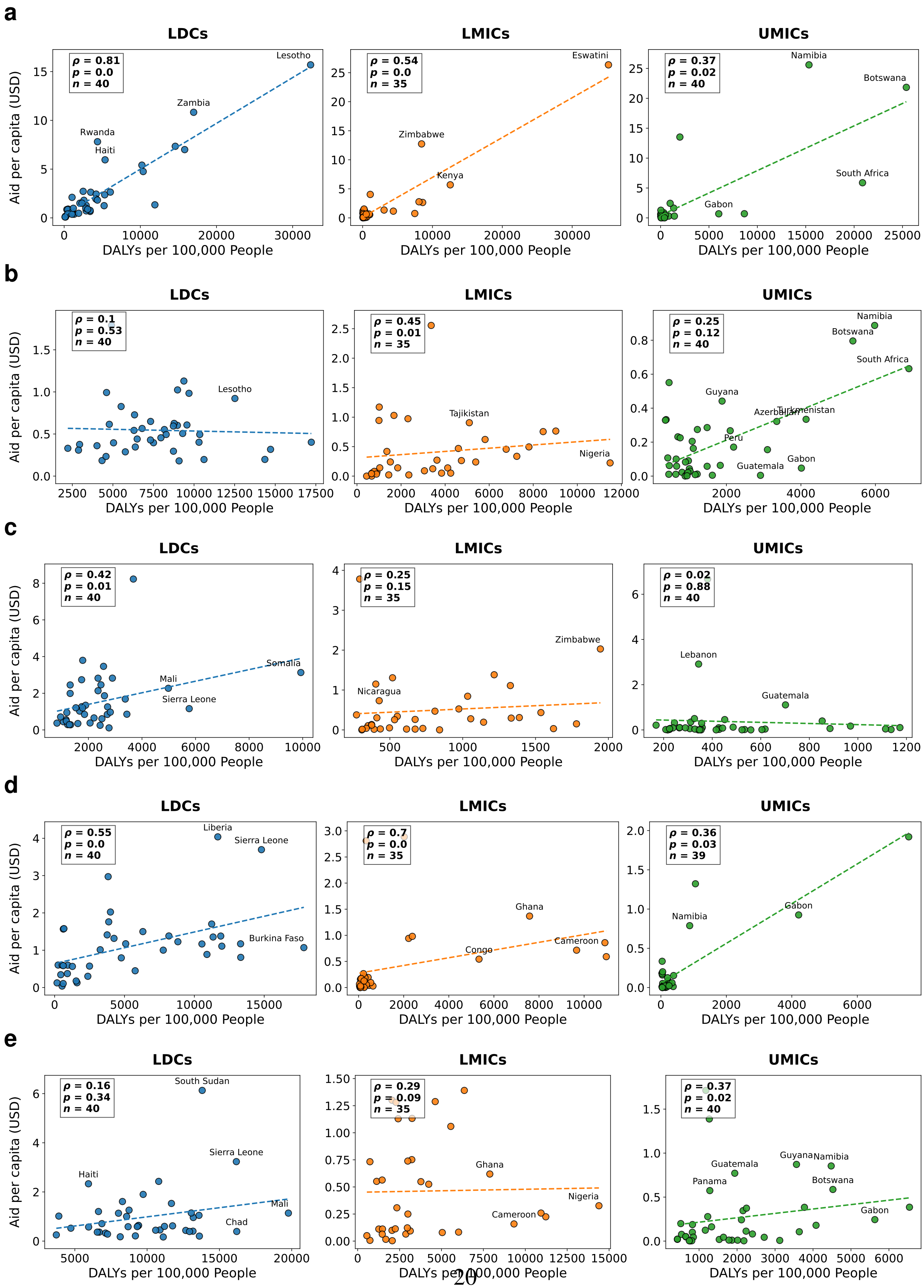
a
LDCs
ρ = 0.81
p = 0.0
n = 40
Lesotho
Zambia
Rwanda
Haiti
LMICs
ρ = 0.54
p = 0.0
n = 35
Eswatini
Zimbabwe
Kenya
UMICs
ρ = 0.37
p = 0.02
n = 40
Namibia
Botswana
South Africa
Gabon
b
LDCs
ρ = 0.1
p = 0.53
n = 40
Lesotho
LMICs
ρ = 0.45
p = 0.01
n = 35
Tajikistan
Nigeria
UMICs
ρ = 0.25
p = 0.12
n = 40
Namibia
Botswana
South Africa
Guyana
Azerbaijan
Turkmenistan
Peru
Gabon
Guatemala
c
LDCs
ρ = 0.42
p = 0.01
n = 40
Somalia
Mali
Sierra Leone
LMICs
ρ = 0.25
p = 0.15
n = 35
Zimbabwe
Nicaragua
UMICs
ρ = 0.02
p = 0.88
n = 40
Lebanon
Guatemala
d
LDCs
ρ = 0.55
p = 0.0
n = 40
Liberia
Sierra Leone
Burkina Faso
LMICs
ρ = 0.7
p = 0.0
n = 35
Ghana
Congo
Cameroon
UMICs
ρ = 0.36
p = 0.03
n = 39
Namibia
Gabon
e
LDCs
ρ = 0.16
p = 0.34
n = 40
South Sudan
Sierra Leone
Haiti
Mali
Chad
LMICs
ρ = 0.29
p = 0.09
n = 35
Ghana
Nigeria
Cameroon
UMICs
ρ = 0.37
p = 0.02
n = 40
Guyana
Namibia
Guatemala
Panama
Botswana
Gabon
Aid per capita (USD)
DALYs per 100,000 People

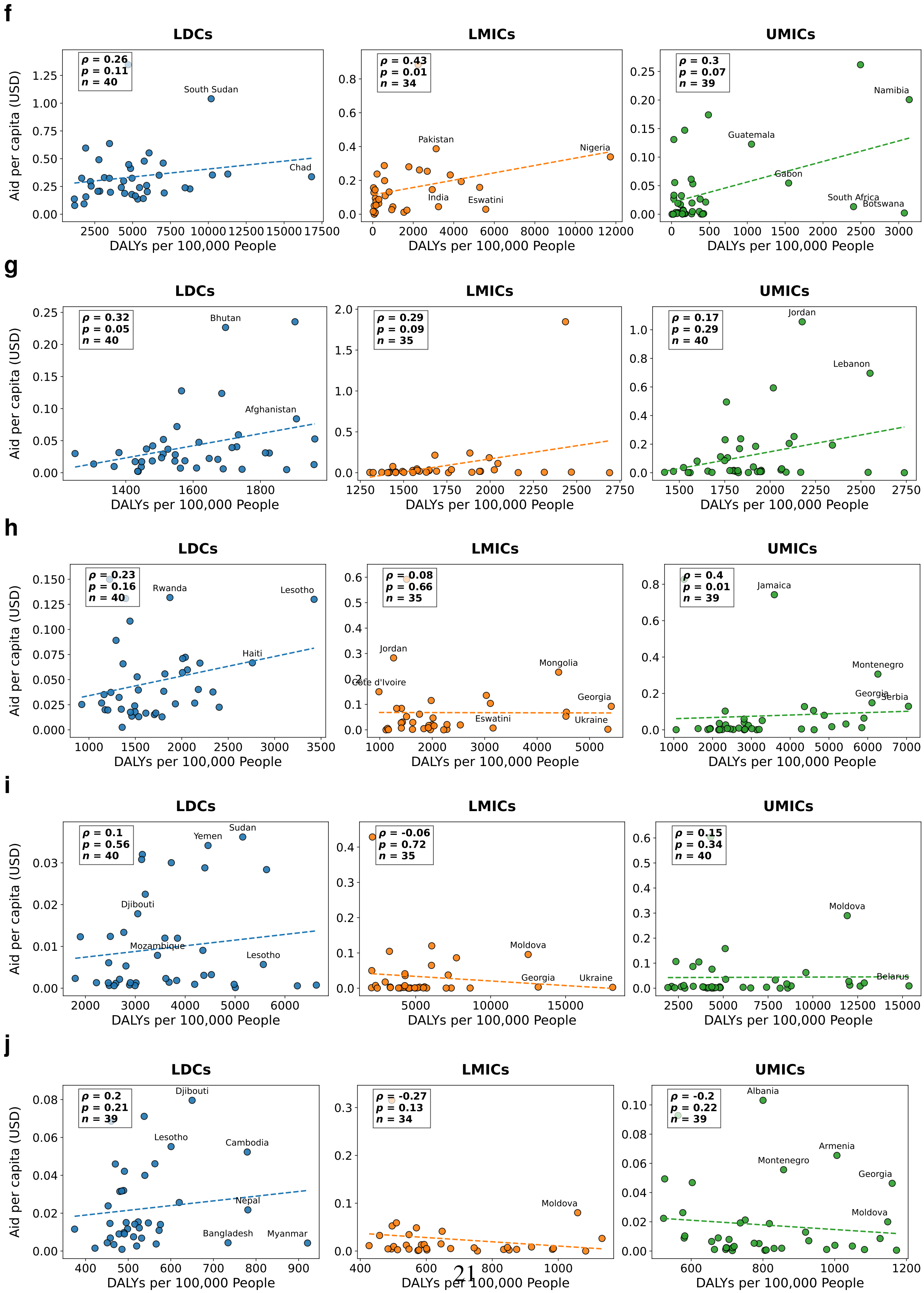
f
LDCs
ρ = 0.26
p = 0.11
n = 40
South Sudan
Chad
LMICs
ρ = 0.43
p = 0.01
n = 34
Pakistan
Nigeria
India
Eswatini
UMICs
ρ = 0.3
p = 0.07
n = 39
Namibia
Guatemala
Gabon
South Africa
Botswana
g
LDCs
ρ = 0.32
p = 0.05
n = 40
Bhutan
Afghanistan
LMICs
ρ = 0.29
p = 0.09
n = 35
UMICs
ρ = 0.17
p = 0.29
n = 40
Jordan
Lebanon
h
LDCs
ρ = 0.23
p = 0.16
n = 40
Rwanda
Lesotho
Haiti
LMICs
ρ = 0.08
p = 0.66
n = 35
Jordan
Côte d'Ivoire
Mongolia
Georgia
Eswatini
Ukraine
UMICs
ρ = 0.4
p = 0.01
n = 39
Jamaica
Montenegro
Georgia
Serbia
i
LDCs
ρ = 0.1
p = 0.56
n = 40
Sudan
Yemen
Djibouti
Mozambique
Lesotho
LMICs
ρ = -0.06
p = 0.72
n = 35
Moldova
Georgia
Ukraine
UMICs
ρ = 0.15
p = 0.34
n = 40
Moldova
Belarus
j
LDCs
ρ = 0.2
p = 0.21
n = 39
Djibouti
Lesotho
Cambodia
Nepal
Bangladesh
Myanmar
LMICs
ρ = -0.27
p = 0.13
n = 34
Moldova
UMICs
ρ = -0.2
p = 0.22
n = 39
Albania
Armenia
Montenegro
Georgia
Moldova
Aid per capita (USD)
DALYs per 100,000 People

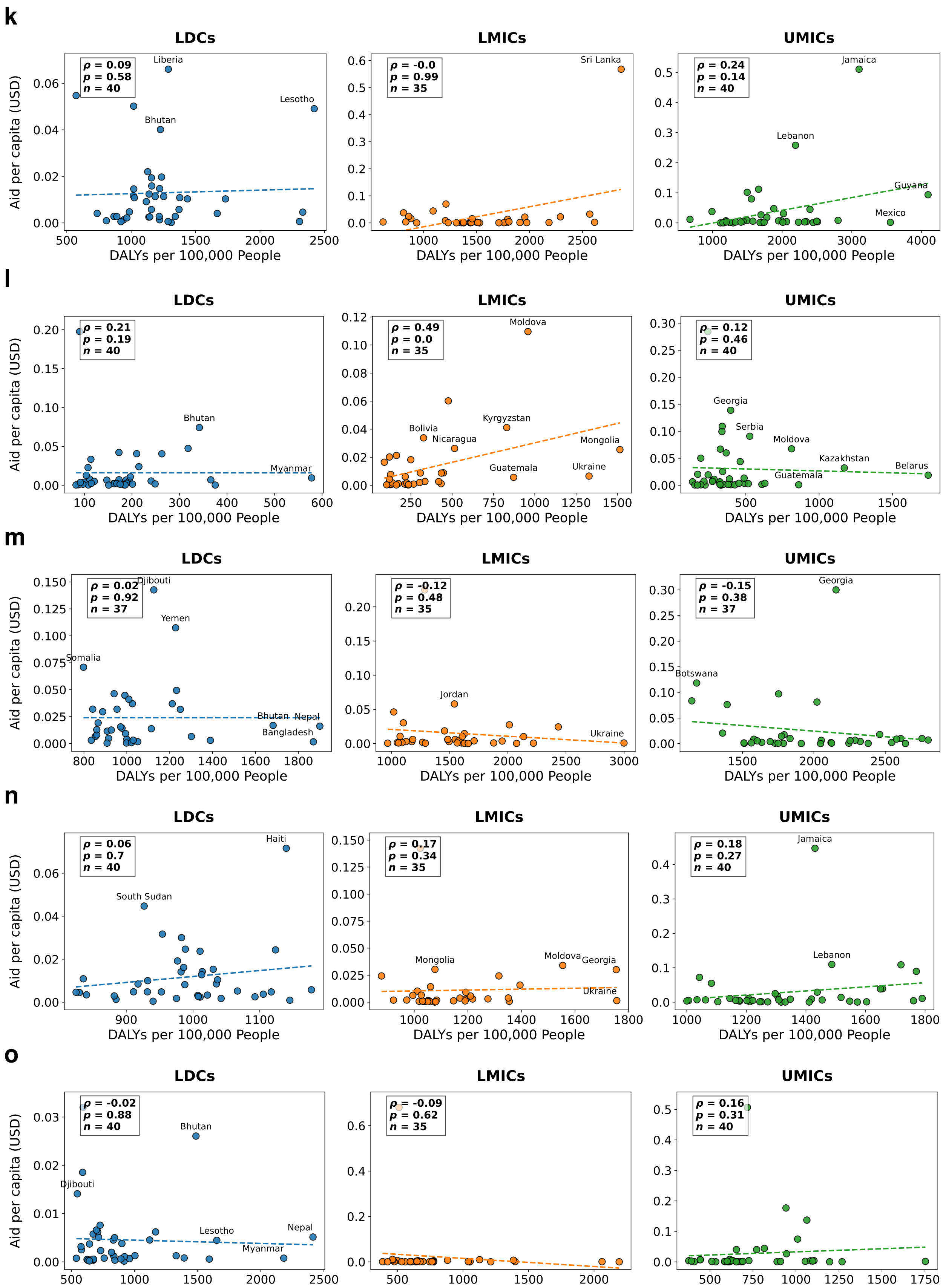
k
LDCs
ρ = 0.09
p = 0.58
n = 40
Liberia
Lesotho
Bhutan
LMICs
ρ = -0.0
p = 0.99
n = 35
Sri Lanka
UMICs
ρ = 0.24
p = 0.14
n = 40
Jamaica
Lebanon
Guyana
Mexico
l
LDCs
ρ = 0.21
p = 0.19
n = 40
Bhutan
Myanmar
LMICs
ρ = 0.49
p = 0.0
n = 35
Moldova
Kyrgyzstan
Bolivia
Nicaragua
Mongolia
Guatemala
Ukraine
UMICs
ρ = 0.12
p = 0.46
n = 40
Georgia
Serbia
Moldova
Kazakhstan
Belarus
Guatemala
m
LDCs
ρ = 0.02
p = 0.92
n = 37
Djibouti
Yemen
Somalia
Bhutan
Nepal
Bangladesh
LMICs
ρ = -0.12
p = 0.48
n = 35
Jordan
Ukraine
UMICs
ρ = -0.15
p = 0.38
n = 37
Georgia
Botswana
n
LDCs
ρ = 0.06
p = 0.7
n = 40
Haiti
South Sudan
LMICs
ρ = 0.17
p = 0.34
n = 35
Mongolia
Moldova
Georgia
Ukraine
UMICs
ρ = 0.18
p = 0.27
n = 40
Jamaica
Lebanon
o
LDCs
ρ = -0.02
p = 0.88
n = 40
Bhutan
Djibouti
Lesotho
Nepal
Myanmar
LMICs
ρ = -0.09
p = 0.62
n = 35
UMICs
ρ = 0.16
p = 0.31
n = 40
Aid per capita (USD)
DALYs per 100,000 People

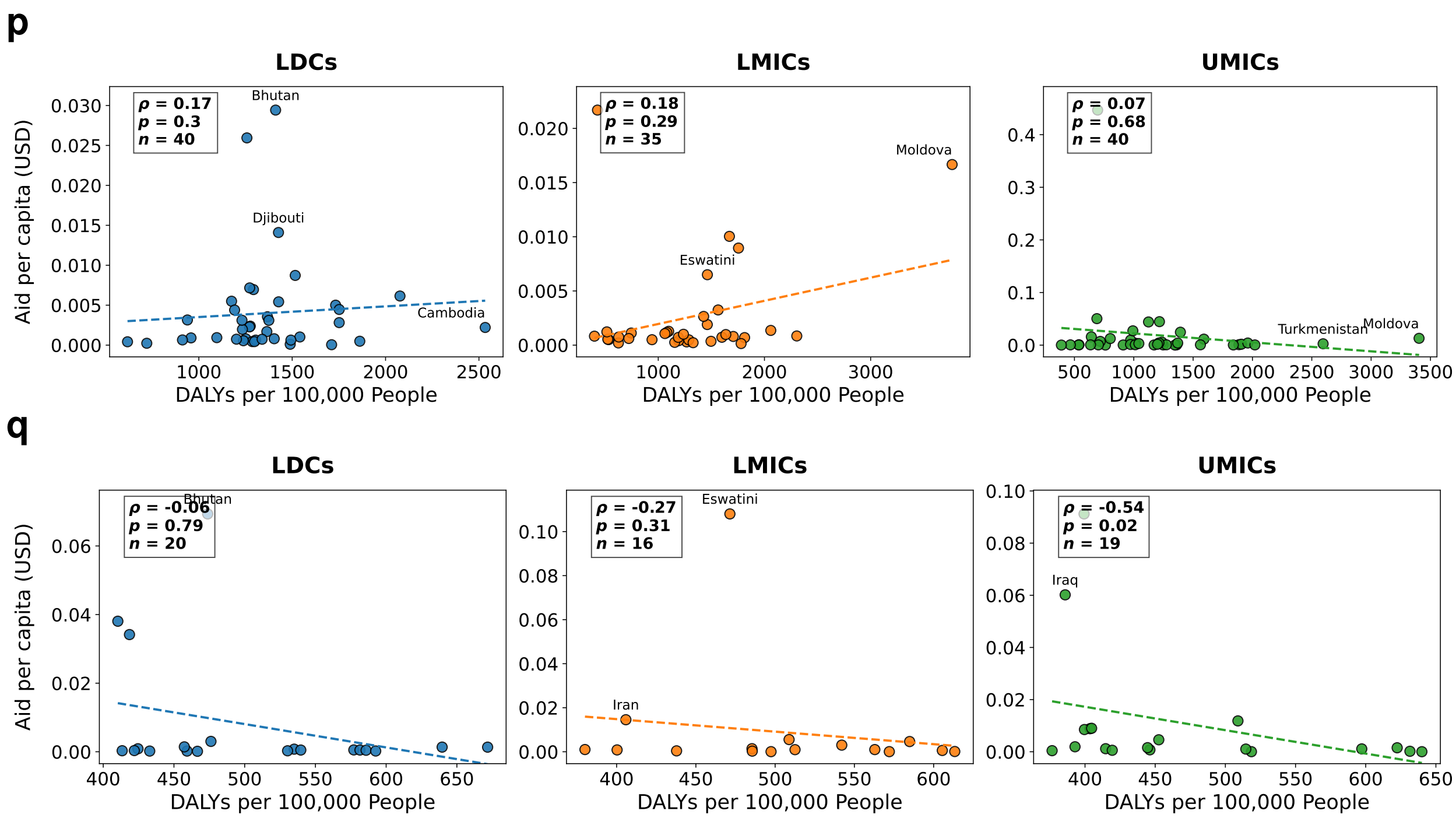

Fig. S8. Funding disparities at the country level (without the years 2020 and 2021 for respiratory infections and TB). The figure is analogous to Figure 8 in the main paper without the years 2020 and 2021 for respiratory infections and TB. The figure shows: **a**, HIV/AIDS and sexually transmitted infections; **b**, respiratory infections and tuberculosis; **c**, nutritional deficiencies; **d**, neglected tropical diseases and malaria; **e**, maternal and neonatal disorders; **f**, enteric infections; **g**, mental disorders; **h**, neoplasms; **i**, cardiovascular diseases; **j**, sense organ diseases; **k**, diabetes and kidney diseases; **l**, substance use disorders; **m**, musculoskeletal disorders; **n**, neurological disorders; **o**, chronic respiratory diseases; **p**, digestive diseases; and **q**, skin and subcutaneous diseases. Each plot reports Spearman's rank correlation coefficient (ρ), the corresponding p-value from a two-sided Spearman rank test, and the number of countries included in the analysis (n). The dashed line denotes the fitted trend line from an ordinary least squares regression. Abbreviations: DALYs, disability-adjusted life years.

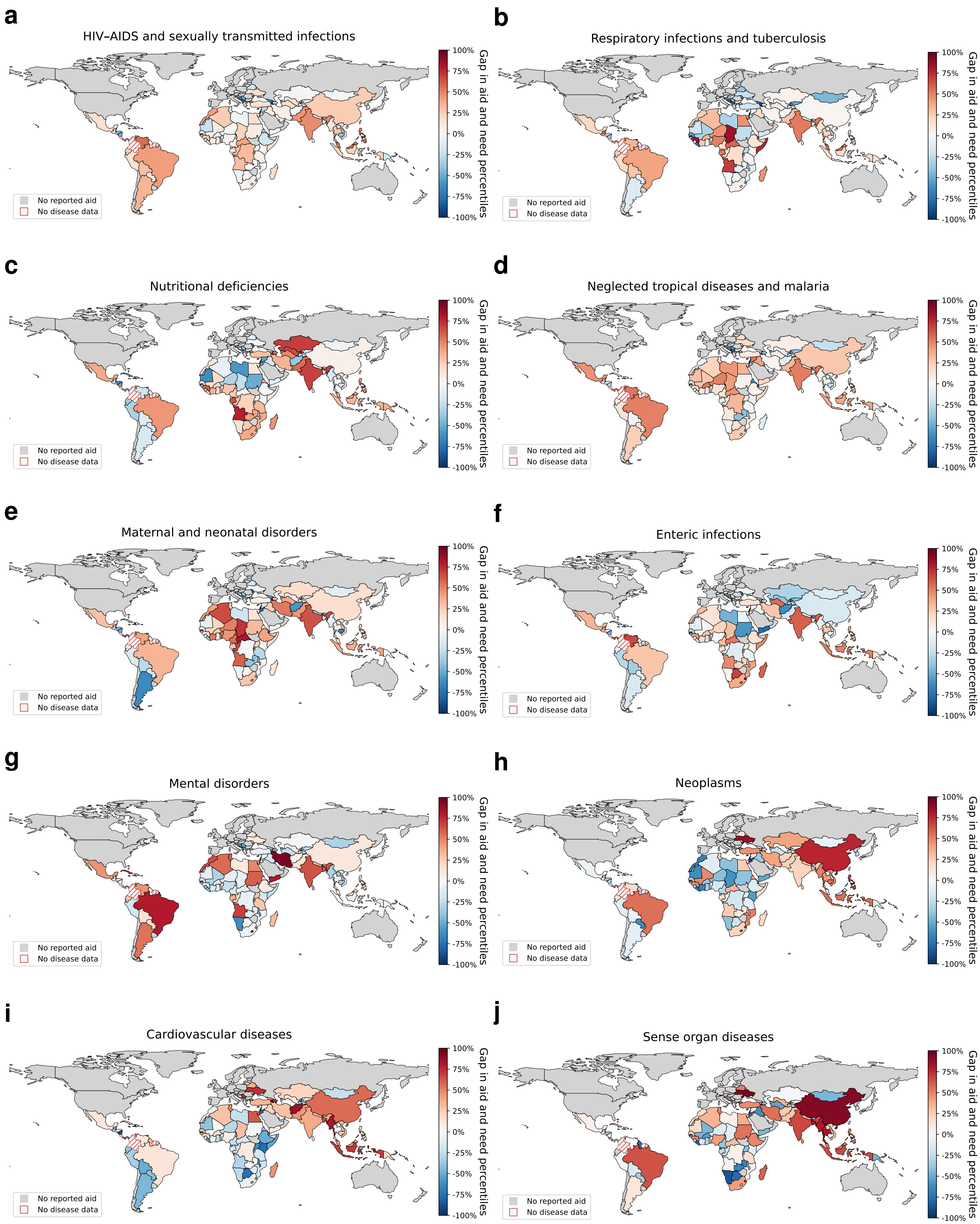
a
HIV–AIDS and sexually transmitted infections
b
Respiratory infections and tuberculosis
c
Nutritional deficiencies
d
Neglected tropical diseases and malaria
e
Maternal and neonatal disorders
f
Enteric infections
g
Mental disorders
h
Neoplasms
i
Cardiovascular diseases
j
Sense organ diseases
No reported aid
No disease data
100%
75%
50%
25%
0%
-25%
-50%
-75%
-100%
Gap in aid and need percentiles

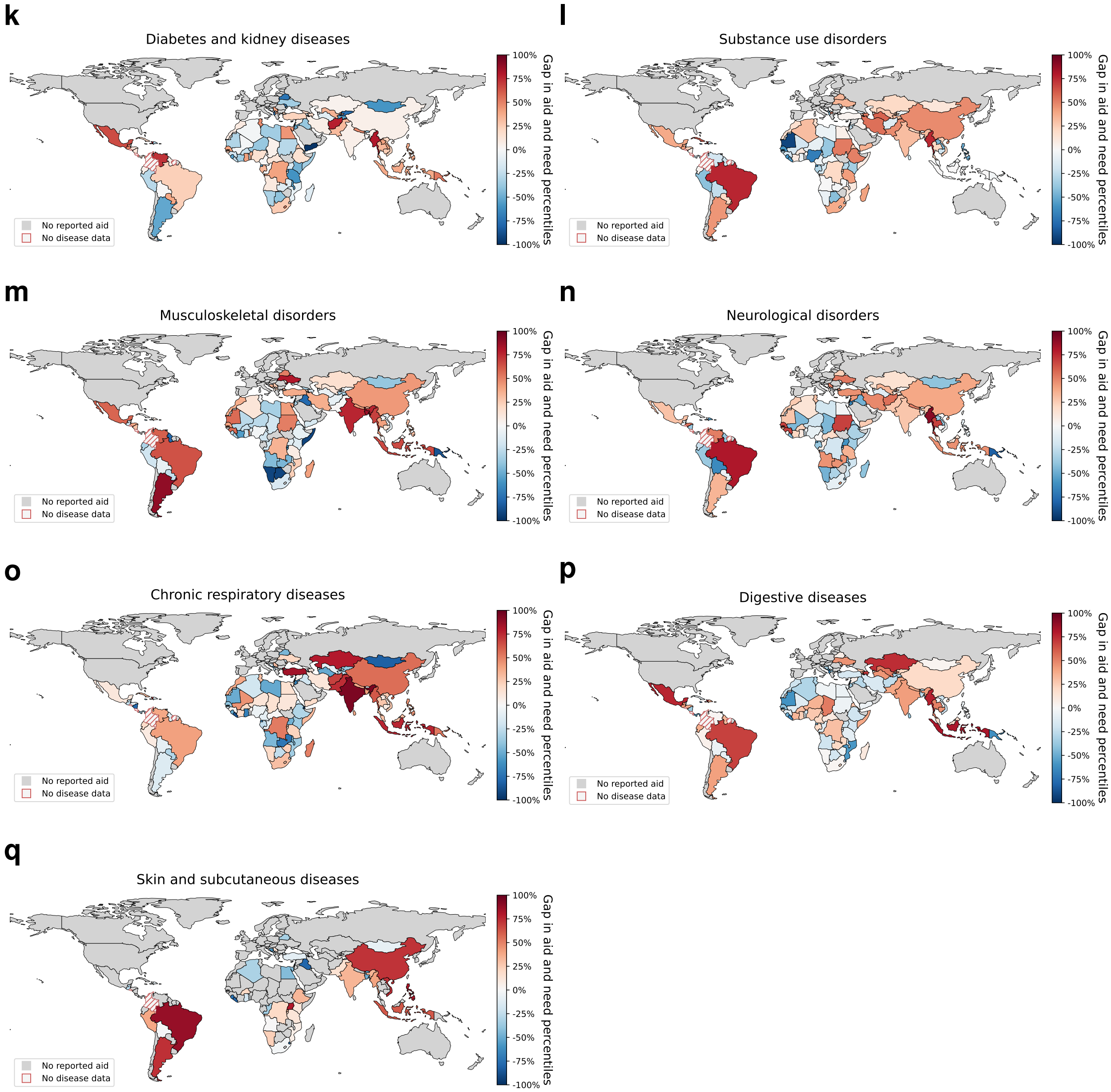

Fig. S9. Geographic distribution of aid–burden misalignment without the years 2020 and 2021 for respiratory infections and TB. The maps show the relative aid–burden misalignment by disease, calculated as the difference between a country's percentile in disease burden (DALYs per 100,000 population) and its percentile in per capita aid within income groups. The figure is analogous to Figure 9 in the main paper without the years 2020 and 2021 for respiratory infections and TB. The figure shows: **a**, HIV/AIDS and sexually transmitted infections; **b**, respiratory infections and tuberculosis; **c**, nutritional deficiencies; **d**, neglected tropical diseases and malaria; **e**, maternal and neonatal disorders; **f**, enteric infections; **g**, mental disorders; **h**, neoplasms; **i**, cardiovascular diseases; **j**, sense organ diseases; **k**, diabetes and kidney diseases; **l**, substance use disorders; **m**, musculoskeletal disorders; **n**, neurological disorders; **o**, chronic respiratory diseases; **p**, digestive diseases; and **q**, skin and subcutaneous diseases. Country boundaries are from Natural Earth, 1:110m Admin 0 Countries (public domain). Abbreviations: DALYs, disability-adjusted life years; TB, tuberculosis.

ODA project preprocessing and classification flow

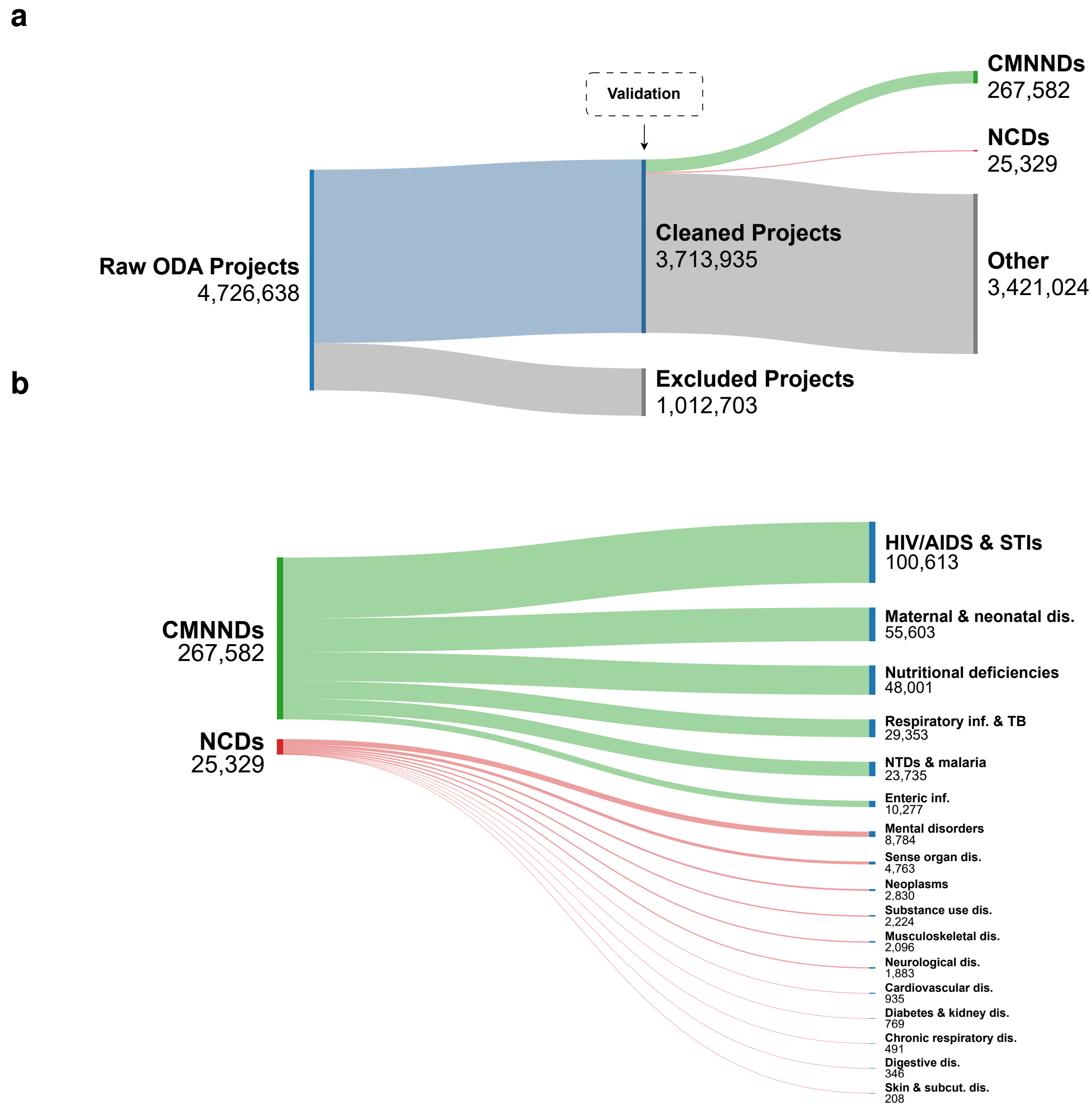

Fig. S10. ODA project preprocessing and classification flow. a, Flow of ODA projects from the raw corpus through preprocessing (i.e., removing invalid entries) to disease classification. Validation occurred after cleaning and before disease classification. **b**, Distribution of projects across the 17 disease categories. Ribbon width is proportional to the number of projects. Abbreviations: CMNNDs, communicable, maternal, neonatal, and nutritional diseases; NCDs, non-communicable diseases; STIs, sexually transmitted infections; TB, tuberculosis; NTDs, neglected tropical diseases.

Supplementary Materials

S1 Comparison against validation datasets

To assess the accuracy and reliability of our ML approach, we employed a dual validation strategy. First, we benchmarked our approach against a conventional keyword-based classification method (inspired by, e.g., [3]), which is interpretable, but unable to capture complex semantics as effectively as our LLM approach. Second, we manually annotated 2,060 aid projects and compared the labels to the LLM-generated classifications; however, this approach lacks the scalability of our machine learning pipeline.

S1.1 Comparison against keyword-based approach

Keyword list. To identify disease-related projects, we developed comprehensive keyword lists for each disease category. For this, we drew upon multiple authoritative sources, including subcategories from the Global Burden of Disease study, the PubMed Medical Subject Headings (MeSH) [4], and well-known disease synonyms. We selected keywords carefully to ensure they are specific to each disease category, so that we avoid overly broad terms that might include unrelated projects (i.e., to reduce false positives). For example, we considered the term "TB" for tuberculosis, but we excluded it because it often appears in unrelated contexts. To ensure the accuracy and relevance of our search terms, we consulted a panel of three experts (two public health researchers and one medical professional) to review and approve the final keyword lists.

To illustrate our approach, we elaborate our approach for the keywords that we selected for the category nutritional deficiencies. The keywords for this disease category include terms such as `malnutrition`, `undernutrition`, `stunting`, and specific deficiencies such as `iodine deficiency` and `zinc deficiency`. We derived these keywords from Global Burden of Disease (GBD) study subcategories, to ensure our search terms align closely with the study's health

categories. For instance, the GBD subcategories *"A.7.3 Vitamin A deficiency"* and *"A.7.4 Dietary iron deficiency"* inform our inclusion of the terms `vitamin A deficiency` and `iron deficiency`.

We provide the full list of keywords in Supplementary Material S5 and in our GitHub repository. For illustration purposes, we list the keywords for HIV/AIDS and STIs, including a brief justification, in Supplementary Table S3).

**Keyword**	**Reasoning**
`sexually transmitted infection`	Direct descriptor of the subcategory
`STI / STIs`	Acronym for sexually transmitted infection
`STD / STDs`	Acronym for sexually transmitted disease
`sexually transmitted disease`	Alternative term for sexually transmitted infection
`safe sex`	Practice to prevent transmission of STIs
`HIV`	Acronym for human immunodeficiency virus, the cause of AIDS
`AIDS`	Acronym for acquired immunodeficiency syndrome, which is caused by HIV
`acquired immunodeficiency syndrome`	Full name of AIDS
`antiretroviral`	Type of medication used to treat HIV/AIDS
`human immunodeficiency virus`	Full name of HIV
`syphilis`	Name of the specific sexually transmitted infection
`treponema pallidum`	Scientific name of the bacterium causing syphilis
`chancre`	Primary symptom of syphilis infection
`chlamydial infection`	Name of the specific sexually transmitted infection
`chlamydia`	Common name for chlamydial infection
`gonococcal infection`	Name of the specific sexually transmitted infection
`gonorrhea`	Common name for gonococcal infection
`trichomoniasis`	Name of the specific sexually transmitted infection
`trichomonas vaginalis`	Scientific name of the parasite causing trichomoniasis
`genital herpes`	Name of the specific sexually transmitted infection
`genital ulcer`	Common symptom of genital herpes

Table S3: **Keywords used as search terms for HIV/AIDS and sexually transmitted infections.**

Classification. We then searched for exact matches (case-insensitive) within the title, long description, and short description of each project. Again, aid projects were assigned to one or more categories based on the matches. Projects without keyword match were classified as "Other".

Results. The comparison between the LLM-based and keyword-based classifications reveals substantial agreement across disease categories, as evidenced by a strong diagonal pattern in our comparative analysis (Supplementary Figure S11). This alignment is particularly pronounced for well-defined disease categories such as HIV/AIDS and STIs and Neglected tropical diseases. How-

ever, we observe some divergence between methods for closely related conditions, such as chronic respiratory diseases and respiratory infections and TB, which we attribute to that the keyword-based search may not fully capture the semantics of the underlying aid description, which we confirm empirically in the next section using our manually-annotated dataset.

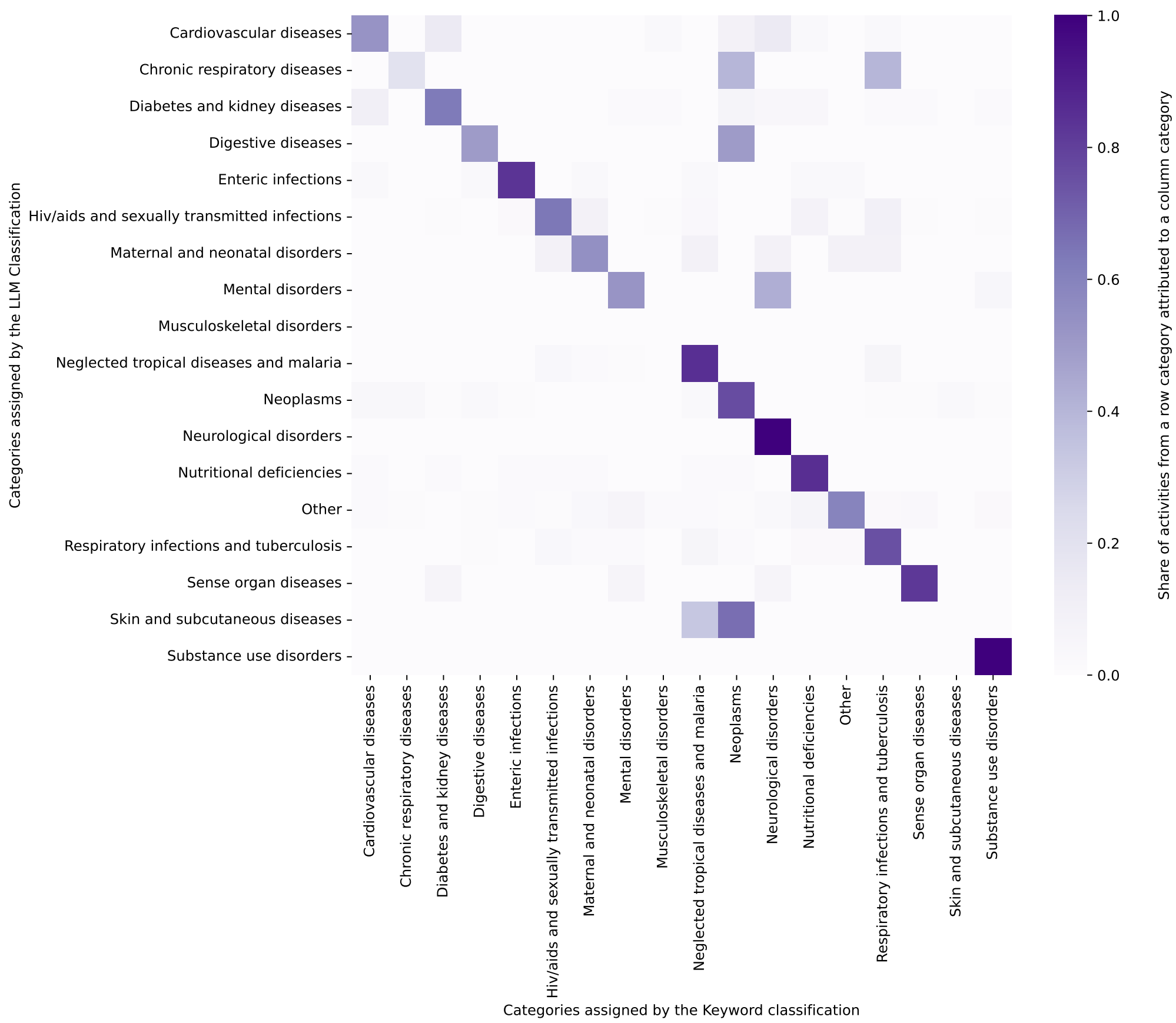

Fig. S11. Comparison between our machine learning pipeline based on LLMs and keyword-based classification. The comparison shows how aid projects ("activities") are classified into disease categories based on the machine learning approach (y-axis) and based on the keyword-based approach (x-axis). The cells show the percentage of aid projects from a specific disease category (as per machine learning pipeline) that are classified into a specific disease category (as per the keyword-based approach). An exception is musculoskeletal diseases, where we struggled to identify aid projects in the OECD CRS data using keywords. Upon manual inspection, this is often because simple keywords are not very meaningful in this context; this is confirmed in our comparison of the keyword-based approach against the manually-annotated validation dataset (see Supplementary Table S7). Therein, we find: On the one hand, the problems are due to the fact that the keyword-based approach struggles with classifying projects earmarked to musculoskeletal diseases (and not due to problems of our machine learning pipeline). As a case in point, the keyword-based approach has both a precision and recall of 0.00. On the other hand, this again highlights the benefits of our machine learning pipeline. Abbreviations: LLM, large language model.

S1.2 Comparison against manually-annotated dataset

Validation dataset. We manually classified a subset of 2,060 aid projects to compare the labels against our machine learning pipeline (and against the keyword-based approach). The validation set was drawn as a random sample from the full study corpus, rather than through stratification by disease category, funding amount, or donor. This design was intended to preserve the corpus-level distribution of projects in the validation set. We verified ex post that all disease categories were represented and that the validation set closely resembled the full corpus in the distribution of project text lengths (Supplementary Figure S12). The final sample size was chosen to balance the cost of manual annotation with the need for statistically precise performance estimates. Of the 17 disease categories, 12 had at least 50 annotated projects; among the remaining 5 categories, 4 had at least 20 projects, and one had 19 projects (Supplementary Table S4). Thus, coverage was substantial for most categories, while the smallest categories were represented by fewer projects because they are rare in the underlying corpus. Consistent with this, the model assigned projects to these rare categories in the full corpus at rates broadly proportional to their prevalence. A sensitivity analysis demonstrates that the size of the validation dataset is sufficient to obtain robust estimates of classification performance (Supplementary Figure S13).

To ensure precise annotations, we excluded water, sanitation, and hygiene (WASH) projects unless they explicitly focus on enteric conditions such as cholera. As expected, there is a subset of projects that are not relevant to specific disease categories (e.g., as they focus on other sustainability targets such as climate action) and are thus labeled as "Others". We also see frequencies that reflect the overall funding priorities (e.g., there are generally more labeled samples for CMNNDs than for NCDs).

Manual annotation was conducted by a primary annotator with domain knowledge in global health metrics and development aid projects, and independently classified by a second annotator. We report inter-annotator agreement using Cohen's kappa (κ) and label consistency rate (LCR),

i.e., the proportion of items receiving identical labels across annotators. To obtain a 95% confidence interval for κ, we applied a non-parametric bootstrap: we resampled the paired annotation labels with replacement 1,000 times, recomputed κ for each bootstrap sample, and took the central 95% of the resulting distribution as the bounds of the interval. For the LCR, we treated the number of matching labels as a binomial count and derived 95% confidence intervals using the Wilson score method, which yields well-calibrated intervals based on the observed proportion and the total number of annotated items. We observe substantial agreement across annotators ($\kappa = 0.91,\ 95\%\,\mathrm{CI}:[0.90,\,0.93];\ \mathrm{LCR} = 0.94,\ 95\%\,\mathrm{CI}:[0.93,\,0.95]$), which strengthens confidence in the reliability of the manually-annotated validation dataset. Discrepancies were subsequently resolved by a third annotator, and the resulting labels were used for the validation analyses. A small number of disagreements reflected borderline cases involving broad or cross-cutting health projects, which suggests some inherent ambiguity in the underlying project descriptions and category boundaries. A category-specific confusion matrix is shown in Supplementary Figure S14.

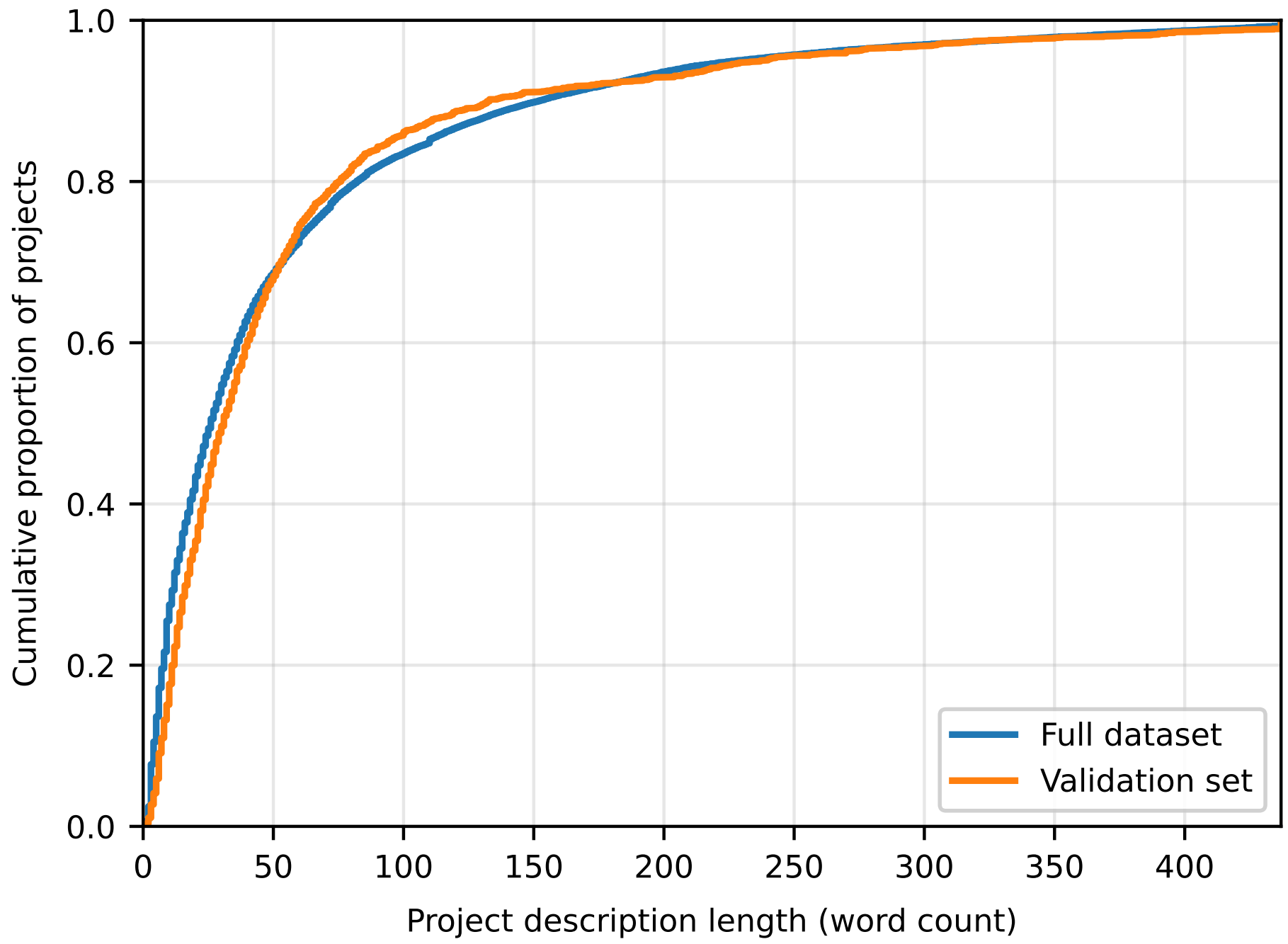

Fig. S12. Comparison of project description lengths in the full and validation datasets. The figure shows the empirical cumulative distribution of project description length (word count) for all projects in the full study corpus (blue) and for the manually annotated validation set (orange). The two distributions closely overlap, indicating that the validation set closely resembles the full corpus with respect to project description length, although the validation set contains slightly fewer very short descriptions. Summary statistics were similar across the two sets (full dataset: mean 56.9 words, median 26, IQR 9–66, 95th percentile 228; validation set: mean 58.2 words, median 31, IQR 14–61, 95th percentile 237).

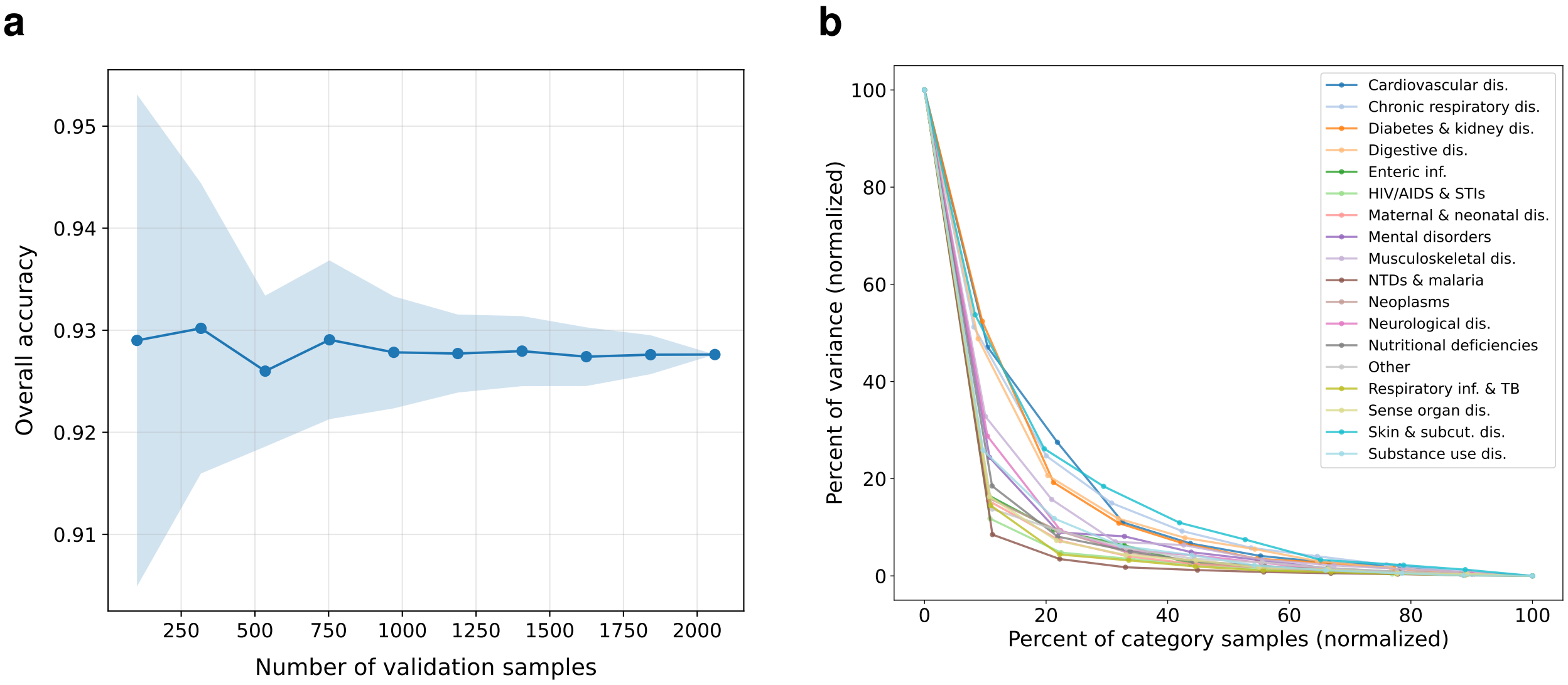

Fig. S13. Sensitivity analysis of classification performance. **a**, Convergence of overall classification accuracy as a function of the number of validation samples, where accuracy is summarized using the mean Jaccard score between predicted and manually assigned label sets. The line shows the mean across repeated random subsamples, and the shaded band reflects variability across subsamples. Performance stabilizes at approximately 0.93 once several hundred samples are included, indicating that the overall performance estimate is largely stable at the full validation set size ($n = 2{,}060$). **b**, Sensitivity analysis of category-level accuracy estimates, showing the variance of category-specific accuracy estimates versus the number of labeled projects per category. Both axes are min–max normalized to 0–100% within each category to enable comparison across categories with different sample sizes and variance ranges. The variance decreases sharply and stabilizes as per-category sample sizes approach those in the final validation set, which indicates increasing stability of the category-level performance estimates, although residual uncertainty remains for rare categories. Abbreviations: STIs, sexually transmitted infections; TB, tuberculosis; NTDs, neglected tropical diseases.

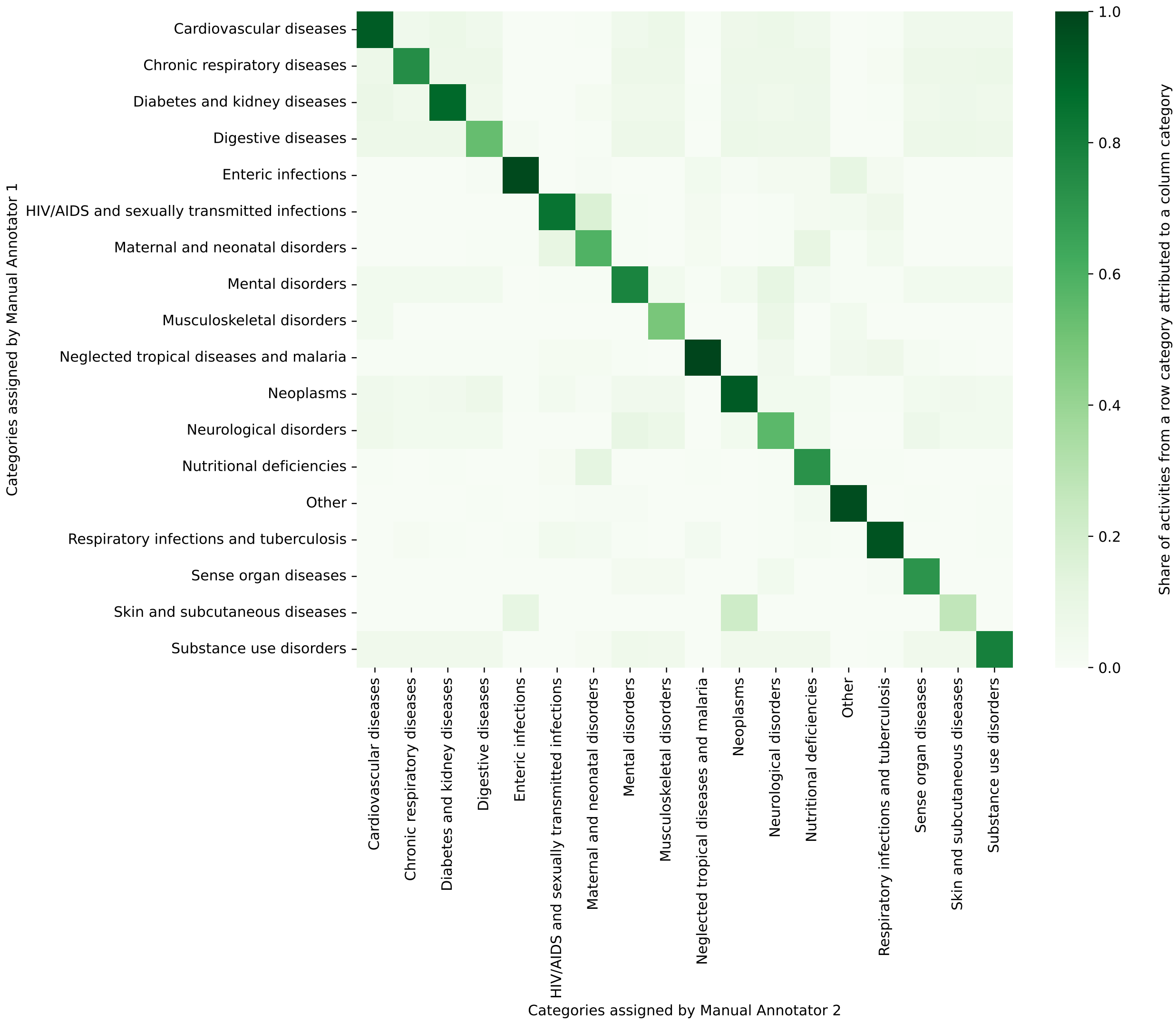

Fig. S14. Comparison between manual annotators. The comparison shows how aid projects ("activities") are classified into disease categories by Manual Annotator 1 (rows) and Manual Annotator 2 (columns). Each cell reports the share of activities from a row category that is assigned to the corresponding column category, so rows sum to 1. Values on the diagonal indicate agreement between annotators for a given disease category, while off-diagonal cells indicate disagreement.

Label	Frequency
Respiratory infections and tuberculosis	178
Nutritional deficiencies	142
Neglected tropical diseases and malaria	139
HIV/AIDS and sexually transmitted infections	105
Mental disorders	100
Maternal and neonatal disorders	94
Neoplasms	80
Sense organ diseases	76
Neurological disorders	74
Enteric infections	58
Substance use disorders	54
Cardiovascular diseases	51
Diabetes and kidney diseases	42
Musculoskeletal disorders	41
Chronic respiratory diseases	28
Digestive diseases	27
Skin and subcutaneous diseases	19
Other	992

Table S4: **Frequency of labels in the manually-annotated validation dataset.**

Performance metrics (overall). The validation of our machine learning approach against the manually-labeled dataset demonstrates the strong performance of our pipeline (Supplementary Table S5). In particular, our machine learning pipeline has a superior performance compared to the keyword-based approach: overall accuracy (0.91 vs 0.70), precision (0.94 vs 0.72), recall (0.89 vs 0.67), and F_1-score (0.92 vs 0.70). The superior performance extends to both micro- and macro-averaged performance metrics, indicating a reliable performance across both common and rare disease categories.

Performance metrics (stratified by disease categories). Further, we performed a stratified analysis of the performance by different disease categories for both our machine learning approach (Supplementary Table S6) and the keyword-based approach (Supplementary Table S7). The analysis confirms that the LLM method maintains high recall across the majority of disease categories, including neglected tropical diseases and malaria (0.98), respiratory infections and tuberculosis

Performance metric	Our ML approach	Keyword-based approach
Accuracy	0.91	0.70
Micro-averaged precision	0.94	0.72
Micro-averaged recall	0.89	0.67
Micro-averaged F_1-score	0.92	0.70
Macro-averaged precision	0.91	0.83
Macro-averaged recall	0.82	0.42
Macro-averaged F_1-score	0.86	0.48

Table S5: **Overall classification performance based on manually-labeled validation data.** Abbreviations: ML, machine learning.

(0.97), nutritional deficiencies (0.96), and enteric infections (0.93). In contrast, the keyword-based approach shows clear limitations, including large failure rates (e.g., a precision and recall of both 0.00) in classifying musculoskeletal disorders and skin and subcutaneous diseases, and very low recall for chronic respiratory diseases (0.07), digestive diseases (0.07), maternal and neonatal disorders (0.07), substance use disorders (0.09), and mental disorders (0.11).

Disease category	Precision	Recall	True positives
Cardiovascular diseases	0.98	0.94	48
Chronic respiratory diseases	0.91	0.75	21
Diabetes and kidney diseases	1.00	0.90	38
Digestive diseases	0.84	0.59	16
Enteric infections	0.87	0.93	54
HIV/AIDS and STIs	0.93	0.90	95
Maternal and neonatal disorders	0.92	0.71	67
Mental disorders	0.87	0.92	92
Musculoskeletal disorders	0.64	0.51	21
Neglected tropical diseases and malaria	0.94	0.98	136
Neoplasms	0.94	0.91	73
Neurological disorders	0.93	0.88	65
Nutritional deficiencies	0.92	0.96	137
Other	0.98	0.92	908
Respiratory infections and tuberculosis	0.93	0.97	172
Sense organ diseases	0.91	0.78	59
Skin and subcutaneous diseases	1.00	0.37	7
Substance use disorders	0.92	0.85	46

Table S6: **Classification performance by disease categories for the machine learning approach.** Results are compared against the manually-annotated validation dataset.

Disease category	Precision	Recall	True positives
Cardiovascular diseases	0.70	0.37	19
Chronic respiratory diseases	0.40	0.07	2
Diabetes and kidney diseases	0.81	0.69	29
Digestive diseases	1.00	0.07	2
Enteric infections	0.91	0.52	30
HIV/AIDS and STIs	0.74	0.71	75
Maternal and neonatal disorders	0.88	0.07	7
Mental disorders	0.55	0.11	11
Musculoskeletal disorders	0.00	0.00	0
Neglected tropical diseases and malaria	0.96	0.86	119
Neoplasms	0.89	0.72	58
Neurological disorders	1.00	0.16	12
Nutritional deficiencies	0.93	0.30	42
Other	0.67	0.99	979
Respiratory infections and tuberculosis	0.86	0.82	146
Sense organ diseases	0.93	0.18	14
Skin and subcutaneous diseases	0.00	0.00	0
Substance use disorders	1.00	0.09	5

Table S7: **Classification performance by disease categories for the keyword-based approach.** Results are compared against the manually-annotated validation dataset.

Comparison (confusion matrix). We further compare how different aid projects in the manually-annotated dataset are classified in comparison to our machine learning approach (see Supplementary Figure S15 for a row-normalized confusion matrix) shows high precision and low misclassification rates, indicated by strong diagonal dominance with minimal off-diagonal elements. Where misclassification occurs, it primarily appears between clinically related conditions.

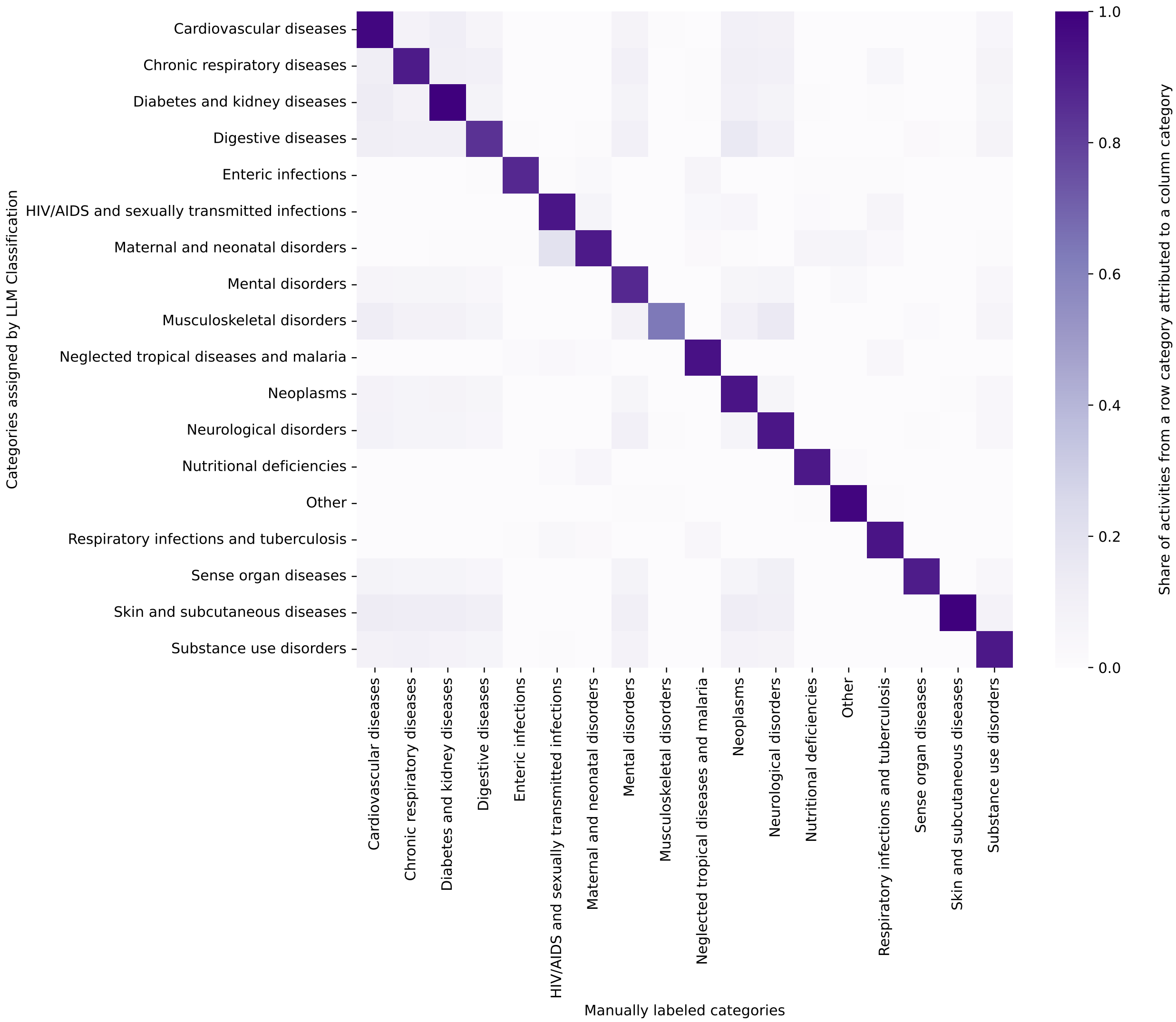

Fig. S15. Comparison between our machine learning pipeline and the manually-annotated dataset. The comparison shows how aid projects ("activities") are classified into disease categories based on our machine learning approach (y-axis) and based on the validation dataset (x-axis). The cells show the percentage of aid projects from a specific disease category (as per machine learning pipeline) that are classified into a specific disease category (as per the validation dataset). The diagonal represents the precision of our machine learning pipeline, while the other cells show the percentage of manually-annotated aid projects from one disease category that the LLM assigned to another category. Abbreviations: LLM, large language model.

S2 Robustness checks

S2.1 Robustness to random seeds and alternative models

To ensure the robustness of our approach, we quantified the uncertainty and reproducibility of the disease-classification pipeline for a randomly sampled 1% of projects. The distribution of disease categories in the 1% subset and in the full corpus is reported in Supplementary Table S8. We repeated the classification with different random seeds and with alternative LLMs (GPT-4.1-mini-2025-04-14 and DeepSeek-V3.1) instead of the main model (Llama-3.1-70B-Instruct). Because LLM performance can vary across tasks and different models may exhibit different strengths and weaknesses, we assessed the sensitivity of the main classification patterns to model choice. We selected GPT-4.1-mini-2025-04-14 as an external benchmark that is suitable for large-scale classification and allows comparison with a proprietary model from a different developer than our primary pipeline. Within the GPT-4.1 family, the mini variant offers a suitable balance between performance and scalability for large-scale classification. It shows strong reported benchmark performance, supports a 1M-token context window, and has lower latency than larger models in the same family [5, 6]. We thus used it to assess whether the main classification patterns persist in a strong and computationally efficient proprietary alternative that is realistic for large-scale classification. We selected DeepSeek-V3.1 as a second external benchmark to extend the comparison to a third model developer. Specifically, DeepSeek-V3.1 combines strong reported benchmark performance, a large architecture (671B total parameters), and a 128,000-token context window [7, 8]. It is open-weight and has comparatively low inference costs, which makes it suitable for large-scale applied use beyond proprietary models. Agreement across runs and models was summarized using Cohen's kappa (κ) and the label consistency rate (LCR), defined as the proportion of items receiving identical labels across runs. Across random seeds, we observed $\kappa = 0.88$ (95% CI: [0.87, 0.88]) and an LCR of 0.90 (95% CI: [0.89, 0.90]). Relative to the primary model Llama-3.1-70B-Instruct, GPT-4.1-mini-2025-04-14 yielded $\kappa = 0.88$ (95% CI: [0.88, 0.88]) and LCR =

0.90 (95% CI: [0.90, 0.90]), whereas DeepSeek-V3.1 yielded $\kappa = 0.71$ (95% CI: [0.70, 0.71]) and LCR = 0.75 (95% CI: [0.75, 0.76]). Category-specific confusion matrices (Supplementary Figures S16–S21) indicate that the lower agreement for DeepSeek-V3.1 is concentrated in a small number of categories, particularly in more frequent assignment to "Other", while the vast majority of projects receive the same labels for both LLMs. Together, these analyses indicate high run-to-run reproducibility and support the robustness of the main results across alternative LLMs, although robustness varies by model.

Disease category	1% robustness analysis subset		Full corpus (excluding "Other")	
	Count	%	Count	%
HIV/AIDS and sexually transmitted infections	13259	33.04	116349	31.78
Maternal and neonatal disorders	5873	14.63	67659	18.48
Nutritional deficiencies	5960	14.85	52159	14.25
Respiratory infections and tuberculosis	4482	11.17	40577	11.08
Neglected tropical diseases and malaria	3512	8.75	33138	9.05
Enteric infections	1535	3.82	14003	3.83
Mental disorders	1585	3.95	11958	3.27
Sense organ diseases	699	1.74	4880	1.33
Substance use disorders	616	1.53	4339	1.19
Neoplasms	580	1.45	4163	1.14
Neurological disorders	510	1.27	3984	1.09
Cardiovascular diseases	372	0.93	3246	0.89
Diabetes and kidney diseases	312	0.78	2894	0.79
Musculoskeletal disorders	358	0.89	2405	0.66
Chronic respiratory diseases	259	0.65	2335	0.64
Digestive diseases	173	0.43	1647	0.45
Skin and subcutaneous diseases	48	0.12	334	0.09

Table S8: **Distribution of disease categories in the 1% subset and in the full corpus.** The 1% subset was used in the robustness analyses to assess the stability of the disease classification pipeline under alternative random seeds and LLMs. Counts and percentages are shown for the 1% subset and for all projects in the full corpus assigned to one of the disease categories listed here; projects classified as "Other" are therefore not included. Overall, the subset closely approximates the distribution of disease categories in the full corpus, although the share of maternal and neonatal disorders is 3.85 percentage points lower in the subset than in the full corpus. Rare categories, such as skin and subcutaneous diseases, are represented by fewer observations in the subset.

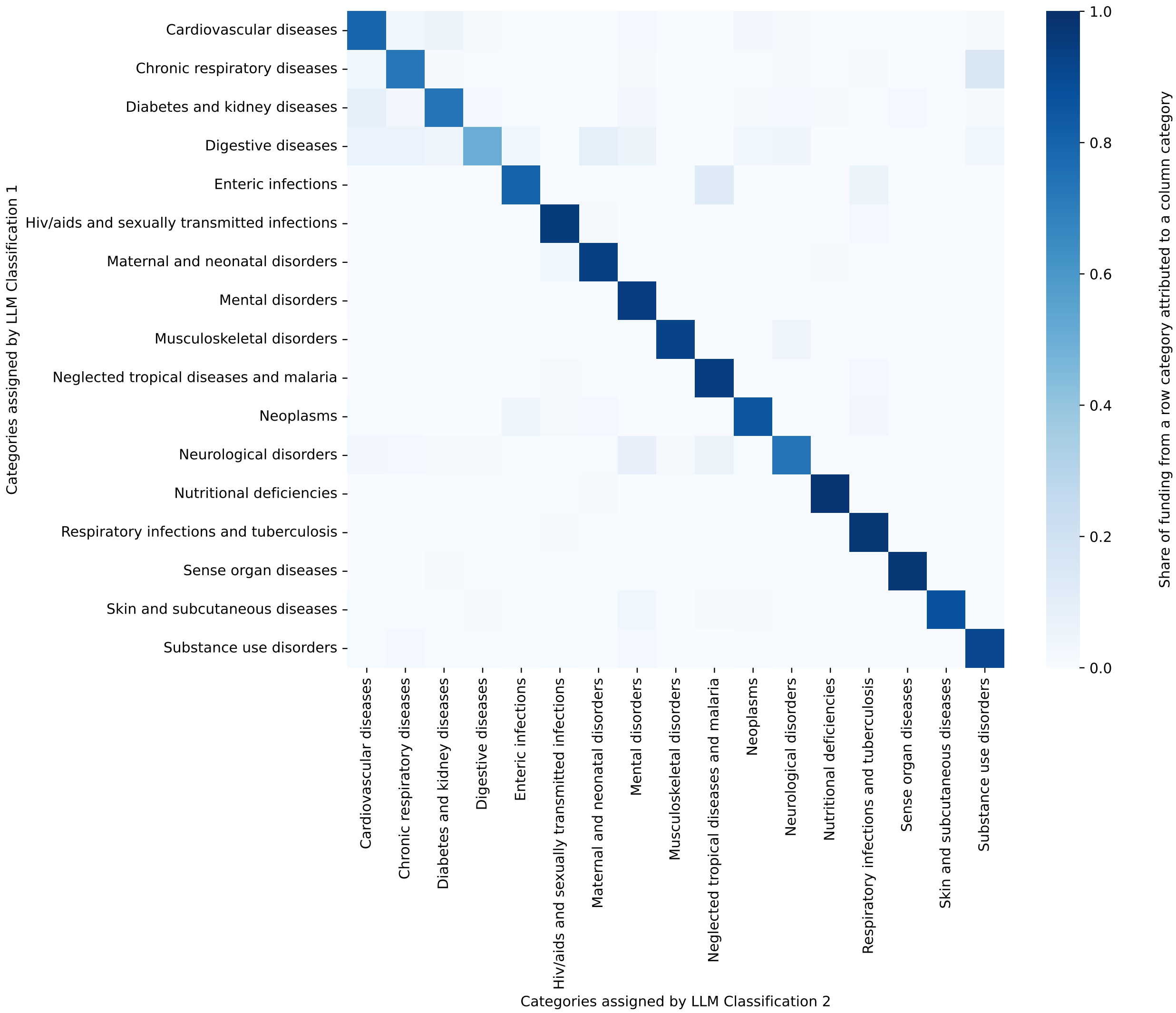

Fig. S16. Comparison between classifications from different random seeds, weighted by funding. The comparison shows how aid projects ("activities") are classified into disease categories by our machine learning pipeline using different random seeds. Each cell reports the share of activities from a row category that is assigned to the corresponding column category, weighted by funding, so rows sum to 1. Values on the diagonal indicate agreement across runs for a given disease category, while off-diagonal cells indicate disagreement. Abbreviations: LLM, large language model.

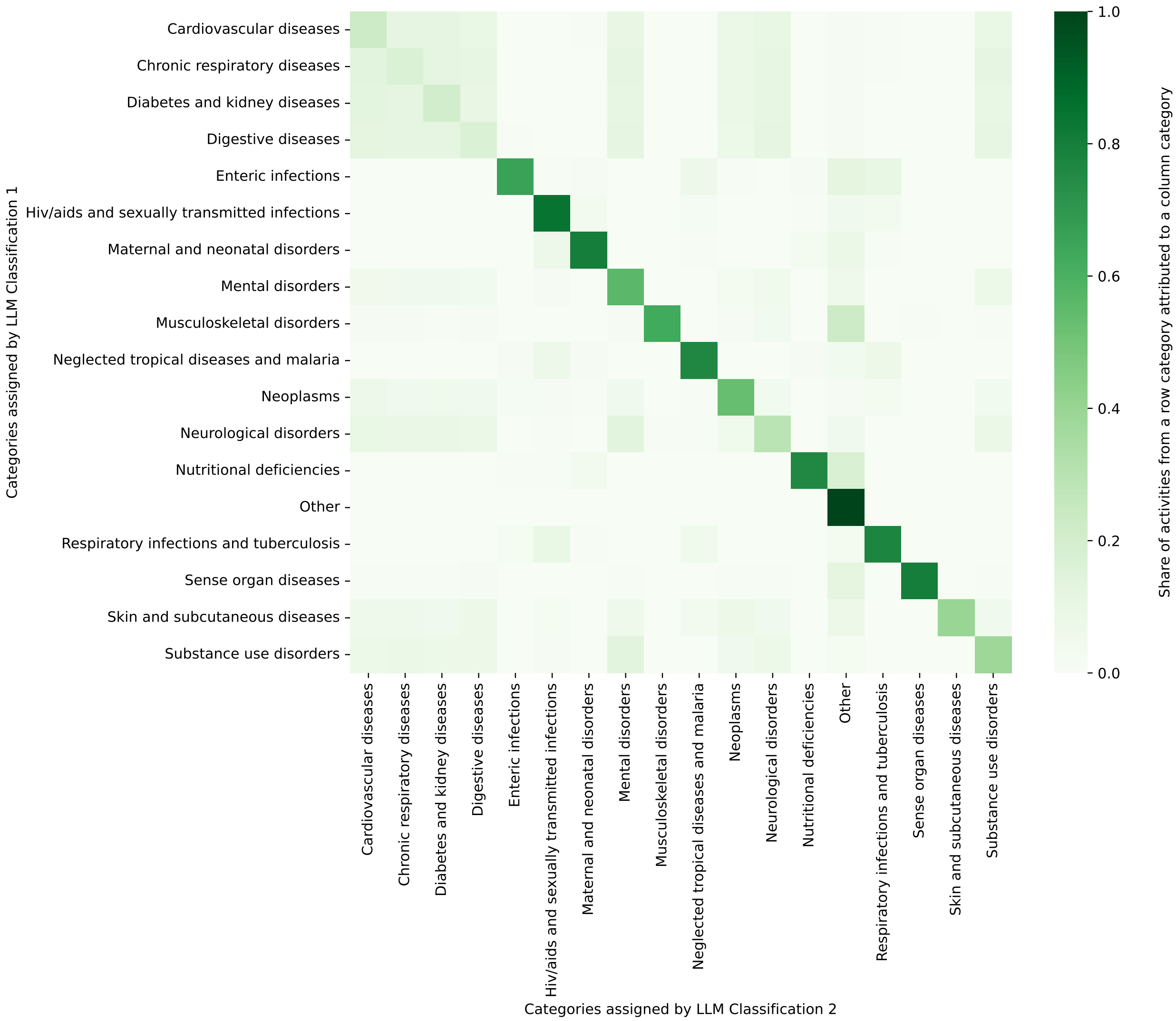

Fig. S17. Comparison between classifications from different random seeds. The comparison shows how aid projects ("activities") are classified into disease categories by our machine learning pipeline using different random seeds. Each cell reports the share of activities from a row category that is assigned to the corresponding column category, so rows sum to 1. Values on the diagonal indicate agreement across runs for a given disease category, while off-diagonal cells indicate disagreement. Abbreviations: LLM, large language model.

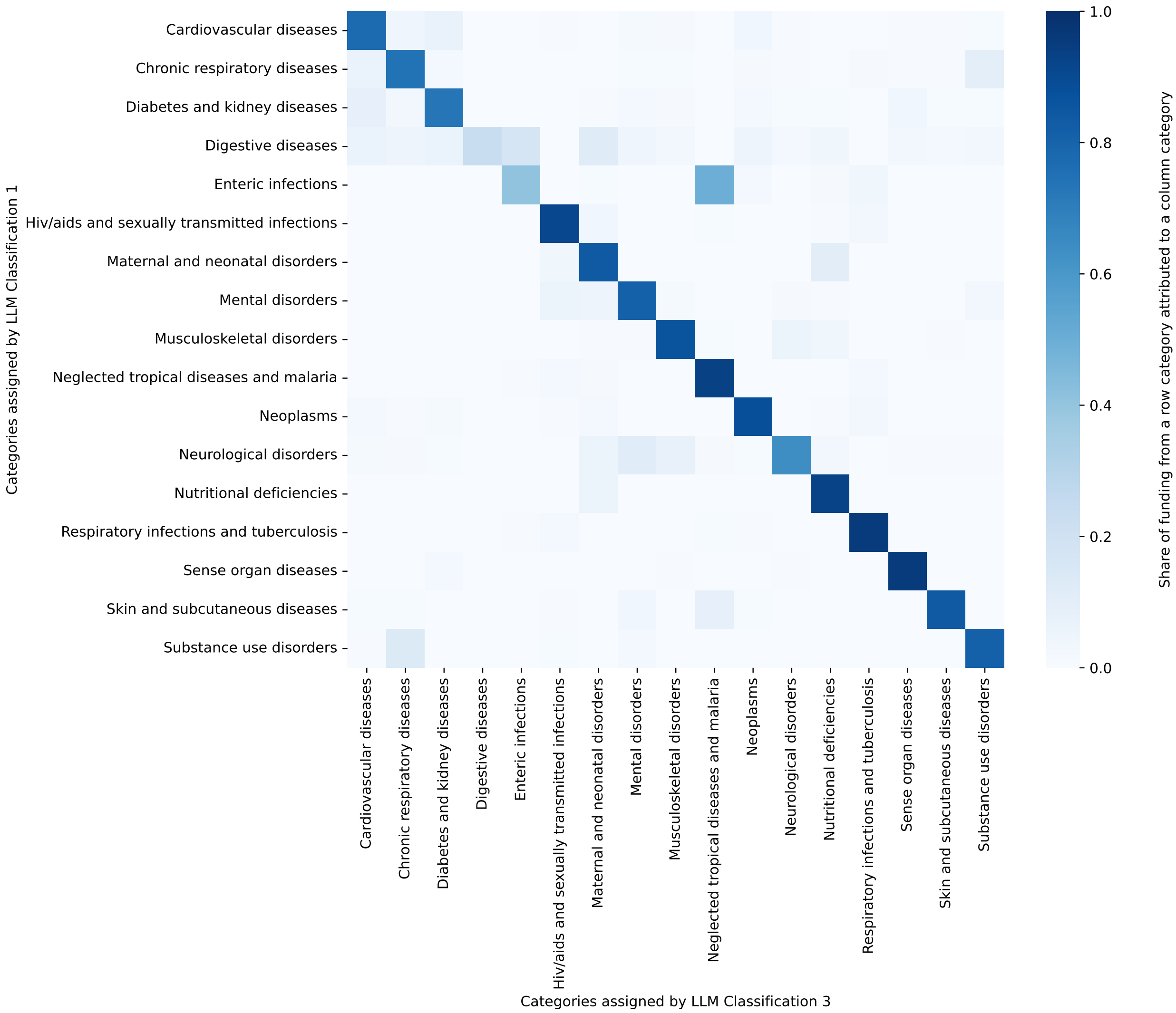

Fig. S18. Comparison between GPT-4.1-mini-2025-04-14 classifications and primary model classifications, weighted by funding. The comparison shows how aid projects ("activities") are classified into disease categories by our machine learning pipeline using different LLMs. LLM Classification 1 uses Llama-3.1-70B-Instruct (rows), LLM Classification 3 uses GPT-4.1-mini-2025-04-14 (columns). Each cell reports the share of activities from a row category that is assigned to the corresponding column category, weighted by funding, so rows sum to 1. Values on the diagonal indicate agreement across models for a given disease category, while off-diagonal cells indicate disagreement. Abbreviations: LLM, large language model.

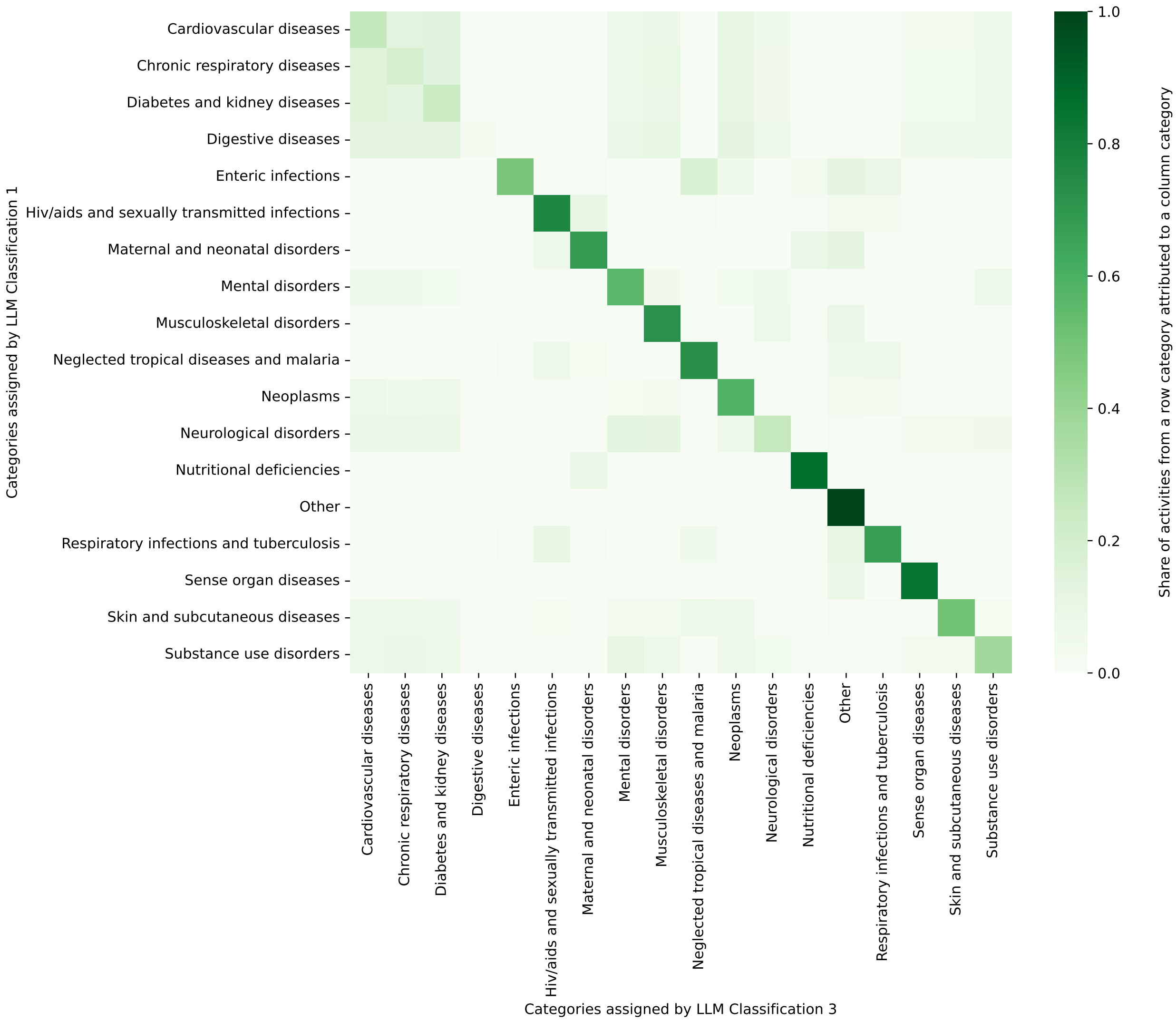

Fig. S19. Comparison between GPT-4.1-mini-2025-04-14 classifications and primary model classifications. The comparison shows how aid projects ("activities") are classified into disease categories by our machine learning pipeline using different LLMs. LLM Classification 1 uses Llama-3.1-70B-Instruct (rows), LLM Classification 3 uses GPT-4.1-mini-2025-04-14 (columns). Each cell reports the share of activities from a row category that is assigned to the corresponding column category, so rows sum to 1. Values on the diagonal indicate agreement across models for a given disease category, while off-diagonal cells indicate disagreement. Abbreviations: LLM, large language model.

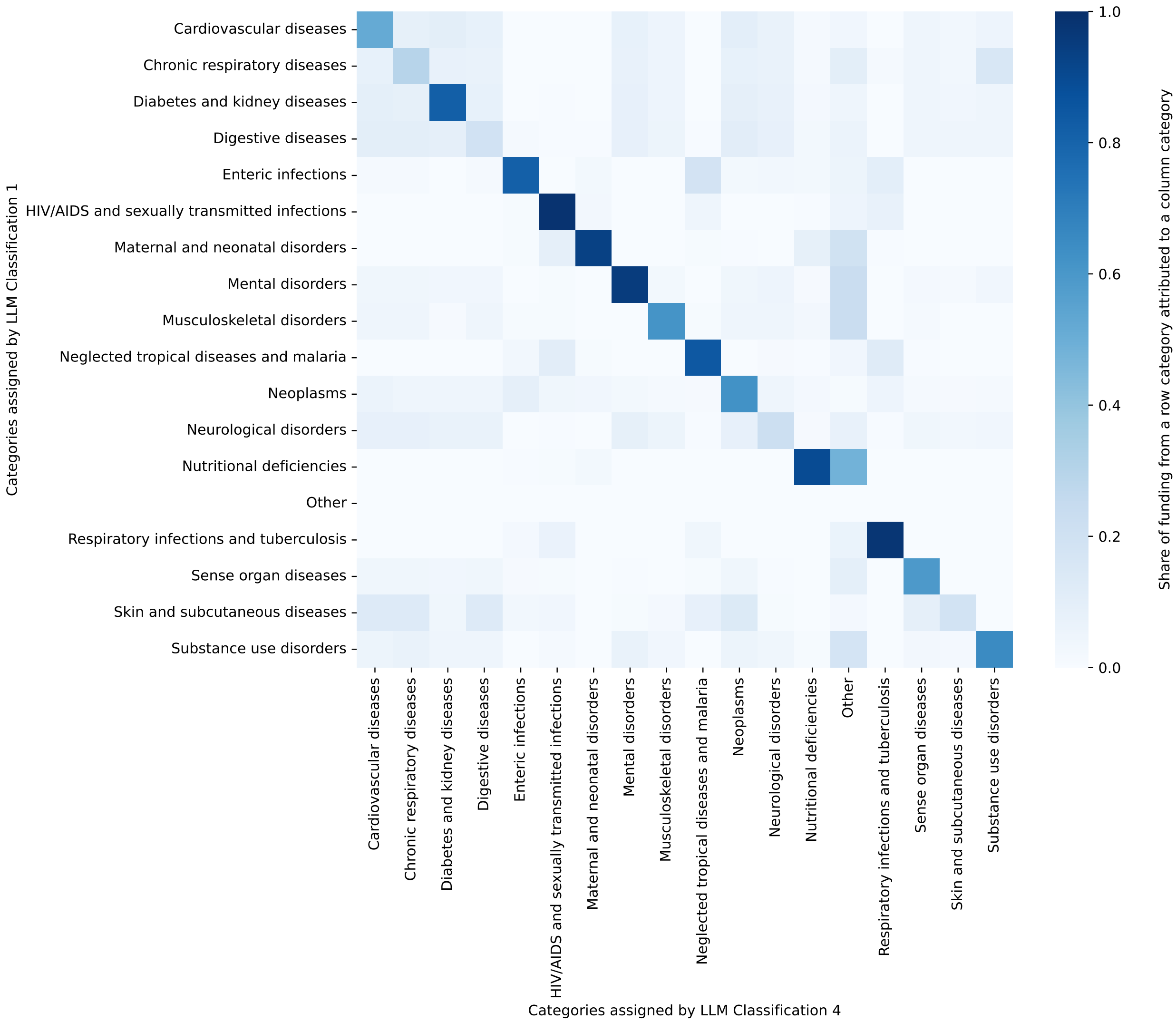

Fig. S20. Comparison between DeepSeek-V3.1 classifications and primary model classifications, weighted by funding. The comparison shows how aid projects ("activities") are classified into disease categories by our machine learning pipeline using different LLMs. LLM Classification 1 uses Llama-3.1-70B-Instruct (rows), LLM Classification 4 uses DeepSeek-V3.1 (columns). Each cell reports the share of activities from a row category that is assigned to the corresponding column category, weighted by funding, so rows sum to 1. Values on the diagonal indicate agreement across models for a given disease category, while off-diagonal cells indicate disagreement. Abbreviations: LLM, large language model.

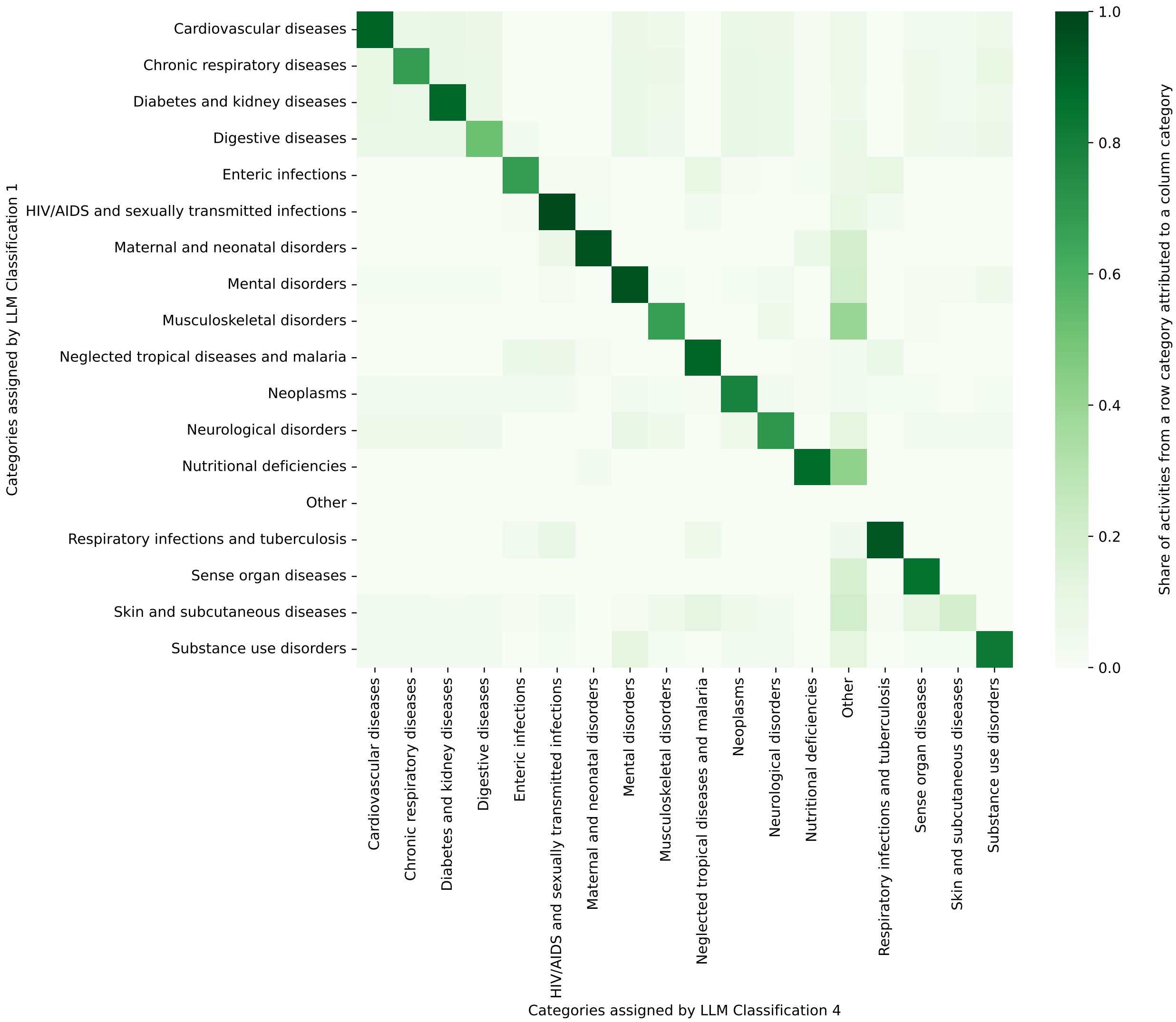

Fig. S21. Comparison between DeepSeek-V3.1 classifications and primary model classifications. The comparison shows how aid projects ("activities") are classified into disease categories by our machine learning pipeline using different LLMs. LLM Classification 1 uses Llama-3.1-70B-Instruct (rows), LLM Classification 4 uses DeepSeek-V3.1 (columns). Each cell reports the share of activities from a row category that is assigned to the corresponding column category, so rows sum to 1. Values on the diagonal indicate agreement across models for a given disease category, while off-diagonal cells indicate disagreement. Abbreviations: LLM, large language model.

S2.2 Robustness to chunk-based input processing

Additionally, we specifically addressed potential failure modes due to long project descriptions, negation, and short, non-English, and semantically ambiguous project descriptions. First, to assess

one alternative input-processing strategy for long descriptions, we implemented a chunk-based variant of the pipeline on the manual validation set. Specifically, we divided the textual inputs (project title, short description, long description) into four identically sized chunks, classified each chunk separately, and concatenated the chunks and classifications to a single text. This concatenated text was then classified using a tailored prompt (Supplementary Figure S22). The chunk-based classification did not yield a meaningful improvement relative to the original classification (overall accuracy of 0.72 and micro-averaged F_1-score of 0.83; Supplementary Tables S9 and S10). We interpret this analysis as a robustness check of one alternative input-processing implementation, not as a direct test of truncation or positional bias. The lower performance of the chunk-based variant may reflect limitations of the chunking design, loss of contextual information across chunks, or inadequacies of the tailored prompt. We therefore do not interpret this result as evidence that truncation-related or positional effects are absent.

S2.3 Robustness to explicit negation

Second, we constructed a negation-focused validation set to assess classification performance on projects with negated disease mentions. Using spaCy and NegEx and the 500 disease keywords from the keyword-based classifier, we scanned all projects previously classified as disease-related and flagged descriptions in which a disease keyword occurred in a negated context (e.g., "no tuberculosis", "not HIV", "excluding malaria"). This procedure identified 2,589 projects with at least one explicit negated disease mention. From these, we randomly sampled 500 projects, which were manually labeled and re-classified using a specialized negation-aware prompt that explicitly restricts labels to diseases positively targeted by the project (Supplementary Figure S23). We then compared the original and negation-aware LLM classifications against the manual labels (Supplementary Tables S13–S15). On this subset, the negation-aware prompt did not improve performance relative to the original prompt (accuracy: 0.74 versus 0.78; micro-averaged F_1-score: 0.83 versus 0.85). Because the subset was derived from a NegEx-based screen for explicit negation patterns,

the analysis is restricted to explicit negation cases detectable by that procedure and does not address implicit or more complex discourse-level negation elsewhere in the corpus. Accordingly, the 2,589 flagged projects (approximately 0.8% of disease-related projects in the full corpus; $n = 321{,}527$) should be interpreted as a lower-bound estimate of explicit negation patterns detectable by this procedure rather than as an estimate of the overall prevalence of negation-related cases.

While the above analysis gives some intuition about the robustness of our LLM pipeline, the lower performance of the negation-aware prompt should likewise be interpreted narrowly as evidence that this specific prompt formulation did not improve performance on this subset and may also reflect a prompt design that is too restrictive for some descriptions. Because the subset was constructed from projects flagged by a NegEx-based screen rather than drawn as a random sample of the full corpus, the analysis is not designed to assess false positives arising from missed or implicit negation elsewhere in the data and should therefore not be interpreted as evidence that negation is not a relevant source of classification error more broadly. In addition, although manual labeling provides a useful reference for this targeted evaluation, some residual uncertainty may remain for difficult negated cases. Finally, the size of the 500-project negation-focused subset may limit robust category-specific inference.

S2.4 Robustness to short and non-English descriptions

Third, we created an additional validation set of 2000 projects with non-English descriptions shorter than 100 characters, which were translated using Google Translate and manually annotated. We then evaluated the original pipeline on this short and non-English subset that may be potentially more ambiguous in the classification than the rest of the sample (Supplementary Tables S11 and S12), finding that overall performance remains comparable to the main validation set (overall accuracy of 0.92 and micro-averaged F_1-score of 0.92), with misclassifications concentrated in inherently ambiguous descriptions.

S2.5 Robustness to position of disease mention

Fourth, we assessed whether classification performance varied with the position of disease-related information within the project description. Using the first disease-related keyword match as a proxy for the position of the disease mention, we stratified validation set projects into those in which the first mention appeared in the first quartile of the text (Q1) and those in which it appeared later (Q2–Q4). Model performance remained high in both strata, but was slightly higher for earlier mentions (accuracy: 0.94 versus 0.87; micro-averaged F_1-score: 0.96 versus 0.90; Supplementary Table S16). These results indicate some performance heterogeneity by the position of the disease mention, but it may also reflect meaningful differences in project structure: when disease-related information appears earlier, it is more likely to correspond to the central focus of the project, whereas later mentions may more often occur in broader or more peripheral contexts. We therefore interpret this result as indicating robust performance when disease-related information appears later in the text, while also suggesting that earlier mentions may provide a stronger signal of disease-specific project content.

You are an expert health policy assistant. Your task is to identify which disease categories are positively targeted by this development aid project, based on its description.

Read the project description carefully. ONLY include disease categories that are explicitly targeted, supported, or addressed in the project. DO NOT include diseases that are:

- Mentioned as excluded

- Mentioned as not funded

- Mentioned only in background context

- Described as 'not a focus' or 'not covered'

You may assign one or more of the following 17 disease categories:

[HIV/AIDS and sexually transmitted infections, Respiratory infections and tuberculosis, Enteric infections, Neglected tropical diseases and malaria, Maternal and neonatal disorders, Nutritional deficiencies, Neoplasms, Cardiovascular diseases, Chronic respiratory diseases, Digestive diseases, Neurological disorders, Mental disorders, Substance use disorders, Diabetes and kidney diseases, Skin and subcutaneous diseases, Sense organ diseases, Musculoskeletal disorders]

Answer as a comma-separated list of disease categories. Use "Other" or "General Health" if no specific disease is addressed. Respond with the label(s) only, without additional commentary.

The project was split into 4 chunks which were classified independently. The project description is the concatenation of all 4 chunks as well as the chunk classifications.

Create a final classification based on the chunk classifications.

This is the description:

Fig. S22. Prompt design for the chunk-based classification.

Performance metric	Chunk-based Classification
Accuracy	0.72
Micro-averaged precision	0.76
Micro-averaged recall	0.91
Micro-averaged F_1-score	0.83
Macro-averaged precision	0.75
Macro-averaged recall	0.92
Macro-averaged F_1-score	0.82

Table S9: **Overall classification performance using chunk-based classification.**

Disease category	Precision	Recall	True positives
Cardiovascular diseases	0.81	0.94	47
Chronic respiratory diseases	0.69	0.92	24
Diabetes and kidney diseases	0.83	1.00	38
Digestive diseases	0.54	0.78	14
Enteric infections	0.69	0.78	49
HIV/AIDS and STIs	0.78	0.97	97
Maternal and neonatal disorders	0.64	0.93	68
Mental disorders	0.75	0.89	93
Musculoskeletal disorders	0.72	0.95	21
Neglected tropical diseases and malaria	0.91	0.99	143
Neoplasms	0.87	0.99	77
Neurological disorders	0.58	0.92	46
Nutritional deficiencies	0.73	0.94	134
Other	0.74	0.88	866
Respiratory infections and tuberculosis	0.87	0.97	175
Sense organ diseases	0.88	0.98	57
Skin and subcutaneous diseases	0.71	0.83	5
Substance use disorders	0.70	0.98	45

Table S10: **Classification performance by disease category using chunk-based classification.**

Performance metric	Our ML approach
Accuracy	0.92
Micro-averaged precision	0.95
Micro-averaged recall	0.90
Micro-averaged F_1-score	0.92
Macro-averaged precision	0.92
Macro-averaged recall	0.81
Macro-averaged F_1-score	0.85

Table S11: **Overall classification performance on short, ambiguous, and non-English project descriptions.** Abbreviations: ML, machine learning.

Disease category	Precision	Recall	True positives
Cardiovascular diseases	1.00	0.93	27
Chronic respiratory diseases	1.00	0.91	20
Diabetes and kidney diseases	1.00	1.00	18
Digestive diseases	1.00	0.58	11
Enteric infections	0.94	0.82	50
HIV/AIDS and STIs	0.99	0.98	371
Maternal and neonatal disorders	0.84	0.95	141
Mental disorders	0.94	0.94	94
Musculoskeletal disorders	0.92	0.44	12
Neglected tropical diseases and malaria	0.93	0.97	212
Neoplasms	0.97	0.75	33
Neurological disorders	0.83	0.70	19
Nutritional deficiencies	0.98	0.99	315
Other	1.00	0.91	485
Respiratory infections and tuberculosis	0.93	0.97	138
Sense organ diseases	0.98	0.80	59
Skin and subcutaneous diseases	0.50	0.11	2
Substance use disorders	0.93	0.89	41

Table S12: **Classification performance on short, ambiguous, and non-English project descriptions by disease category.**

You are an expert health policy assistant. Your task is to identify which disease categories are positively targeted by this development aid project, based on its description.

Read the project description carefully. ONLY include disease categories that are explicitly targeted, supported, or addressed in the project. DO NOT include diseases that are:

- Mentioned as excluded

- Mentioned as not funded

- Mentioned only in background context

- Described as 'not a focus' or 'not covered'

You may assign one or more of the following 17 disease categories:

[HIV/AIDS and sexually transmitted infections, Respiratory infections and tuberculosis, Enteric infections, Neglected tropical diseases and malaria, Maternal and neonatal disorders, Nutritional deficiencies, Neoplasms, Cardiovascular diseases, Chronic respiratory diseases, Digestive diseases, Neurological disorders, Mental disorders, Substance use disorders, Diabetes and kidney diseases, Skin and subcutaneous diseases, Sense organ diseases, Musculoskeletal disorders]

Answer as a comma-separated list of disease categories. Use "Other" or "General Health" if no specific disease is addressed. Respond with the label(s) only, without additional commentary.

Project Description:

"""

[concatenated title + descriptions]

"""

Fig. S23. Prompt design for the negation-aware classification.

Performance metric	Original Prompt	Optimized Prompt
Accuracy	0.78	0.74
Micro-averaged precision	0.89	0.80
Micro-averaged recall	0.81	0.88
Micro-averaged F_1-score	0.85	0.83
Macro-averaged precision	0.91	0.74
Macro-averaged recall	0.73	0.80
Macro-averaged F_1-score	0.78	0.74

Table S13: **Overall classification performance on project descriptions including negation.**

Disease category	Precision	Recall	True positives
Cardiovascular diseases	0.92	0.92	12
Chronic respiratory diseases	1.00	0.38	3
Diabetes and kidney diseases	1.00	0.95	18
Digestive diseases	0.00	0.00	0
Enteric infections	0.64	0.58	14
HIV/AIDS and STIs	0.95	0.97	317
Maternal and neonatal disorders	0.85	0.50	17
Mental disorders	1.00	0.50	10
Musculoskeletal disorders	0.00	0.00	0
Neglected tropical diseases and malaria	0.95	0.98	125
Neoplasms	0.97	0.95	35
Neurological disorders	0.90	0.90	9
Nutritional deficiencies	0.88	0.87	84
Other	0.00	0.00	0
Respiratory infections and tuberculosis	0.68	0.86	77
Sense organ diseases	0.00	0.00	0
Skin and subcutaneous diseases	1.00	0.50	1
Substance use disorders	1.00	0.38	3

Table S14: **Classification performance by disease category on project descriptions including negation using the original prompt.**

Disease category	Precision	Recall	True positives
Cardiovascular diseases	0.87	1.00	13
Chronic respiratory diseases	1.00	0.75	6
Diabetes and kidney diseases	0.86	0.95	18
Digestive diseases	0.00	0.00	0
Enteric infections	0.71	0.83	20
HIV/AIDS and STIs	0.91	1.00	326
Maternal and neonatal disorders	0.33	0.71	24
Mental disorders	0.56	0.75	15
Musculoskeletal disorders	0.00	0.00	0
Neglected tropical diseases and malaria	0.91	0.98	125
Neoplasms	0.88	1.00	37
Neurological disorders	0.75	0.90	9
Nutritional deficiencies	0.85	0.97	94
Other	0.33	0.03	2
Respiratory infections and tuberculosis	0.70	0.97	87
Sense organ diseases	1.00	0.33	1
Skin and subcutaneous diseases	0.67	1.00	2
Substance use disorders	0.45	0.62	5

Table S15: **Classification performance by disease category on project descriptions including negation using the negation-optimized prompt.**

Performance metric	Q1	Q2–Q4
Accuracy	0.94	0.87
Micro-averaged precision	0.98	0.96
Micro-averaged recall	0.93	0.85
Micro-averaged F_1-score	0.96	0.90
Macro-averaged precision	0.98	0.95
Macro-averaged recall	0.86	0.73
Macro-averaged F_1-score	0.91	0.80

Table S16: **Classification performance by position of disease mention.** Q1 denotes validation set projects in which the first disease-related keyword match appeared in the first quartile of the project description, whereas Q2–Q4 denote projects in which the first match appeared later in the text.

S3 Prompt design

The prompt was designed following best practices in prompt engineering and prior research [9–11]. It includes a short task description, a list of possible labels, and the description of the aid project to be classified. The prompt is designed to guide the LLM along the following reasoning process. (i) The LLM should first determine whether a project is health-related. If not (or if unclear), the LLM should classify the project as “Other”. (ii) If the project is health-related but broad and not related to a specific disease category, it should be classified as “General Health”. (iii) Only disease-specific projects are categorized into the 17 specific disease categories. To reduce false positives, we instructed the LLM to follow a conservative approach: when the classification was uncertain, it should default to “Other”. We found that this helps prevent the overestimation of funding for specific diseases by avoiding the misclassification of non-disease-related projects into disease categories. Similarly, we observed that introducing a category “General Health” helped improve classification performance. As a result, the LLM avoids forcing projects related to the broader health systems but that do not align with a specific disease into one of the narrow disease labels, which also minimized false positives. The full prompt is provided in Supplementary Figure S24.

Classify the given development aid project description by its primary objective:

1. If the primary focus is not on addressing diseases or health conditions, return 'Other' and stop. If the description is very ambiguous, return 'Other' and stop.
2. If the project is health-related, but addresses broad health initiatives or doesn't fit the provided specific disease categories perfectly, return 'General Health' and stop.
3. If disease is the main focus, evaluate if it fits into any of these specific disease categories: [HIV/AIDS and sexually transmitted infections, Respiratory infections and tuberculosis, Enteric infections, Neglected tropical diseases and malaria, Maternal and neonatal disorders, Nutritional deficiencies, Neoplasms, Cardiovascular diseases, Chronic respiratory diseases, Digestive diseases, Neurological disorders, Mental disorders, Substance use disorders, Diabetes and kidney diseases, Skin and subcutaneous diseases, Sense organ diseases, Musculoskeletal disorders]. Don't make assumptions if the text is not crystal clear. If you can't be sure, return 'General Health'.
4. If the project fits one or more specific categories, return those category names separated by commas.

Rules:

- Do not combine 'Other' or 'General Health' with disease categories.
- Only use labels from the provided list, 'Other', or 'General Health'.
- Respond with the label(s) only, without additional commentary.

Analyze this project description:

Fig. S24. Prompt design. The prompt combines a short task description, a list of possible labels, and the description of the ODA project to be classified. It guides the LLM along several reasoning steps to prevent the misclassification of non-disease-related projects or projects that do not align with a specific disease, effectively reducing false positives.

S4 Hyperparameters

The hyperparameters of our LLM approach were chosen as follows. We set the temperature and top-p values to 0.7 to balance consistency in the disease classification with sufficient flexibility for edge cases. We specified top-k = 50 and a repetition penalty of 1.0 to effectively distinguish between closely related disease categories while preventing classification loops in complex multi-disease descriptions. The maximum token output was capped at 40, which provided sufficient length for our classification task while ensuring computational scalability.

Donor name	Share of funding	Cumulative share of funding
United States	16.05%	16.05%
Germany	8.71%	24.76%
EU Institutions	8.5%	33.26%
Japan	6.59%	39.85%
United Kingdom	5.70%	45.55%
International Bank for Reconstruction and Development	5.35%	50.90%
Asian Development Bank	5.20%	56.10%
France	4.64%	60.74%
International Development Association	4.52%	65.26%
Inter-American Development Bank	4.33%	69.59%
Netherlands	2.31%	71.90%
Norway	1.81%	73.71%
Canada	1.77%	75.48%
Sweden	1.72%	77.20%
Australia	1.56%	78.76%
African Development Bank	1.36%	80.12%
Bill & Melinda Gates Foundation	1.33%	81.45%
Global Fund	1.22%	82.67%
Spain	1.21%	83.88%
African Development Fund	1.16%	85.04%
United Arab Emirates	1.07%	86.11%
Denmark	0.95%	87.06%
Turkey	0.95%	88.01%
Switzerland	0.93%	88.94%
Italy	0.87%	89.81%
Korea	0.76%	90.57%
Saudi Arabia	0.74%	91.31%
Global Alliance for Vaccines and Immunization	0.68%	91.99%
Asian Infrastructure Investment Bank	0.58%	92.57%
Belgium	0.52%	93.09%
UNICEF	0.47%	93.56%
European Bank for Reconstruction and Development	0.44%	94.00%
Austria	0.42%	94.42%
Finland	0.38%	94.80%
Ireland	0.35%	95.15%

Table S17: **Share and cumulative share of global development funding by donor from 2000 to 2022.** The top 35 donors of the 165 total donors contribute over 95% of the total funding.

Disease	ODA share (%)	95% UI
HIV/AIDS and sexually transmitted infections	33.87	[32.52, 35.30]
Respiratory infections and tuberculosis	23.91	[22.24, 25.57]
Nutritional deficiencies	12.21	[11.63, 12.78]
Neglected tropical diseases and malaria	11.55	[10.95, 12.15]
Maternal and neonatal disorders	10.67	[10.21, 11.14]
Enteric infections	5.28	[4.93, 5.65]
Mental disorders	0.74	[0.67, 0.82]
Neoplasms	0.47	[0.41, 0.53]
Substance use disorders	0.24	[0.21, 0.27]
Sense organ diseases	0.20	[0.18, 0.22]
Chronic respiratory diseases	0.20	[0.16, 0.24]
Cardiovascular diseases	0.18	[0.15, 0.21]
Diabetes and kidney diseases	0.17	[0.14, 0.21]
Musculoskeletal disorders	0.12	[0.10, 0.13]
Neurological disorders	0.11	[0.10, 0.12]
Digestive diseases	0.05	[0.04, 0.06]
Skin and subcutaneous diseases	0.04	[0.01, 0.07]

Table S18: **Disease-specific downstream ODA allocation estimates from the main classification.** Values are reported as the share of total ODA allocated to each disease category, together with bootstrap-based 95% uncertainty intervals (UIs).

S5 Keyword list

Classification Category	GBD Subcategories, Keywords and Reasoning

HIV/AIDS and sexually transmitted infections

A.1 HIV/AIDS and sexually transmitted infections

Keyword	Reasoning
Sexually transmitted infection	Direct descriptor of the subcategory
STI	Acronym for sexually transmitted infection
STIs	Plural acronym for sexually transmitted infections
STD	Acronym for sexually transmitted disease and alternative term for STI
STDs	Plural acronym for sexually transmitted diseases
Sexually transmitted disease	Alternative term for sexually transmitted infection
Safe sex	Practice to prevent transmission of STIs

A.1.1 HIV/AIDS

Keyword	Reasoning
HIV	Acronym for Human Immunodeficiency Virus - the cause of AIDS
AIDS	Acronym for Acquired Immunodeficiency Syndrome - caused by HIV
Acquired Immunodeficiency Syndrome	Full name of AIDS
Antiretroviral	Type of medication used to treat HIV/AIDS
Human Immunodeficiency Virus	Full name of HIV

A.1.2.1 Syphilis

Keyword	Reasoning
Syphilis	Name of the specific sexually transmitted infection
Treponema pallidum	Scientific name of the bacterium causing syphilis
Chancre	Primary symptom of syphilis infection

A.1.2.2 Chlamydial infection

Keyword	Reasoning
Chlamydial infection	Name of the specific sexually transmitted infection
Chlamydia	Common name for chlamydial infection

A.1.2.3 Gonococcal infection

Keyword	Reasoning
Gonococcal infection	Name of the specific sexually transmitted infection
Gonorrhea	Common name for gonococcal infection

A.1.2.4 Trichomoniasis

Keyword	Reasoning
Trichomoniasis	Name of the specific sexually transmitted infection
Trichomonas vaginalis	Scientific name of the parasite causing trichomoniasis

A.1.2.5 Genital herpes

Keyword	Reasoning
Genital herpes	Name of the specific sexually transmitted infection
Genital ulcer	Common symptom of genital herpes

Respiratory infections and tuberculosis

A.2.1 Tuberculosis

Keyword	Reasoning
Tuberculosis	Name of the specific respiratory infection
MDR-TB	Acronym for Multi-Drug Resistant Tuberculosis
XDR-TB	Acronym for Extensively Drug-Resistant Tuberculosis
Isoniazid	Antibiotic used to treat tuberculosis
Rifampicin	Antibiotic used to treat tuberculosis

Bacille Calmette-Guérin	Vaccine used to prevent tuberculosis
Pulmonary TB	Tuberculosis affecting the lungs
Extrapulmonary TB	Tuberculosis affecting areas outside the lungs
TB screening	Process of testing for tuberculosis
TB treatment	Medical care for tuberculosis patients
TB prevention	Measures to avoid tuberculosis transmission

A.2.1.1 Latent tuberculosis infection

Keyword	Reasoning
Latent TB	Tuberculosis infection without active symptoms
LTBI	Acronym for Latent Tuberculosis Infection
TB prophylaxis	Preventive treatment for latent tuberculosis

A.2.1.2 Drug-susceptible tuberculosis

Keyword	Reasoning
Drug-susceptible TB	Tuberculosis responsive to standard antibiotics

A.2.1.3 Multidrug-resistant tuberculosis without extensive drug resistance

Keyword	Reasoning
Drug-resistant TB	Tuberculosis not responsive to some standard antibiotics

A.2.2 Lower respiratory infections

Keyword	Reasoning
Bronchitis	Inflammation of the bronchial tubes
Pneumonia	Infection causing inflammation of the lungs
Influenza	Viral respiratory infection
Flu	Common name for influenza
Lung infection	General term for infections affecting the lungs

A.2.3 Upper respiratory infections

Keyword	Reasoning
Sinusitis	Inflammation of the sinuses
Pharyngitis	Inflammation of the pharynx (sore throat)
Laryngitis	Inflammation of the larynx (voice box)
Tonsillitis	Inflammation of the tonsils
Rhinitis	Inflammation of the nasal passages

A.2.4 Otitis media

Keyword	Reasoning
Otitis media	Infection of the middle ear

A.2.5 COVID-19

Keyword	Reasoning
COVID-19	Disease caused by the SARS-CoV-2 virus
Corona virus	General term for a family of viruses including SARS-CoV-2
Covid	Common abbreviation for COVID-19
Coronavirus	Another term for the virus causing COVID-19
SARS-CoV-2	Scientific name of the virus causing COVID-19

Enteric infections

A.3 Enteric infections

Keyword	Reasoning
Enteric infections	Infections affecting the intestines
Gastroenteritis	Inflammation of the stomach and intestines
Dysentery	Intestinal inflammation causing severe diarrhea
Cholera	Severe diarrheal disease caused by Vibrio cholerae

Rotavirus	Common cause of severe diarrhea in children
Norovirus	Common cause of viral gastroenteritis

A.3.1 Diarrheal diseases

Keyword	Reasoning
Diarrhea	Main symptom of many enteric infections
Diarrhoeal	Relating to diarrhea
Diarrhoeal	Relating to diarrhea
Oral rehydration therapy	Treatment to prevent dehydration in diarrheal diseases

A.3.2 Typhoid and paratyphoid

Keyword	Reasoning
Typhoid	Bacterial infection caused by Salmonella Typhi
Paratyphoid	Similar to typhoid but caused by Salmonella Paratyphi
Salmonella Typhi	Bacterium causing typhoid fever
Salmonella Paratyphi	Bacterium causing paratyphoid fever

A.3.3 Invasive Non-typhoidal Salmonella (iNTS)

Keyword	Reasoning
iNTS	Acronym for Invasive Non-typhoidal Salmonella

A.3.5 Other intestinal infectious diseases

Keyword	Reasoning
Foodborne illness	Infections transmitted through contaminated food
Waterborne disease	Infections transmitted through contaminated water
Giardiasis	Intestinal infection caused by Giardia parasite
Cryptosporidiosis	Intestinal infection caused by Cryptosporidium parasite
Amoebiasis	Intestinal infection caused by Entamoeba histolytica
Campylobacteriosis	Intestinal infection caused by Campylobacter bacteria
Shigellosis	Intestinal infection caused by Shigella bacteria
E. coli	Common cause of intestinal infections
Clostridium difficile	Bacterium causing antibiotic-associated diarrhea
Helminthiasis	Intestinal worm infections
Vibrio cholerae	Bacterium causing cholera
Salmonella	Genus of bacteria causing various intestinal infections
Intestinal infectious disease	General term for infections of the intestines
Fecal-oral route	Common transmission method for intestinal infections
Enteric	Relating to the intestines
Diarrhoeal	Relating to diarrhea
Diarrhoeal	Relating to diarrhea
Bacterial enteritis	Intestinal inflammation caused by bacteria
Intestinal infection	General term for infections in the intestines

Neglected tropical diseases and malaria

A.4 Neglected tropical diseases and malaria

Keyword	Reasoning
Neglected tropical disease	Diverse group of tropical infections in impoverished communities

A.4.1 Malaria

Keyword	Reasoning
Malaria	Mosquito-borne parasitic disease
Tropical parasitic disease	Category of diseases including malaria
Vector-borne disease	Diseases transmitted by insects or other animals
Plasmodium	Genus of parasites causing malaria
P. falciparum	Most dangerous species of malaria parasite
P. vivax	Common species of malaria parasite

Antimalarial	Medications used to treat or prevent malaria
Mosquito nets	Prevention method for malaria
Indoor residual spraying	Insecticide application to prevent malaria transmission
Rapid diagnostic test	Quick test to diagnose malaria
Artemisinin-based combination therapy	Standard treatment for malaria

A.4.2 Chagas disease

Keyword	Reasoning
Chagas disease	Parasitic disease common in Latin America
Chagas	Short form of Chagas disease
Trypanosoma cruzi	Parasite causing Chagas disease
American trypanosomiasis	Alternative name for Chagas disease
Kissing bug disease	Common name for Chagas disease

A.4.3 Leishmaniasis

Keyword	Reasoning
Leishmaniasis	Parasitic disease transmitted by sandflies
Leishmania	Genus of parasites causing leishmaniasis
Sandfly fever	Alternative name for leishmaniasis

A.4.3.1 Visceral leishmaniasis

Keyword	Reasoning
Kala-azar	Common name for visceral leishmaniasis
Black fever	Another common name for visceral leishmaniasis

A.4.3.2 Cutaneous and mucocutaneous leishmaniasis

Keyword	Reasoning
Skin sores	Symptom of cutaneous leishmaniasis
Espundia	South American term for mucocutaneous leishmaniasis

A.4.4 African trypanosomiasis

Keyword	Reasoning
African trypanosomiasis	Parasitic disease transmitted by tsetse flies
Sleeping sickness	Common name for African trypanosomiasis
Tsetse fly disease	Alternative name for African trypanosomiasis
Trypanosoma brucei	Parasite causing African trypanosomiasis

A.4.5 Schistosomiasis

Keyword	Reasoning
Schistosomiasis	Parasitic disease caused by flatworms
Bilharzia	Alternative name for schistosomiasis
Snail fever	Common name for schistosomiasis
Schistosoma	Genus of parasites causing schistosomiasis

A.4.6 Cysticercosis

Keyword	Reasoning
Cysticercosis	Tissue infection caused by tapeworm larvae
Neurocysticercosis	Cysticercosis affecting the brain
Taenia solium	Tapeworm species causing cysticercosis

A.4.7 Cystic echinococcosis

Keyword	Reasoning
Echinococcosis	Parasitic disease caused by tapeworm larvae
Hydatid disease	Alternative name for echinococcosis

A.4.8 Lymphatic filariasis

Keyword	Reasoning
Lymphatic filariasis	Parasitic disease affecting the lymphatic system

Wuchereria bancrofti	Main parasite causing lymphatic filariasis
Brugia malayi	Another parasite causing lymphatic filariasis

A.4.9 Onchocerciasis

Keyword	Reasoning
Onchocerciasis	Parasitic disease causing skin and eye problems
River blindness	Common name for onchocerciasis
Onchocerca volvulus	Parasite causing onchocerciasis

A.4.10 Trachoma

Keyword	Reasoning
Trachoma	Bacterial eye infection causing blindness
Chlamydia trachomatis	Bacterium causing trachoma
Trichiasis	Complication of trachoma affecting eyelashes

A.4.11 Dengue

Keyword	Reasoning
Dengue	Mosquito-borne viral disease
Aedes mosquito	Vector for dengue virus

A.4.12 Yellow fever

Keyword	Reasoning
Yellow fever	Mosquito-borne viral disease
YF vaccine	Vaccine to prevent yellow fever
Aedes aegypti	Mosquito species transmitting yellow fever

A.4.13 Rabies

Keyword	Reasoning
Rabies	Viral disease affecting the nervous system
Post-exposure prophylaxis	Preventive treatment after potential rabies exposure
Lyssavirus	Genus of viruses causing rabies

A.4.14 Intestinal nematode infections

Keyword	Reasoning
Intestinal nematode infections	Infections caused by intestinal worms
Soil-transmitted helminths	Common name for intestinal worms
Intestinal worms	General term for parasitic worms in the intestines

A.4.14.1 Ascariasis

Keyword	Reasoning
Ascariasis	Intestinal infection caused by Ascaris roundworms
Roundworm	Common name for Ascaris worms
Ascaris lumbricoides	Scientific name of the roundworm causing ascariasis

A.4.14.2 Trichuriasis

Keyword	Reasoning
Trichuriasis	Intestinal infection caused by whipworms
Whipworm	Common name for Trichuris worms
Trichuris trichiura	Scientific name of the whipworm causing trichuriasis

A.4.14.3 Hookworm disease

Keyword	Reasoning
Hookworm disease	Intestinal infection caused by hookworms
Ancylostomiasis	Alternative name for hookworm disease
Necator americanus	Species of hookworm causing infection
Ancylostoma duodenale	Another species of hookworm causing infection

A.4.15 Food-borne trematodiases

Keyword	Reasoning
Food-borne trematodiases	Parasitic infections transmitted through food
Liver flukes	Parasites affecting the liver
Lung flukes	Parasites affecting the lungs
Intestinal flukes	Parasites affecting the intestines

A.4.16 Leprosy

Keyword	Reasoning
Leprosy	Chronic bacterial infection affecting skin and nerves
Hansen's disease	Alternative name for leprosy
Mycobacterium leprae	Bacterium causing leprosy
Hansens disease	Alternative spelling of Hansen's disease

A.4.17 Ebola

Keyword	Reasoning
Ebola	Severe viral hemorrhagic fever
Filovirus	Family of viruses including Ebola

A.4.18 Zika virus

Keyword	Reasoning
Zika virus	Mosquito-borne virus causing birth defects
Zika	Short form of Zika virus

A.4.19 Guinea worm disease

Keyword	Reasoning
Guinea worm disease	Parasitic infection caused by Dracunculus worm
Guinea worm	Common name for the parasite causing the disease
Dracunculiasis	Scientific name for Guinea worm disease
Dracunculus medinensis	Scientific name of the Guinea worm

A.4.20 Other neglected tropical diseases

Keyword	Reasoning
Buruli ulcer	Bacterial infection causing skin ulcers
Yaws	Bacterial infection affecting skin bones and cartilage
Mycetoma	Chronic skin and soft tissue infection
Chromoblastomycosis	Fungal skin infection
Deep mycoses	Fungal infections affecting deep tissues

Maternal and neonatal disorders

A.6.1 Maternal disorders

Maternal disorder	Health problems related to pregnancy and childbirth
Maternal disease	Diseases affecting pregnant women
Maternal infections	Infections affecting pregnant women
Miscarriage	Spontaneous loss of pregnancy before 20 weeks
Ectopic pregnancy	Pregnancy outside the uterus
Maternal deaths	Deaths related to pregnancy or childbirth
Eclampsia	Seizures during pregnancy due to high blood pressure
Pre-eclampsia	High blood pressure during pregnancy

A.6.1.1 Maternal hemorrhage

Keyword	Reasoning
Maternal hemorrhage	Excessive bleeding during pregnancy or childbirth

A.6.1.2 Maternal sepsis and other maternal infections

Keyword	Reasoning
Maternal sepsis	Infection during pregnancy or after childbirth

A.6.1.4 Maternal obstructed labor and uterine rupture

Keyword	Reasoning
Maternal obstructed labor	Difficulty in fetus passing through the birth canal
Maternal uterine rupture	Tearing of the uterus during pregnancy or childbirth

A.6.1.5 Maternal abortion and miscarriage

Keyword	Reasoning
Maternal abortion	Termination of pregnancy

A.6.2 Neonatal disorders

Keyword	Reasoning
Neonatal disorder	Health problems affecting newborns
Neonatal disease	Diseases affecting newborns
Birth asphyxia	Lack of oxygen to a baby during birth
Stillbirth	Baby born without signs of life at or after 28 weeks
Neonatal encephalopathy	Brain disorder in newborns
Neonatal infections	Infections affecting newborns

A.6.2.1 Neonatal preterm birth

Keyword	Reasoning
Preterm	Born before 37 weeks of pregnancy
Neonatal preterm birth	Birth before 37 weeks of pregnancy

A.6.2.3 Neonatal sepsis and other neonatal infections

Keyword	Reasoning
Neonatal sepsis	Blood infection in newborns
Neonatal jaundice	Yellowing of a newborn's skin and eyes

A.6.2.4 Hemolytic disease and other neonatal jaundice

Keyword	Reasoning
Hemolytic disease	Blood disorder in newborns

Nutritional Deficiencies

A.7 Nutritional deficiencies

Keyword	Reasoning
Malnutrition	Lack of proper nutrition
Undernutrition	Insufficient intake of nutrients
Stunting	Impaired growth and development due to poor nutrition
Nutritional deficiency	Lack of essential nutrients
Nutritional deficiencies	Multiple lacks of essential nutrients

A.7.1 Protein-energy malnutrition

Keyword	Reasoning
Protein-energy deficiency	Lack of protein and energy in the diet
Protein-energy deficiencies	Multiple lacks of protein and energy in the diet
Wasting	Severe weight loss due to malnutrition

A.7.2 Iodine deficiency

Keyword	Reasoning
Iodine deficiency	Lack of iodine in the diet
Iodine deficiencies	Multiple instances of iodine lack in the diet

A.7.3 Vitamin A deficiency

Keyword	Reasoning
Vitamin A deficiencies	Multiple instances of vitamin A lack in the diet

Vitamin A deficiency	Lack of vitamin A in the diet

A.7.4 Dietary iron deficiency

Keyword	Reasoning
Iron deficiencies	Multiple instances of iron lack in the diet
Iron deficiency	Lack of iron in the diet
Anemia	Condition often caused by iron deficiency

A.7.5 Other nutritional deficiencies

Keyword	Reasoning
Vitamin deficiency	Lack of essential vitamins in the diet
Zinc deficiency	Lack of zinc in the diet
Folate deficiency	Lack of folate (vitamin B9) in the diet

Neoplasms

B.1 Neoplasms

Keyword	Reasoning
Neoplasm	Abnormal growth of tissue (tumor)
Tumor	Abnormal mass of tissue
Malignant	Cancerous
Carcinoma	Cancer originating in epithelial cells
Sarcoma	Cancer originating in connective tissue
Leukemia	Cancer of blood-forming tissues
Lymphoma	Cancer of lymphatic system
Melanoma	Cancer originating in melanocytes
Metastasis	Spread of cancer from one part of the body to another
Oncology	Study and treatment of cancer
Cancer	General term for malignant neoplasms

B.1.7.5 Hepatoblastoma

Keyword	Reasoning
Hepatoblastoma	Rare liver cancer typically occurring in young children

B.1.25.1 Retinoblastoma

Keyword	Reasoning
Retinoblastoma	Cancer of the retina typically occurring in young children

B.1.28 Mesothelioma

Keyword	Reasoning
Mesothelioma	Cancer affecting the lining of the lungs and abdomen

B.1.31 Multiple myeloma

Keyword	Reasoning
Multiple myeloma	Cancer of plasma cells

Cardiovascular diseases

B.2 Cardiovascular diseases

Keyword	Reasoning
Cardiovascular disease	Diseases affecting the heart and blood vessels
Heart disease	Diseases affecting the heart
Hypertension	High blood pressure
High blood pressure	Another term for hypertension
Coronary artery disease	Narrowing of the coronary arteries
Atherosclerosis	Buildup of plaque in the arteries

Arrhythmia	Irregular heartbeat
Atrial fibrillation	Common type of arrhythmia
Heart failure	Heart's inability to pump blood effectively
Myocardial infarction	Heart attack
Aneurysm	Bulge in a blood vessel wall
Peripheral artery disease	Narrowing of arteries in the limbs
Thrombosis	Blood clot formation

B.2.3 Stroke

Keyword	Reasoning
Stroke	Sudden interruption of blood supply to the brain

B.2.3.2 Intracerebral hemorrhage

Keyword	Reasoning
Intracerebral hemorrhage	Bleeding within the brain tissue

B.2.3.3 Subarachnoid hemorrhage

Keyword	Reasoning
Subarachnoid hemorrhage	Bleeding in the space surrounding the brain

B.2.5 Non-rheumatic valvular heart disease

Keyword	Reasoning
Valve disease	Diseases affecting heart valves

B.2.6 Cardiomyopathy and myocarditis

Keyword	Reasoning
Cardiomyopathy	Diseases of the heart muscle

B.2.6.1 Myocarditis

Keyword	Reasoning
Myocarditis	Inflammation of the heart muscle

B.2.8 Atrial fibrillation and flutter

Keyword	Reasoning
Atrial fibrillation	Common type of irregular heartbeat

B.2.9 Aortic aneurysm

Keyword	Reasoning
Aortic aneurysm	Bulge in the wall of the aorta

B.2.10 Lower extremity peripheral arterial disease

Keyword	Reasoning
Peripheral arterial disease	Narrowing of arteries in the legs

B.2.11 Endocarditis

Keyword	Reasoning
Endocarditis	Inflammation of the inner lining of the heart

B.2.12 Other cardiovascular and circulatory diseases

Keyword	Reasoning
Circulatory disease	Diseases affecting blood circulation

Chronic respiratory diseases

B.3 Chronic respiratory diseases

Keyword	Reasoning
Chronic respiratory disease	Long-term diseases affecting the airways and lungs

B.3.1 Chronic obstructive pulmonary disease

Keyword	Reasoning
COPD	Acronym for Chronic Obstructive Pulmonary Disease
Chronic obstructive pulmonary disease	Group of lung diseases causing airflow blockage

B.3.2 Pneumoconiosis

Keyword	Reasoning
Pneumoconiosis	Lung diseases caused by inhalation of dust

B.3.2.1 Silicosis

Keyword	Reasoning
Silicosis	Lung disease caused by inhalation of silica dust

B.3.2.2 Asbestosis

Keyword	Reasoning
Asbestosis	Lung disease caused by inhalation of asbestos fibers

B.3.3 Asthma

Keyword	Reasoning
Asthma	Chronic disease causing airway inflammation and narrowing

B.3.4 Interstitial lung disease and pulmonary sarcoidosis

Keyword	Reasoning
Pulmonary fibrosis	Scarring of lung tissue
Sarcoidosis	Inflammatory disease affecting multiple organs including lungs

B.3.5 Other chronic respiratory diseases

Keyword	Reasoning
Emphysema	Damage to the air sacs in the lungs
Cystic fibrosis	Inherited disorder affecting lungs and other organs
Bronchitis	Inflammation of the bronchial tubes

Digestive diseases

B.4 Digestive diseases

Keyword	Reasoning
Digestive disease	Diseases affecting the digestive system
Digestive system disease	Another term for digestive diseases

B.4.1 Cirrhosis and other chronic liver diseases

Keyword	Reasoning
Cirrhosis	Scarring of the liver
Chronic liver disease	Long-term diseases affecting the liver
Fatty liver disease	Accumulation of fat in the liver

B.4.2.1 Peptic ulcer disease

Keyword	Reasoning
Peptic ulcer disease	Sores in the lining of stomach or small intestine

B.4.2.2 Gastritis and duodenitis

Keyword	Reasoning
Gastritis	Inflammation of the stomach lining
Duodenitis	Inflammation of the duodenum

B.4.2.3 Gastroesophageal reflux disease

Keyword	Reasoning

GERD	Acronym for Gastroesophageal Reflux Disease
Gastroesophageal reflux disease	Chronic digestive disease causing acid reflux

B.4.3 Appendicitis

Keyword	Reasoning
Appendicitis	Inflammation of the appendix

B.4.4 Paralytic ileus and intestinal obstruction

Keyword	Reasoning
Paralytic ileus	Intestinal paralysis
Intestinal obstruction	Blockage of the intestines

B.4.5 Inguinal, femoral, and abdominal hernia

Keyword	Reasoning
Inguinal hernia	Protrusion of tissue through the groin
Femoral hernia	Protrusion of tissue through the upper thigh
Abdominal hernia	Protrusion of tissue through the abdominal wall

B.4.6 Inflammatory bowel disease

Keyword	Reasoning
Inflammatory bowel disease	Chronic inflammation of the digestive tract
Crohn's disease	Type of inflammatory bowel disease
Ulcerative colitis	Type of inflammatory bowel disease

B.4.7 Vascular intestinal disorders

Keyword	Reasoning
Vascular intestinal disorder	Disorders affecting blood vessels in the intestines

B.4.8 Gallbladder and biliary diseases

Keyword	Reasoning
Gallbladder	Organ storing and concentrating bile
Biliary disease	Diseases affecting the biliary system

B.4.9 Pancreatitis

Keyword	Reasoning
Pancreatitis	Inflammation of the pancreas

B.4.10 Other digestive diseases

Keyword	Reasoning
Gallstone	Solid material that forms in the gallbladder
Irritable bowel syndrome	Disorder affecting the large intestine
Bowel disease	Diseases affecting the bowels
Intestinal disorder	Disorders affecting the intestines
Intestinal disease	Diseases affecting the intestines

Neurological disorders

B.5 Neurological disorders

Keyword	Reasoning
Neurological disorder	Disorders affecting the nervous system

B.5.1 Alzheimer's disease and other dementias

Keyword	Reasoning
Alzheimer	Common form of dementia
Dementia	Decline in cognitive function

B.5.2 Parkinson's disease

Keyword	Reasoning
Parkinson	Progressive nervous system disorder affecting movement

B.5.3 Idiopathic epilepsy

Keyword	Reasoning
Epilepsy	Neurological disorder causing seizures
Seizure	Sudden uncontrolled electrical disturbance in the brain

B.5.4 Multiple sclerosis

Keyword	Reasoning
Multiple sclerosis	Disease affecting the central nervous system

B.5.5 Motor neuron disease

Keyword	Reasoning
Motor neuron disease	Progressive neurological disease
ALS	Amyotrophic Lateral Sclerosis a type of motor neuron disease

B.5.6 Headache disorders

Keyword	Reasoning
Headache disorder	Disorders characterized by recurrent headaches

B.5.6.1 Migraine

Keyword	Reasoning
Migraine	Severe recurring headache

B.5.6.2 Tension-type headache

Keyword	Reasoning
Tension-type headache	Common form of primary headache

B.5.7 Other neurological disorders

Keyword	Reasoning
Neuropathy	Damage or dysfunction of nerves
Cerebral palsy	Group of disorders affecting movement and posture
Huntington's disease	Inherited disorder causing progressive brain damage

Mental disorders

B.6 Mental disorders

Keyword	Reasoning
Mental disorder	Disorders affecting behavior and thinking

B.6.1 Schizophrenia

Keyword	Reasoning
Schizophrenia	Severe mental disorder affecting thinking and behavior

B.6.2 Depressive disorders

Keyword	Reasoning
Depressive disorder	Mood disorders characterized by persistent feelings of sadness
Depression	Mood disorder characterized by persistent sadness

B.6.2.2 Dysthymia

Keyword	Reasoning
Dysthymia	Persistent mild depression

B.6.3 Bipolar disorder

Keyword	Reasoning
Bipolar	Mood disorder with alternating episodes of mania and depression

B.6.4 Anxiety disorders

Keyword	Reasoning
Anxiety	Disorders characterized by excessive worry or fear

B.6.5 Eating disorders

Keyword	Reasoning
Eating disorder	Disorders characterized by abnormal eating habits

B.6.5.1 Anorexia nervosa

Keyword	Reasoning
Anorexia	Eating disorder characterized by low weight and food restriction

B.6.5.2 Bulimia nervosa

Keyword	Reasoning
Bulimia	Eating disorder characterized by binge eating and purging

B.6.6 Autism spectrum disorders

Keyword	Reasoning
Autism	Developmental disorder affecting communication and behavior

B.6.7 Attention-deficit/hyperactivity disorder

Keyword	Reasoning
Attention-deficit/hyperactivity disorder	Neurodevelopmental disorder affecting attention and behavior
ADHD	Acronym for Attention-Deficit/Hyperactivity Disorder

B.6.8 Conduct disorder

Keyword	Reasoning
Conduct disorder	Behavioral disorder in children and adolescents

B.6.10 Other mental disorders

Keyword	Reasoning
PTSD	Post-Traumatic Stress Disorder
OCD	Obsessive-Compulsive Disorder
Personality disorder	Disorders characterized by unhealthy patterns of thinking and behavior
Post-traumatic stress	Disorder that may develop after experiencing a traumatic event

Substance use disorders

B.7 Substance use disorders

Keyword	Reasoning
Substance use disorder	Disorders related to the use of psychoactive substances
Substance abuse	Harmful use of psychoactive substances
Substance dependence	Physical or psychological dependence on a substance

B.7.1 Alcohol use disorders

Keyword	Reasoning
Alcohol use disorder	Disorder characterized by problematic patterns of alcohol use
Alcohol abuse	Harmful or hazardous use of alcohol
Alcoholism	Addiction to alcohol

B.7.2 Drug use disorders

Keyword	Reasoning
Drug use disorder	Disorder characterized by problematic patterns of drug use
Drug abuse	Harmful or hazardous use of drugs

B.7.2.1 Opioid use disorders

Keyword	Reasoning
Opioid use disorder	Disorder related to the use of opioids
Opioid abuse	Harmful or hazardous use of opioids

B.7.2.2 Cocaine use disorders

Keyword	Reasoning
Cocaine use disorder	Disorder related to the use of cocaine
Cocaine abuse	Harmful or hazardous use of cocaine

B.7.2.3 Amphetamine use disorders

Keyword	Reasoning
Amphetamine use disorder	Disorder related to the use of amphetamines
Amphetamine abuse	Harmful or hazardous use of amphetamines

B.7.2.4 Cannabis use disorders

Keyword	Reasoning
Cannabis use disorder	Disorder related to the use of cannabis
Cannabis abuse	Harmful or hazardous use of cannabis

B.7.2.5 Other drug use disorders

Keyword	Reasoning
Substance misuse	Inappropriate use of psychoactive substances

Diabetes and kidney diseases

B.8.1 Diabetes mellitus

Keyword	Reasoning
Diabetes	Group of metabolic disorders characterized by high blood sugar
Diabetic	Relating to diabetes
Insulin resistance	Condition in which cells don't respond normally to insulin

B.8.1.1 Diabetes mellitus type 1

Keyword	Reasoning
Diabetes mellitus type 1	Autoimmune form of diabetes

B.8.1.2 Diabetes mellitus type 2

Keyword	Reasoning
Diabetes mellitus type 2	Most common form of diabetes often related to lifestyle

B.8.2 Chronic kidney disease

Keyword	Reasoning
Kidney disease	Long-term condition where kidneys don't work properly
Nephropathy	Damage to or disease of the kidneys
Dialysis	Treatment for kidney failure
Renal failure	Condition where kidneys fail to function properly
Chronic kidney disease	Long-term condition of kidney damage or dysfunction

B.8.2.4 Chronic kidney disease due to glomerulonephritis

Keyword	Reasoning
Glomerulonephritis	Inflammation of the kidney's filtering units

B.8.2.5 Chronic kidney disease due to other and unspecified causes

Keyword	Reasoning
End-stage renal disease	Final stage of chronic kidney disease

B.8.3 Acute glomerulonephritis

Keyword	Reasoning

Acute kidney injury	Sudden decrease in kidney function

Skin and subcutaneous diseases

B.9 Skin and subcutaneous diseases

Keyword	Reasoning
Skin disease	Diseases affecting the skin
Subcutaneous disease	Diseases affecting the tissue beneath the skin

B.9.1 Dermatitis

Keyword	Reasoning
Dermatitis	Inflammation of the skin

B.9.1.1 Atopic dermatitis

Keyword	Reasoning
Eczema	Inflammatory skin condition causing itchy rashes

B.9.2 Psoriasis

Keyword	Reasoning
Psoriasis	Chronic autoimmune condition causing rapid skin cell growth

B.9.3 Bacterial skin diseases

Keyword	Reasoning
Bacterial skin disease	Skin infections caused by bacteria

B.9.3.1 Cellulitis

Keyword	Reasoning
Cellulitis	Bacterial infection of the deep layers of skin

B.9.3.2 Pyoderma

Keyword	Reasoning
Pyoderma	Bacterial skin infection characterized by pus-filled lesions

B.9.4 Scabies

Keyword	Reasoning
Scabies	Skin infestation caused by tiny mites

B.9.7 Acne vulgaris

Keyword	Reasoning
Acne	Common skin condition causing pimples and other lesions
Acne vulgaris	Medical term for common acne

B.9.8 Alopecia areata

Keyword	Reasoning
Alopecia	General term for hair loss
Alopecia areata	Autoimmune disorder causing hair loss in patches

B.9.9 Pruritus

Keyword	Reasoning
Pruritus	Itching sensation

B.9.10 Urticaria

Keyword	Reasoning
Urticaria	Hives condition causing itchy welts

B.9.11 Decubitus ulcer

Keyword	Reasoning

Decubitus ulcer	Pressure sores or bedsores

B.9.12 Other skin and subcutaneous diseases

Keyword	Reasoning
Rosacea	Chronic inflammatory skin condition
Vitiligo	Condition causing loss of skin pigmentation

Sense organ diseases

B.10 Sense organ diseases

Keyword	Reasoning
Sense organ disease	Diseases affecting organs of sight hearing smell taste or touch

B.10.1 Blindness and vision loss

Keyword	Reasoning
Blindness	Complete or partial loss of sight
Vision loss	Partial or complete loss of vision
Vision impairment	Decreased ability to see
Visual impairment	Another term for vision impairment

B.10.1.1 Glaucoma

Keyword	Reasoning
Glaucoma	Group of eye conditions damaging the optic nerve

B.10.1.2 Cataract

Keyword	Reasoning
Cataract	Clouding of the eye's natural lens

B.10.1.3 Age-related macular degeneration

Keyword	Reasoning
Macular degeneration	Eye disorder causing loss of central vision

B.10.1.4 Refraction disorders

Keyword	Reasoning
Refraction disorder	Vision problems due to how the eye focuses light
Refractive error	Another term for refraction disorder

B.10.1.6 Other vision loss

Keyword	Reasoning
Retinopathy	Damage to the retina of the eye
Amblyopia	Decreased vision in one eye also known as lazy eye

B.10.2 Age-related and other hearing loss

Keyword	Reasoning
Hearing loss	Partial or complete inability to hear
Tinnitus	Perception of noise or ringing in the ears
Deafness	Severe or complete hearing loss

B.10.3 Other sense organ diseases

Keyword	Reasoning
Anosmia	Loss of sense of smell
Ageusia	Loss of sense of taste
Conjunctivitis	Inflammation of the conjunctiva of the eye
Vestibular disorder	Disorders affecting balance and spatial orientation

Musculoskeletal disorders

B.11 Musculoskeletal disorders

Keyword	Reasoning
Musculoskeletal disorder	Disorders affecting muscles bones and joints
Musculoskeletal disease	Diseases affecting muscles bones and joints

B.11.1 Rheumatoid arthritis

Keyword	Reasoning
Rheumatoid arthritis	Chronic inflammatory disorder affecting joints

B.11.2 Osteoarthritis

Keyword	Reasoning
Osteoarthritis	Degenerative joint disease
Arthritis	General term for joint inflammation

B.11.3 Low back pain

Keyword	Reasoning
Low back pain	Pain in the lower back

B.11.4 Neck pain

Keyword	Reasoning
Neck pain	Pain in the neck area

B.11.5 Gout

Keyword	Reasoning
Gout	Form of inflammatory arthritis caused by uric acid crystals

B.11.6 Other musculoskeletal disorders

Keyword	Reasoning
Tendinitis	Inflammation of a tendon
Scoliosis	Abnormal curvature of the spine
Carpal tunnel syndrome	Compression of the median nerve in the wrist
Osteoporosis	Condition causing bones to become weak and brittle
Fibromyalgia	Chronic condition causing widespread muscle pain and tenderness
Bursitis	Inflammation of the fluid-filled sacs that cushion joints
Spinal stenosis	Narrowing of spaces in the spine
Rotator cuff disorder	Injuries and conditions affecting the shoulder joint
Muscular dystrophy	Group of genetic diseases causing progressive muscle weakness
Bone fractures	Breaks in bone continuity

Supplementary References

[1] Murray, C. J. L. & Lopez, A. D. *The Global Burden of Disease: A comprehensive assessment of mortality and disability from diseases, injuries, and risk factors in 1990 and projected to 2020* (Harvard School of Public Health, Cambridge, MA, 1996).

[2] James, S. L. *et al.* Global, regional, and national incidence, prevalence, and years lived with disability for 354 diseases and injuries for 195 countries and territories, 1990–2017: a systematic analysis for the Global Burden of Disease Study 2017. *The Lancet* **392**, 1789–1858 (2018).

[3] Pitt, C. *et al.* Countdown to 2015: an analysis of donor funding for prenatal and neonatal health, 2003–2013. *BMJ Global Health* **2**, e000205 (2017). Accessed June 1, 2025.

[4] U.S. National Library of Medicine. Medical subject headings. URL https://www.nlm.nih.gov/mesh/meshhome.html. Accessed June 1, 2025.

[5] OpenAI. Introducing GPT-4.1 in the API (2025). URL https://openai.com/index/gpt-4-1/. Accessed March 1, 2026.

[6] OpenAI. GPT-4.1 mini model | OpenAI API (2025). URL https://developers.openai.com/api/docs/models/gpt-4.1-mini. Accessed March 1, 2026.

[7] DeepSeek-AI *et al.* DeepSeek-V3 technical report (2025). URL https://arxiv.org/abs/2412.19437. Accessed March 1, 2026.

[8] Sandmann, S. *et al.* Benchmark evaluation of DeepSeek large language models in clinical decision-making. *Nature Medicine* **31**, 2546–2549 (2025).

[9] Feuerriegel, S. *et al.* Using natural language processing to analyse text data in behavioural science. *Nature Reviews Psychology* **4**, 96–111 (2025).

[10] Giray, L. Prompt engineering with ChatGPT: a guide for academic writers. *Annals of Biomedical Engineering* **51**, 2629–2633 (2023).

[11] Lin, Z. How to write effective prompts for large language models. *Nature Human Behaviour* **8**, 611–615 (2024).
