# Supplementary material for: Tracking funding disparities in global health aid with machine learning
Source: Nat Commun. 2026 Aug 18;17:8569. doi: 10.1038/s41467-026-76542-z (PMC13486753; doi:10.1038/s41467-026-76542-z)
Supplement: Supplementary file 2 [file 41467_2026_76542_MOESM2_ESM.pdf]

Description of additional supplementary files

Title: Supplementary Data 1 (Supplementary_data_1.xlsx)

Description: Source data underlying all supplementary figures. The workbook contains separate worksheets corresponding to each supplementary figure, with each worksheet providing the numerical data used to generate the associated figure panels.
