# Supplementary material for: Tracking funding disparities in global health aid with machine learning
Source: Nat Commun. 2026 Aug 18;17:8569. doi: 10.1038/s41467-026-76542-z (PMC13486753; doi:10.1038/s41467-026-76542-z)
Supplement: Supplementary file 3 [file 41467_2026_76542_MOESM4_ESM.pdf]

nature portfolio

Corresponding author(s): Stefan Feuerriegel

Last updated by author(s): May 29, 2026

Reporting Summary

Nature Portfolio wishes to improve the reproducibility of the work that we publish. This form provides structure for consistency and transparency in reporting. For further information on Nature Portfolio policies, see our Editorial Policies and the Editorial Policy Checklist.

Statistics

For all statistical analyses, confirm that the following items are present in the figure legend, table legend, main text, or Methods section.

n/a	Confirmed	
☐	☒	The exact sample size (n) for each experimental group/condition, given as a discrete number and unit of measurement
☐	☒	A statement on whether measurements were taken from distinct samples or whether the same sample was measured repeatedly
☐	☒	The statistical test(s) used AND whether they are one- or two-sided<br>*Only common tests should be described solely by name; describe more complex techniques in the Methods section.*
☐	☒	A description of all covariates tested
☒	☐	A description of any assumptions or corrections, such as tests of normality and adjustment for multiple comparisons
☐	☒	A full description of the statistical parameters including central tendency (e.g. means) or other basic estimates (e.g. regression coefficient) AND variation (e.g. standard deviation) or associated estimates of uncertainty (e.g. confidence intervals)
☐	☒	For null hypothesis testing, the test statistic (e.g. F, t, r) with confidence intervals, effect sizes, degrees of freedom and P value noted<br>*Give P values as exact values whenever suitable.*
☒	☐	For Bayesian analysis, information on the choice of priors and Markov chain Monte Carlo settings
☒	☐	For hierarchical and complex designs, identification of the appropriate level for tests and full reporting of outcomes
☐	☒	Estimates of effect sizes (e.g. Cohen's d, Pearson's r), indicating how they were calculated

Our web collection on statistics for biologists contains articles on many of the points above.

Software and code

Policy information about availability of computer code

Data collection	OECD Creditor Reporting System data were downloaded from the OECD Data Explorer. Disease burden estimates were obtained from the Global Burden of Disease study. Population data were obtained from the World Bank. Non-English project descriptions were translated using the Google Translate API. HTTP/API requests were handled using requests (≥2.31) and aiohttp (≥3.9), with nest_asyncio (≥1.6) for asynchronous execution. Language detection was performed using langdetect (≥1.0.9) and langid (≥1.1.6). Project classification was performed using Llama-3.1-70B-Instruct-Turbo via the Together AI API.
Data analysis	Analyses were conducted in Python (3.12.10) using numpy (≥1.26), pandas (≥2.2), polars (≥1.0), scipy (≥1.13), scikit-learn (≥1.5), and Jinja2 (≥3.1). Statistical analyses included Spearman rank correlations and two-sided Spearman rank tests implemented with scipy. Classification validation used accuracy, precision, recall, F1-scores, Cohen’s kappa, and label consistency metrics implemented with scikit-learn and custom Python code. Data visualization and mapping used matplotlib (≥3.8), seaborn (≥0.13), cartopy (≥0.23), and geopandas (≥0.14). Custom code for preprocessing, classification, validation, statistical analysis, robustness checks, and figure generation is available at https://github.com/forsterkerstin/tracking-health-aid-disparities.

For manuscripts utilizing custom algorithms or software that are central to the research but not yet described in published literature, software must be made available to editors and reviewers. We strongly encourage code deposition in a community repository (e.g. GitHub). See the Nature Portfolio guidelines for submitting code & software for further information.

Data

Policy information about availability of data

All manuscripts must include a data availability statement. This statement should provide the following information, where applicable:

- Accession codes, unique identifiers, or web links for publicly available datasets
- A description of any restrictions on data availability
- For clinical datasets or third party data, please ensure that the statement adheres to our policy

The processed data generated in this study have been deposited in the Open Science Framework (OSF) repository at \url{https://osf.io/ce3q2/}. Source data are provided with this paper. The raw aid data used in this study are available from the OECD Creditor Reporting System at \url{https://data-explorer.oecd.org/}. The disease burden data used in this study are available from the Global Burden of Disease study \cite{james-2018, murray-2022}. Population data used to compute per capita aid are available from the World Bank at \url{https://data.worldbank.org/indicator/SP.POP.TOTL}.

Research involving human participants, their data, or biological material

Policy information about studies with human participants or human data. See also policy information about sex, gender (identity/presentation), and sexual orientation and race, ethnicity and racism.

Reporting on sex and gender	This study did not involve human participants or human data.
Reporting on race, ethnicity, or other socially relevant groupings	This study did not involve human participants or human data.
Population characteristics	This study did not involve human participants or human data.
Recruitment	This study did not involve human participants or human data.
Ethics oversight	This study did not involve human participants or human data.

Note that full information on the approval of the study protocol must also be provided in the manuscript.

Field-specific reporting

Please select the one below that is the best fit for your research. If you are not sure, read the appropriate sections before making your selection.

☐ Life sciences ☒ Behavioural & social sciences ☐ Ecological, evolutionary & environmental sciences

For a reference copy of the document with all sections, see nature.com/documents/nr-reporting-summary-flat.pdf

Behavioural & social sciences study design

All studies must disclose on these points even when the disclosure is negative.

Study description	This is a quantitative observational study using existing datasets from the OECD Creditor Reporting System, the Global Burden of Disease study, and the World Bank. We developed and validated a machine learning pipeline to classify official development assistance projects by disease category and analysed the alignment between disease-specific aid and country-level disease burden.
Research sample	The study sample comprised official development assistance projects from the OECD Creditor Reporting System for 2000-2022 and country-level disease burden estimates from the Global Burden of Disease study for 2000-2021. The analysis focused on 157 recipient countries classified as least-developed, lower-middle-income, or upper-middle-income countries. No human participants were recruited.
Sampling strategy	No participant sampling was performed. We included all eligible OECD CRS project records from 2000-2022 with valid textual descriptions, funding amounts, recipient-country information, and year information. The final cleaned dataset included 3,713,935 projects after excluding invalid records. No statistical sample size calculation was performed because the analysis used the full eligible dataset rather than a sampled participant cohort.
Data collection	No primary data collection was performed. OECD CRS project data, Global Burden of Disease estimates, and World Bank population data were obtained from existing databases. Project text was processed computationally, including language detection, translation of non-English descriptions, and classification by a large language model. No participants or researchers were involved in data collection from individuals, and blinding to experimental conditions was not applicable.
Timing	OECD CRS project data covered 2000-2022. Global Burden of Disease estimates used for comparisons with disease burden covered 2000-2021, which was the latest available period at the time of analysis. World Bank population data were used for the corresponding years to compute per capita aid.

Data exclusions	We excluded invalid OECD CRS project records with missing textual descriptions, missing funding amounts, or negative funding amounts. The initial dataset contained 4,726,638 projects; 1,012,703 records were excluded, leaving 3,713,935 projects for analysis.
Non-participation	No human participants were involved.
Randomization	No participants were allocated to experimental groups, and no intervention was conducted. Randomization was therefore not applicable.

Reporting for specific materials, systems and methods

We require information from authors about some types of materials, experimental systems and methods used in many studies. Here, indicate whether each material, system or method listed is relevant to your study. If you are not sure if a list item applies to your research, read the appropriate section before selecting a response.

Materials & experimental systems

n/a	Involved in the study	
☒	☐	Antibodies
☒	☐	Eukaryotic cell lines
☒	☐	Palaeontology and archaeology
☒	☐	Animals and other organisms
☒	☐	Clinical data
☒	☐	Dual use research of concern
☒	☐	Plants

Methods

n/a	Involved in the study	
☒	☐	ChIP-seq
☒	☐	Flow cytometry
☒	☐	MRI-based neuroimaging

Plants

Seed stocks	This study did not involve plants, seed stocks, or other plant material.
Novel plant genotypes	This study did not involve plants, seed stocks, or other plant material.
Authentication	This study did not involve plants, seed stocks, or other plant material.
