# Supplementary material for: Tracking funding disparities in global health aid with machine learning
Source: Nat Commun. 2026 Aug 18;17:8569. doi: 10.1038/s41467-026-76542-z (PMC13486753; doi:10.1038/s41467-026-76542-z)
Supplement: Supplementary file 4 [file 41467_2026_76542_MOESM5_ESM.pdf]

nature portfolio

Peer Review File

Tracking funding disparities in global health aid with machine learning

Corresponding Author: Professor Stefan Feuerriegel

This file contains all reviewer reports in order by version, followed by all author rebuttals in order by version.

Version 0:

Reviewer comments:

Reviewer #1

(Remarks to the Author)
The article utilizes large language models to categorize development assistance for health into 17 major disease areas and examine the adequacy of provided funding relative to individual country disease burden within income groups to determine funding gaps. A key finding is that in several countries based on the disease burden and aid received, there are disparities/misalignment. The effort to apply the increasingly ubiquitous machine learning approach to this area of work while not novel is commendable. There are, however, specific areas of concern that I highlight below for the authors' attention.

Major comments
-The authors' assessment of the existing literature is inaccurate. IHME's work is comprehensive and includes several health areas as well as diseases (non-communicable diseases, neglected tropical diseases etc. in addition to the typical ones referenced). Additionally, the period and data records covered in the Financing Global Health study exceed what is included in this current work. It leverages a longer time series of CRS data (1990 through present) and also incorporate additional data sources on private foundations and NGOs which is missing from the current work.

-While matching disease burden to development assistance support received might be a rational way to allocate development assistance support, this type of analysis has received criticism (see : Voigt & King (2017) Out of Alignment? Limitations of the Global Burden of Disease in Assessing the Allocation of Global Health Aid https://pubmed.ncbi.nlm.nih.gov/29731809/). The authors mention some of these in their limitation section. However, given the centrality of this kind of analysis to the study, the authors should address/acknowledge the challenges with such analysis earlier on in the manuscript and more comprehensively than what is currently included as a sentence in the limitation section of the manuscript.

-The authors' use of funding gap to discuss the analysis they completed is questionable. While they note that the term is not used to imply the adequacy of the volume of aid support but its relativity to country disease burden, this is still problematic because any such gap assessment must consider all sources of funding -government, household and donor – for the health sector otherwise the analysis promotes the assumption that donor funding is the primary source of funding in the health sector across these countries which is inaccurate. Recommend switching to describing the analysis more in terms of alignment of aid with country needs specifically and not as funding gap as currently described since that term connotes additional data and analysis that is not currently included.

-The authors detail out how multi-focused projects were handled in the analysis by splitting disbursements across the various disease areas identified. This approach is similar to what is typically done using the keyword approach. What is missing is how the authors also addressed resource flows that may have passed through multiple agencies as well as an acknowledgement in the limitation that approach relies primarily on the descriptions provided in the CRS database and so the degree to which allocation or utilization of the resources in practice differed from what was detailed in the description the conclusion in the analysis could be misleading.

-The authors should also include the limitations of the CRS database in the manuscript including that it has limited coverage of private foundations contributions and so the findings from the study should be caveated as primarily applicable to government sourced development assistance for health.

-One of the key areas of misalignment the study finds is NCDs. It is therefore unclear to me why the figure for the analysis highlighting this disparity is included in the appendix instead of in the main manuscript text. Recommend moving it up if possible.

-The authors also indicate that they use google to translate foreign language descriptions to English in order to leverage the large language model. While this may be reasonable the authors should acknowledge the error that this translation could introduce into the study.

-Lastly the current framework, provides little information on who is providing funds so while it is helpful to know the countries receiving resources and for what, it is limited in terms of who can be held accountable for the disparities highlighted in the findings which limits how the data can be acted upon.

(Remarks on code availability)
While I did go to the website and scan through the hierarchy and file system of the project. I did not run the code line by line. The structure set up aligns with the text description in the manuscript.

Reviewer #2

(Remarks to the Author)
Overall comments:
This was a wonderful research paper addressing the timely topic of funding disparities in public health. The study used new methodologies, artificial intelligence (AI) and machine learning, in a unique and novel way to comprehensively evaluate funding disparities. Strengths of AI and machine learning were leveraged well in this study, particularly with this method's ability to assess large amounts of data, 3.7 million projects in this case, in an efficient manner.
Major Comments:
L225 While the link between certain diseases, such as HIV and TB, was briefly addressed, consider expanding on this in the discussion. Using specific disease categories based on the GBD framework allows for clear interpretation and aligns with global priorities. However, these diseases do not always exist in isolation. For example, HIV increases the risk of TB, and TB can accelerate the progression of HIV. Since some projects fund only one disease—even when it may influence the development or progression of another—the discussion could be strengthened by considering the potential impact of these interrelationships.
L312 It would be helpful to have a flow chart. For example, 4.7 million projects 3.7 million projects X value for NCDs and Y value for CMNNDs, Z value for each disease, etc. It would also be helpful to see at which point validation occurred. Furthermore, would it be appropriate to do any sensitivity analyses with imputation as a significant portion of the data were removed? The discussion should address if there are potential biases due to the missing data.
L320 Would you be able to expand on how the 17 major categories of CMNNDs and NCDs were selected? While the Global Burden of Disease study was cited, 354 causes are listed in that study. It looks like the 17 categories were selected from the Level 2 causes of GBD, but it should be explained how they were selected. For example, "other infectious diseases" is a Level 2 cause that would be a CMNND, but this was not included In Supplementary Table S1.
L325 It was discussed that project funding was distributed equally among all different projects, but would this be an accurate assumption? Do these projects pre-specify what proportion of the funding would be allotted to what disease state, or would this be up to the applicant's or funder's discretion?
L362 Would you be able to expand on why the number of 2,060 was chosen and how they were chosen to validate the model? How was it determined that this was a sufficient value?
Minor Comments:
L70 Thank you for all the details in the supplement which discuss the validation of the language learning model (LLM) and how it was used. Along with the provided code, it helps improve the reproducibility of the study and demonstrates the robustness of the study.
L71 While it was noted that the remainder of the projects were related to other sustainability targets like climate action, could this be expanded upon? What were these sustainability targets, and if these projects could have spillover effects on NCD and CMMND burden, could this be addressed in the discussion?
L81 The statement for "reflecting long-standing global priorities" should be cited or can be removed as it may be more appropriate for the discussion section.
Figures 2 and 3 do a great job of demonstrating the differences in magnitude of funding across different disease states and how funding has changed over time.
L94 This paragraph for the results discussing the ODA distribution by disease and geography was harder to follow. Simplifying this paragraph to the key points would be helpful. For example, what were the overall trends for CMNNDs and NCDs? Additionally, why was the figure for CMNND depicted in the main manuscript while the figure for NCDs were in the Supplement? Furthermore, Figure S3 could benefit from making the font and images larger, as it is a bit difficult to read.
Figure 6 was nicely done. It clearly demonstrates the correlation between aid and burden. For the Spearman rank coefficients with the correlations and the alignments, i.e. "0.3 to 0.7, moderate alignment," would you be able to provide a citation to support the interpretation of the correlation coefficients? Based on the literature, a variety of interpretations on strength of the association and the correlation coefficient could be made.
• Mukaka MM. Statistics corner: A guide to appropriate use of correlation coefficient in medical research. Malawi Med J. 2012 Sep;24(3):69-71. PMID: 23638278; PMCID: PMC3576830.
• Schober P, Boer C, Schwarte LA. Correlation Coefficients: Appropriate Use and Interpretation. Anesth Analg. 2018 May;126(5):1763-1768. doi: 10.1213/ANE.0000000000002864. PMID: 29481436.

L240 Rather than persistent underfunding, would it be more appropriate to say that there was a significant gap in the funding attributed to CMNNDs compared to NCDs or relative funding? Overall, the funding for NCDs has increased over time.

(Remarks on code availability)
The instructions for the code appear to be very clear and easy to follow. It is well-aligned with the methods section of the text.

Reviewer #3

(Remarks to the Author)
I co-reviewed this manuscript with one of the reviewers who provided the listed reports. This is part of the Nature Communications initiative to facilitate training in peer review and to provide appropriate recognition for Early Career Researchers who co-review manuscripts.

(Remarks on code availability)

Reviewer #4

(Remarks to the Author)
Thank you for creating a pipeline to address an important issue. There are important issues to address concerning the methods employed. Good luck with your revisions.

Comments, besides Methods

The authors equate alignment between aid and disease burden (as measured by DALYs) with appropriate or desirable aid allocation. While this is a defensible premise, it is controversial. The manuscript would benefit from a more nuanced discussion of alternative justifications for aid allocation (e.g., cost-effectiveness, absorptive capacity, co-benefits) and why DALYs (almost alone) are here used as a proxy for “need.” The authors rightly note in the limitations that other factors (e.g., governance, domestic investment, health infrastructure) affect both funding decisions and outcomes. A short paragraph in Discussion to situate DALY-misaligned aid in this broader context would improve the policy relevance.

The funding gap metric is innovative but the authors should clarify that it captures relative disparity within income groups, not necessarily absolute underfunding. A short explanation in the main text or figure captions (e.g., Figure 8) would aid interpretability.

Comments on Methods

The machine learning classification is central to the paper, yet the authors provide no uncertainty estimates (e.g., confidence scores or inter-model variation). Even a brief exploration of classification robustness (e.g., for ambiguous descriptions, multilingual projects, or short texts) would increase the credibility of the pipeline and highlight where future refinements are needed. It should also be called just that: pipeline (the term Framework is not appropriate).
The LLM classification accuracy is good, but the validation dataset (n=2,060) is small compared to the project universe. Were the validation projects sampled randomly? Stratified? Were inter-annotator agreement or category-level confusion matrices assessed? The authors should address both intra-model variability (e.g. variability across different runs of the same model) and inter-rater reliability (if human annotators are used for validation).

LLMs are nondeterministic by default: responses can vary between runs unless a low temperature and seed are fixed. This means any label assigned to a project could vary if the prompt were re-run, and such variability can affect downstream conclusions, especially when used at scale and in critical settings like ODA allocation, see e.g. Scaling Instruction-Finetuned Language Models (https://arxiv.org/abs/2210.11416) Re-run classification on a random 1 per cent subset with different seeds/temperatures to quantify variability. Alternatively, perform bootstrap sampling or Monte Carlo dropout-style ensemble to capture label uncertainty.

The validation set (2,060 manually labeled projects) is impressive in scale, but it is unclear whether more than one annotator was used per item. If not, it is hard to assess how subjective or stable the disease labelling process is, especially since categories can overlap. It is in the paper (e.g. Figure 1 caption) unclear if the classification is a partition or not: it seems not, but at the same time a process is described for how to divide financial funds that seems to be based on a previous partition. For inter-rater variability, use Cohen’s Kappa (for two annotators) or Krippendorff’s alpha or Fleiss’ Kappa (for more). Even better, report category-wise confusion matrices or inter-annotator agreement across disease groups.

The most important shortcoming lies in the context window of the LLMs used. The two main problems are the context window truncation (batch size/input length) and semantic misinterpretation due to naive concatenation and lack of negation handling. These are well-known but often overlooked issues in applying LLMs to large-scale classification tasks, especially in noisy real-world text like ODA project descriptions.

First, the authors mention concatenating the project title, short description, and long description into a single input string per project for classification. However, the Llama-3.1-70B-Instruct model has a context window (e.g., 8,192 tokens, possibly

more with Together.ai's infrastructure, but still finite), and attention degrades with position. Thus:

• If the combined input exceeds the context window, later portions may be truncated silently or receive less attention.
• Even well within the limit, studies have shown that LLMs prioritise earlier tokens, especially for tasks where the prompt does not explicitly instruct “attend to all parts.”
• Important information at the end (e.g., secondary disease focus) may be ignored.
• Classifications may be biased toward the beginning of the description.

The authors should consider a chunk-based classification strategy, where project descriptions are split into segments (e.g., title, short, and long text or sentence-level blocks), each classified separately, and the final disease assignment is aggregated from segment predictions. This would mitigate context window truncation issues and reduce positional bias. Additionally, the prompt could be revised to explicitly instruct the model to ignore diseases that are mentioned only in exclusionary or background contexts. A short validation of this approach on a subsample would be a valuable robustness check.

Second, by blindly concatenating fields, the authors risk semantic errors like misinterpreting negations (e.g., “This project must not address HIV”) as positive signals. They also risk failing to account for discourse structure, e.g., distinguishing goals from exclusions, comparisons, or context. This is a well-known issue in clinical and biomedical NLP, where negation detection is critical. It is applicable here because development aid projects often describe what they avoid, no longer fund, or previously included.

Suggested mitigation strategies:
• Use sentence-level classification, or at least chunking, to reduce context overload and preserve syntactic cues.
• Explicitly prompt the model to handle negation.
• Run a negation detection preprocessor (like NegEx, pyConTextNLP, or spaCy’s dependency parsing) before classification.

Suggestion for a revised prompt with explicit negation and scope handling:

You are an expert health policy assistant. Your task is to identify which disease categories are positively targeted by this development aid project, based on its description.
Read the project description carefully. ONLY include disease categories that are explicitly targeted, supported, or addressed in the project. DO NOT include diseases that are:
- Mentioned as excluded
- Mentioned as not funded
- Mentioned only in background context
- Described as 'not a focus' or 'not covered'

You may assign one or more of the following 17 disease categories:
[List of categories]

Project Description:
"""
[concatenated title + descriptions]
"""

Answer as a list of disease categories (by number and name). Use "Other" or "General Health" if no specific disease is addressed.

This type of prompt nudges the model toward conservative, scoped classification and helps prevent false positives due to negation. If the authors use this revised prompt and re-run on even a small random subset (e.g. 500 projects), they could report the change in classification outcomes, which in itself would be very informative.

Minor issues

Use “DALYs per 100,000” consistently. In some places (e.g., Figure 7), the denominator is unclear.

In the Figure 5 caption, the phrase “may indicate underfunding” could be softened or clarified (e.g., “may reflect underfunding relative to burden”).

In the Abstract, a trend (as in “rising burden”) is not in itself a burden.

In Figure 2a, Mental disorders should be deleted.

For Spearman, you should replace r by rho (the Greek letter), r is used for Pearson. Spearman’s ρ captures monotonic relationships, including non-linear ones, whereas Pearson’s r captures only linear associations. The comment in the

manuscript should be corrected accordingly.

On page 12, “$p > 0.05$” should be “$p < 0.05$”.

Parts of “Sensitivity analysis” should be moved to Methods.

The beginning of Discussion should be deleted, no summary is necessary there.

(Remarks on code availability)
README file is informative and all links are working. Instructions are adequate, and the data sharing works just fine. There was no reason for me to run the code, only to inspect it to guide my detailed comments to the authors.

Version 1:

Reviewer comments:

Reviewer #1

(Remarks to the Author)
The authors use large language methods to examine the alignment of aid disbursements with disease burden. After revisions, the manuscript provides a good addition to the literature by providing more evidence on a longstanding question in global health financing concerning the allocation of aid. The manuscript text and methodology have all been revised substantially to address concerns I previously raised.

(Remarks on code availability)
Again, I did not run the code line by line, but the layout is aligned with the analysis completed from my initial review.

Reviewer #2

(Remarks to the Author)
The authors have addressed our comments very well.

(Remarks on code availability)

Reviewer #3

(Remarks to the Author)
I co-reviewed this manuscript with one of the reviewers who provided the listed reports. This is part of the Nature Communications initiative to facilitate training in peer review and to provide appropriate recognition for Early Career Researchers who co-review manuscripts.

(Remarks on code availability)

Reviewer #4

(Remarks to the Author)
Thank you for addressing my comments, I appreciate the work put in, and about half of my points were addressed just fine, what remains of them is below. Please note that not every single point needs to be thoroughly addressed for me to consider your work sufficient, so do not despair.

R4.1
While you added acknowledgment of limitations, the fundamental issue remains as you still use DALY alignment as the primary analytical model throughout. The expanded discussion may be relegated to limitations rather than integrated into the core interpretation.

R4.2
Your changed terminology from "funding gap" to "alignment/misalignment" is cosmetic. The underlying methodological issue that you are only measuring donor contributions without domestic/household spending remains.

R4.3
You did substantial work here. What remains to consider:
- Only 1% subset for reproducibility may not capture rare categories adequately.
- No confidence intervals on final disease allocations themselves.
- For the LLM comparison: gpt-4.1-mini-2025-04-14 seems like an odd choice?

R4.4
You did substantial work here. What remains to consider:

- How exactly were the 2,060 selected? Was it stratified random sampling by category? By funding amount? By donor?
- While Fig S12 shows convergence, it does not address whether 50 samples per category is sufficient for rare categories with high within-category variance.
- Second annotator added after the fact seems like a response to my review rather than original design. Were discrepancies adjudicated? How?

R4.7 (IMPORTANT)
The chunk-based approach performed worse, and you can NOT interpret this as "truncation or positional bias did not materially affect results." This is backward logic. Poor performance of chunks could mean:
- The chunking implementation was flawed.
- The chunks lost critical context.
- The prompt design for chunks was inadequate.

You also only tested on the validation set (n=2,060), not the full dataset. The validation set may not represent the full distribution of text lengths and structures. There was no positional bias analysis: you did not actually test whether disease mentions at the end of descriptions are classified differently than those at the beginning.

The negation handling resulted in no improvement as I understand it (accuracy 0.74 vs 0.78 original)!?

NegEx is a rule-based system designed for clinical notes. Development aid descriptions have different linguistic structures. The 0.8% finding may be a false negative, missing implicit negations or complex discourse structures.

Sample representativeness was only tested on projects already flagged as having negations. You should also test on random sample to see if LLM creates false positives from missed negations. The negation-aware prompt performed worse from which you cannot conclude negation is not a problem. The optimised prompt may be too restrictive, manual annotators may have made errors on negated cases, plus the 500-sample validation is too small to detect category-specific patterns.

(Remarks on code availability)

Version 2:

Reviewer comments:

Reviewer #4

(Remarks to the Author)
I am very impressed with the author revisions in this rebuttal round.

Most importantly, they acknowledged the interpretive error regarding chunk-based analysis and removed the unjustified claim. They added multiple new analyses that strengthen the robustness assessment. Critically, they have reframed their claims more cautiously, acknowledging inherent limitations of LLM-based classification rather than asserting the absence of problems.

The revised Discussion now appropriately positions this work as an advance over simpler keyword-based methods while transparently acknowledging sensitivity to model choice, input structure, and positional information. The validation remains limited in scope, particularly for rare categories, but this limitation is now explicitly disclosed.

While no validation can fully capture all sources of classification uncertainty in a 3.7M-project corpus, the authors have followed current best practice: manual annotation with high inter-rater reliability, robustness checks across multiple models and seeds, transparent reporting of uncertainty intervals, and honest acknowledgment of limitations.

I recommend acceptance with the understanding that this represents a useful methodological contribution to global health aid tracking, with appropriate caveats about LLM-based classification that future work should continue to address.

(Remarks on code availability)
The OSF data and README file are both fine and the procedure is clear. The GitHub page would be served well by some About info, at the moment there is only pragmatic and procedural info, no semantic or contextual info.

Open Access This Peer Review File is licensed under a Creative Commons Attribution 4.0 International License, which permits use, sharing, adaptation, distribution and reproduction in any medium or format, as long as you give appropriate credit to the original author(s) and the source, provide a link to the Creative Commons license, and indicate if changes were made.
In cases where reviewers are anonymous, credit should be given to 'Anonymous Referee' and the source.
The images or other third party material in this Peer Review File are included in the article's Creative Commons license, unless indicated otherwise in a credit line to the material. If material is not included in the article's Creative Commons license and your intended use is not permitted by statutory regulation or exceeds the permitted use, you will need to obtain permission directly from the copyright holder.
To view a copy of this license, visit https://creativecommons.org/licenses/by/4.0/
